# Global patterns and drivers of extinction risk in island endemic terrestrial vertebrates

**DOI:** 10.1126/sciadv.aef9781

**Published:** 2026-08-05

**Authors:** Yuyang Zhang, Jiang Wang, Yanxia Li, Chuanwu Chen, Yanping Wang

**Affiliations:** ^1^ Laboratory of Island Biogeography and Conservation Biology, College of Life Sciences, Nanjing Normal University, Nanjing 210023, China.; ^2^ State Key Laboratory of Animal Biodiversity Conservation and Integrated Pest Management, Institute of Zoology, Chinese Academy of Sciences, Beijing 100101, China.

## Abstract

Island endemic terrestrial vertebrates, shaped by long-term isolation and ecological specialization, are particularly vulnerable to extinction pressure in the Anthropocene. However, cross-taxon extinction patterns and their drivers remain poorly understood. Here, we compiled a global dataset of 31,865 terrestrial vertebrates, including more than 7500 island endemic species, to evaluate the patterns and drivers of extinction risk in island endemics, in comparison with continent endemics and dual-range species. We found that island endemics face significantly higher extinction risk than non-island endemics, with 3.1% extinct and 34% currently threatened since 1500 CE. Extinction risk among island endemics is unevenly distributed and taxonomically structured, tightly linked to invasive species, overexploitation, and climate change. Compared with continent endemics and dual-range species, their elevated risk is closely associated with small range size and habitat specialization, while biological traits, climate, and anthropogenic pressures also exert taxon-specific effects. These findings explain the unique vulnerability of island endemic vertebrates and inform conservation priorities across island ecosystems.

## Teaser

Island endemics face higher extinction risk due to taxon-specific traits, environmental factors, and human impacts.

## INTRODUCTION

Biodiversity loss represents one of the most critical environmental challenges of the 21st century and is a hallmark of the proposed sixth mass extinction (*1*, *2*). Despite ongoing global conservation efforts, extinction rates during the Anthropocene remain alarmingly high, now estimated to be orders of magnitude greater than natural background rates (*1*, *3*–*5*). Unlike previous mass extinctions dominated by naturally geological or astrophysical events, the current biodiversity crisis is accelerated by human activities including habitat conversion, human overexploitation, invasive species, and climate change, progressively eroding planetary evolutionary heritage and ecosystem resilience (*5*–*7*). Accordingly, identifying the most vulnerable lineages and regions, along with elucidating the mechanisms underpinning their vulnerability, constitutes a fundamental challenge in mitigating the ongoing biodiversity crisis (*8*, *9*).

Islands have long been recognized as epicenters of the global extinction crisis (*10*, *11*). Although islands constitute just 6.7% of the Earth’s land surface (*12*), they harbor more than 20% of global biodiversity and have experienced ∼70% of documented species extinctions since 1500 CE (*13*–*15*). Historical extinctions reflect a fundamental evolutionary paradox on islands. Prolonged isolation often leads to the “island syndrome,” a suite of adaptations including shifts in body size, reduced reproductive output, and the loss of defensive traits (*16*–*18*). While these adaptations enhance survival and reproductive success in stable, isolated island environments (*19*), they concurrently increase species vulnerability to emerging anthropogenic pressures such as habitat alteration, human overexploitation, and introduced species (*16*, *20*).

Terrestrial vertebrates provide a particularly suitable model system for investigating extinction processes on islands, owing to their well-documented ecological functions and the relatively comprehensive global data available on their traits, distributions, phylogenetic relationships, and conservation status (*21*). Among these taxa, island endemic terrestrial vertebrates (hereafter abbreviated as “island endemics”) are especially vulnerable because their entire distributions are restricted to insular environments. They typically occupy extremely small geographic ranges and lack demographic or spatial buffering outside island systems (*10*). Compared with continent endemic terrestrial vertebrates (hereafter abbreviated as “continent endemics”) and dual-range species occurring in both continental and insular environments, island endemics exhibit a disproportionately high sensitivity to habitat loss, invasive species, and other intrinsic and extrinsic pressures (*22*, *23*), making them a key group for understanding extinction dynamics in island ecosystems.

For species on islands, extinction risk stems from the interaction of intrinsic traits and extrinsic pressures (*24*). Building on early global analyses that identified life-history traits such as body size, reproductive rate, and foraging modes as predictors of extinction vulnerability (*25*–*27*), studies focusing on island faunas have provided more taxon-specific insights. For instance, island endemic birds with larger body sizes and higher hand-wing indices exhibit higher extinction risk (*22*, *28*). Similarly, threatened mammalian lineages on islands often display extreme shifts in body size and reduced metabolic rates (*29*, *30*). These adaptive characteristics, while advantageous in historically stable island environments, may simultaneously increase vulnerability to rapidly intensifying anthropogenic pressures (*30*, *31*). Beyond intrinsic traits, an increasing number of studies have incorporated environmental gradients to explain variation in extinction risk across islands. Climatic conditions have been proved to shape the diversity of mammals on islands worldwide (*32*) and alter demographic rates, phenology, distribution, and physiological performance of insular vertebrate assemblages (*33*, *34*). At the same time, nonclimatic geographic gradients such as elevation can exert contrasting effects across different taxonomic groups (*35*–*37*). The unprecedented rapidity of environment changes during the Anthropocene has created a temporal mismatch, in which the pace of environmental alteration vastly outstrips the capacity of species to adapt through natural selection, thereby critically determining extinction outcomes (*38*, *39*). More recently, the availability of global anthropogenic pressure datasets, such as the human footprint index and land use conditions, has enabled researchers to quantify the cumulative impacts of human activities on biodiversity (*40*). Case studies from New Zealand confirm that extrinsic threats need to be considered properly, as they may outweigh key life-history traits such as clutch size and body size (*6*, *41*). Human activities have repeatedly driven insular ecosystems beyond their ecological thresholds (*42*). Exotic species that accompanied human voyages, such as rats (*Rattus* spp.) and cats (*Felis catus*), conversion of native habitats for agriculture, and unsustainable harvesting have collectively precipitated hundreds of extinctions on islands (*20*, *43*, *44*). These pressures seldom act in isolation. Habitat modification often facilitates biological invasions, which, in turn, can disrupt food webs and amplify the impacts of climate change via trophic cascades (*45*). Therefore, advancing predictive frameworks requires an integrated approach to better understand the combined effects of biological traits, ecological contexts, and anthropogenic pressures on extinction dynamics across insular ecosystems.

Island endemics should be the focus of conservation because their local extinction on islands means complete global extinction. As key functional components of island ecosystems, island endemics play critical roles in energy transfer, food-web regulation, and the coupling of ecological processes (*46*–*48*). While multiple studies have examined correlates of extinction risk, most previous studies have focused on single taxonomic groups or regional island systems, and few have simultaneously evaluated biological traits, environmental gradients, and anthropogenic pressures within a unified comparative framework. This limitation has constrained systematic insights into the drivers of extinction risk in island endemic terrestrial vertebrates at a global scale.

Here, we compiled a comprehensive global database of terrestrial vertebrates that includes both island endemic and non-island endemic species to investigate the patterns and drivers of extinction risk on global scale. We addressed the following questions: (i) How does extinction risk differ between island endemics and non-island endemic species assemblages? (ii) What are the multifaceted patterns of extinction risk in island endemics, encompassing geographic hotspots, phylogenetic nonrandomness across major taxonomic groups, and the variation of threat types across International Union for Conservation of Nature (IUCN) risk categories? (iii) What are the key intrinsic and extrinsic correlates that jointly drive extinction risk in island endemics? To address these questions, we applied an integrated phylogenetic comparative framework that enables a systematic assessment of how biological traits, ecological contexts, and external pressures collectively shape the extinction dynamics of island endemic terrestrial vertebrates worldwide.

## RESULTS

**Fig. 1.**
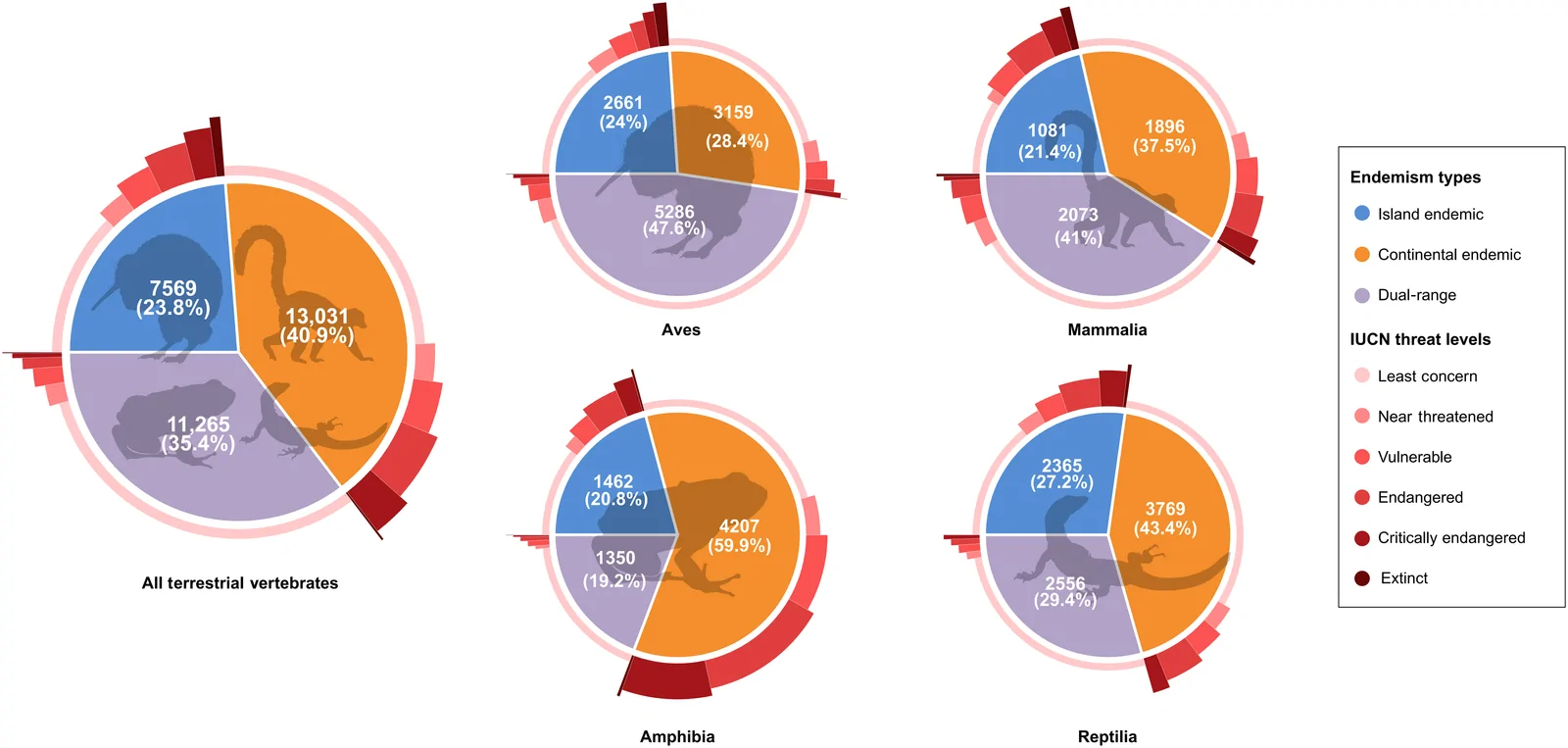
Distribution of endemism types in global terrestrial vertebrates. Nested donut charts showing the distribution of endemism types (inner ring) and IUCN Red List threat categories (outer segments) for 31,865 non-DD terrestrial vertebrate species. The large central chart displays results for all taxa combined, with smaller charts showing patterns separately for birds, mammals, amphibians, and reptiles. Numbers in the inner rings indicate species counts and percentages for each endemism category (island endemic, continent endemic, and dual-range). The outer segments represent the proportional composition of threat levels (LC = least concern, NT = near threatened, VU = vulnerable, EN = endangered, CR = critically endangered, and EX = extinct) within each endemism type. Source data are provided in data S2. Silhouettes shown in the panels depict representative island endemic species: *Apteryx australis* (Aves), *Lemur catta* (Mammalia), *Varanus komodoensis* (Reptilia), and *Mantella milotympanum* (Amphibia). All silhouettes are from PhyloPic (http://phylopic.org). The silhouettes of *A. australis* and *V. komodoensis* were created by S. Traver, whereas those of *L. catta* and *M. milotympanum* were created by F. Sayol. All images are available under the CC0 1.0 Universal Public Domain Dedication.

Overall, we compiled and retained 31,865 non–data-deficient (non-DD) terrestrial vertebrate species (data S1 and S2). Among them, 7569 (23.75%) are island endemics, dominated by Aves (2661), Reptilia (2365), Amphibia (1462), and Mammalia (1081), while 13,031 (40.89%) are continent endemics occurring only on continents and 11,265 (35.35%) are dual-range species occurring on both islands and continents (Fig. 1).

### Extinction risk and population trends

Island endemic terrestrial vertebrates exhibited markedly elevated extinction risk: Since 1500 CE, 236 (3.12%) species have gone extinct (EX), with a further 2574 (34.01%) species classified as threatened, including 622 (8.22%) as critically endangered (CR), 1030 (13.61%) as endangered (EN), and 922 (12.18%) as vulnerable (VU) according to the IUCN Red List (Fig. 1 and data S2). In contrast, only 72 (0.30%) continent endemic or dual-range species were classified as extinct. The combined proportion of threatened continent endemic or dual-range species from VU to CR categories was lower at 19.3% (Fig. 1 and data S2). A chi-square test confirmed significant differences in threat status between island endemics and non-island endemics (χ^2^ = 971.43, df = 1, *P* < 2.9 × 10^−213^), with island endemics showing disproportionately higher extinction risk (data S2 and table S3). The strength of this pattern varied among major vertebrate classes. Birds (χ^2^ = 602.34, *P* < 5.2 × 10^−133^), mammals (χ^2^ = 436.39, *P* < 6.6 × 10^−97^), and reptiles (χ^2^ = 537.84, *P* < 5.6 × 10^−119^) all showed highly significant differences, with island endemics consistently facing greater extinction risk since 1500 CE. Amphibians, however, showed no significant difference between island endemics and non-island endemics (χ^2^ = 0.65, *P* = 0.42). A finer-scale comparison among three categories (island endemics, continent endemics, and dual-range species) revealed that extinction risk was highest in island endemics, intermediate in continent endemics, and lowest in dual-range species across all vertebrate classes except amphibians (table S4).

Among island endemics, the majority of species are experiencing population declines, including 56.50% of amphibians, 47.73% of birds, 50.05% of mammals, and 25.75% of reptiles (table S5A). Only a small fraction (<6%) of island endemics show increasing populations. When considering only threatened species, declining trends dominate more strongly, reaching 89.36% in amphibians, 73.22% in birds, 82.18% in mammals, and 52.53% in reptiles (table S5B).

### Geographic concentration and taxonomic selectivity of extinction risk

**Fig. 2.**
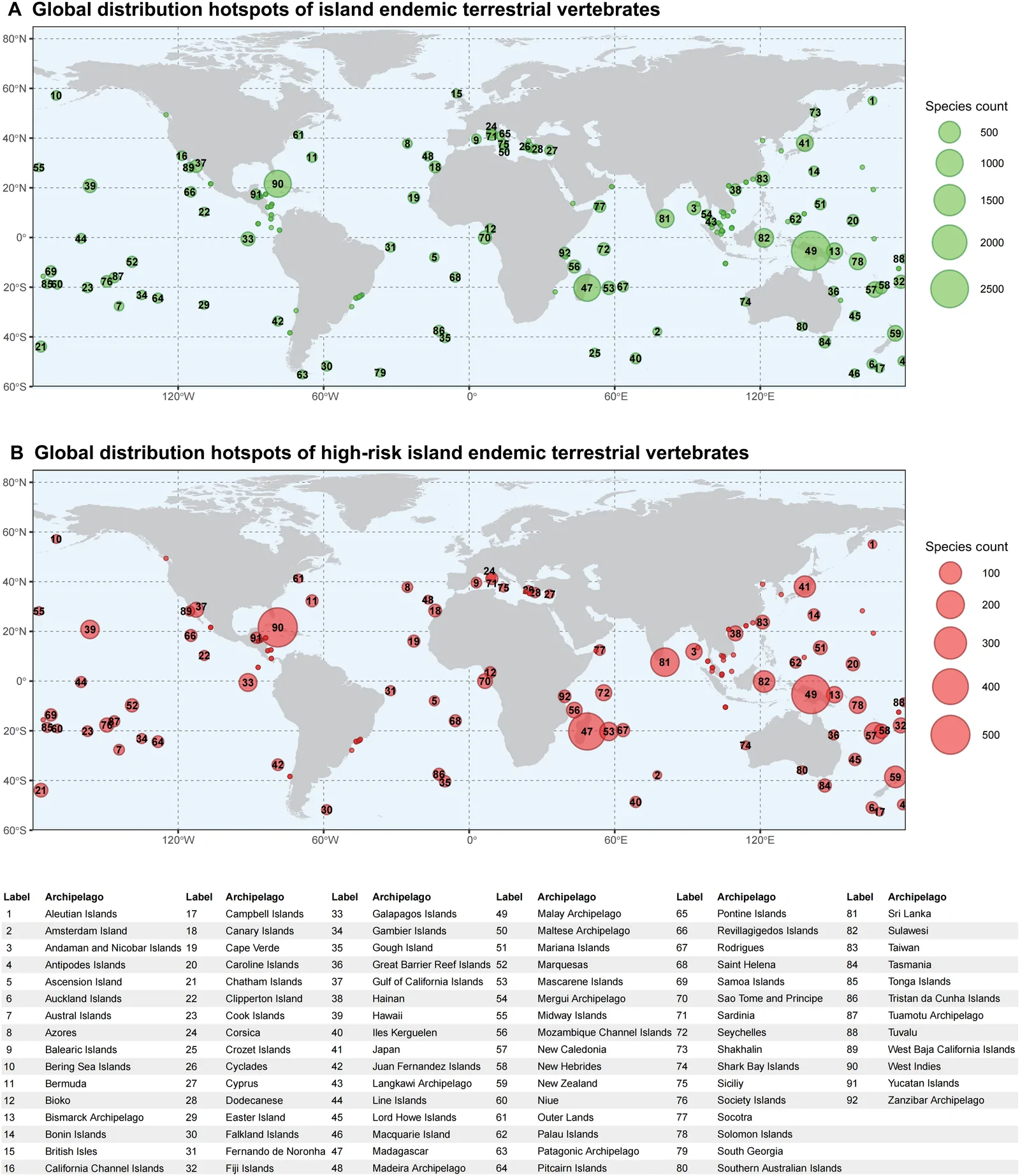
Global hotspots of island endemic terrestrial vertebrates and extinction risk. Panels show the global distribution of archipelago level concentrations of island endemic terrestrial vertebrates. Circle size is proportional to species richness within each archipelago, based on total island endemics (shown in green) in panel (**A**) and high-risk island endemics (shown in red) classified as EX, CR, EN, or VU in panel (**B**). Circles are located at the centroid of each archipelago’s largest island, with numeric labels correspond to archipelagos listed in the accompanying correspondence table shown as the legend. Island endemic species restricted to remote or isolated small islands that were not assigned to any archipelago are displayed as small points without numerical labeling. The global maps are displayed between 60°S and 85°N to focus on the primary latitudinal range of terrestrial vertebrate diversity and island systems and to minimize visual distortion. The base map was generated using the Natural Earth dataset (naturalearthdata.com) accessed through the R package rnaturalearth (version 1.0.1). Only nonpolitical landmass outlines were retained.

Island endemic terrestrial vertebrates are unevenly distributed across the globe. While a few endemic species occur on remote or isolated islands outside the major archipelagos, most are found within regions that vary in endemism (Fig. 2). Overall, the Malay Archipelago supports the largest number of island endemics (2767 species), followed by the West Indies (1004 species) and Madagascar (972 species). Hotspots of high extinction risk largely overlap with, but do not exactly match, these centers of endemism. The West Indies contain the greatest number of high-risk endemics (507 species), followed by the Malay Archipelago (490 species) and Madagascar (441 species). Smaller islands—including New Caledonia, Hawaii, and the Mascarene Islands, among others—carry disproportionately high conservation burden, with more than half of their endemics threatened despite their lower total species richness. Across birds, mammals, and reptiles, high-risk endemics tend to occur on large, species-rich archipelagos, while amphibians show a more geographically restricted pattern, with high-risk species concentrated in only a few archipelagos such as West Indies and Madagascar. Full regional rankings of island endemic richness and associated extinction risk for all terrestrial vertebrates and each of the four major classes are provided in data S4.

**Fig. 3.**
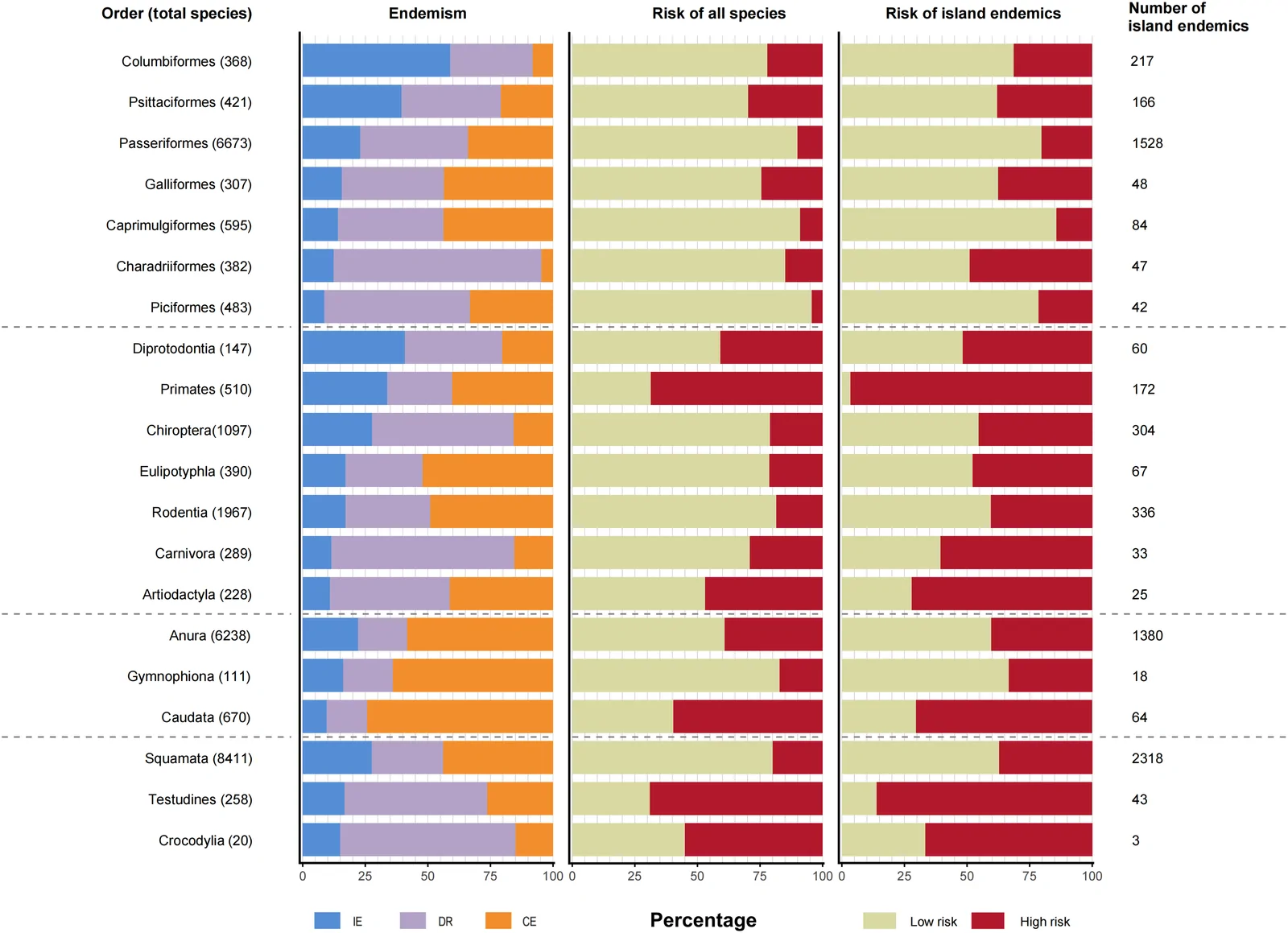
Contrasting patterns of island endemism and extinction risk across major vertebrate orders. Only orders with ≥20 species were included. Within each vertebrate class, the most species-rich orders were retained (birds and mammals: top seven orders; amphibians and reptiles: top three orders). For each order, stacked bars show the proportional composition of endemism types (island endemics, continent endemics, and dual-range species), the overall extinction risk of all species, and the extinction risk among island endemic species. Extinction risk was classified into high extinction risk (EX, CR, EN, and VU) and low extinction risk (NT and LC). Numbers at the right indicate the total number of island endemic species per order. Dashed horizontal lines separate vertebrate classes. Source data are provided in table S6. IE, island endemics; CE, continent endemics; DR, dual-range species.

Extinction risk among island endemics was taxonomically structured, showing clear nonrandom patterns across both orders and families. High concentrations of island endemics were found in Psittaciformes, Primates, and Testudines, where endemic species accounted for 39.43, 33.73, and 16.67% of total species, respectively, and exhibited much higher proportions of threatened taxa than non-island endemics (Fig. 3). Nearly all island endemic primates (96.51%) and the vast majority of island endemic turtles and tortoises (86.05%) were classified as threatened, while island endemic parrots showed over a threefold higher likelihood of extinction risk relative to continent endemic or dual-range species (table S5). Binomial tests further revealed pronounced clustering of extinction risk among particular families (figs. S1 to S4). Standout examples of families with significantly elevated threat proportions after Dunn-Šidák correction included Procellariidae (33 of 46 threatened) and Rallidae (42 of 63 threatened) among birds; Lemuridae (21 of 21 threatened) and Cheirogaleidae (35 of 38 threatened) among mammals; Eleutherodactylidae (138 of 163 threatened) and Rhacophoridae (93 of 165 threatened) among amphibians; and Testudinidae (24 of 24 threatened) and Diplodactylidae (41 of 55 threatened) among reptiles. Conversely, some families, such as Meliphagidae (9 of 119 threatened) and Muridae (70 of 206 threatened), exhibited significantly lower extinction risk than expected (all listed families with *P* < 0.001 after Dunn-Šidák correction; full results for all significantly higher- and lower-risk families are provided in data S5), highlighting the taxonomic selectivity of extinction crisis on islands.

**Fig. 4.**
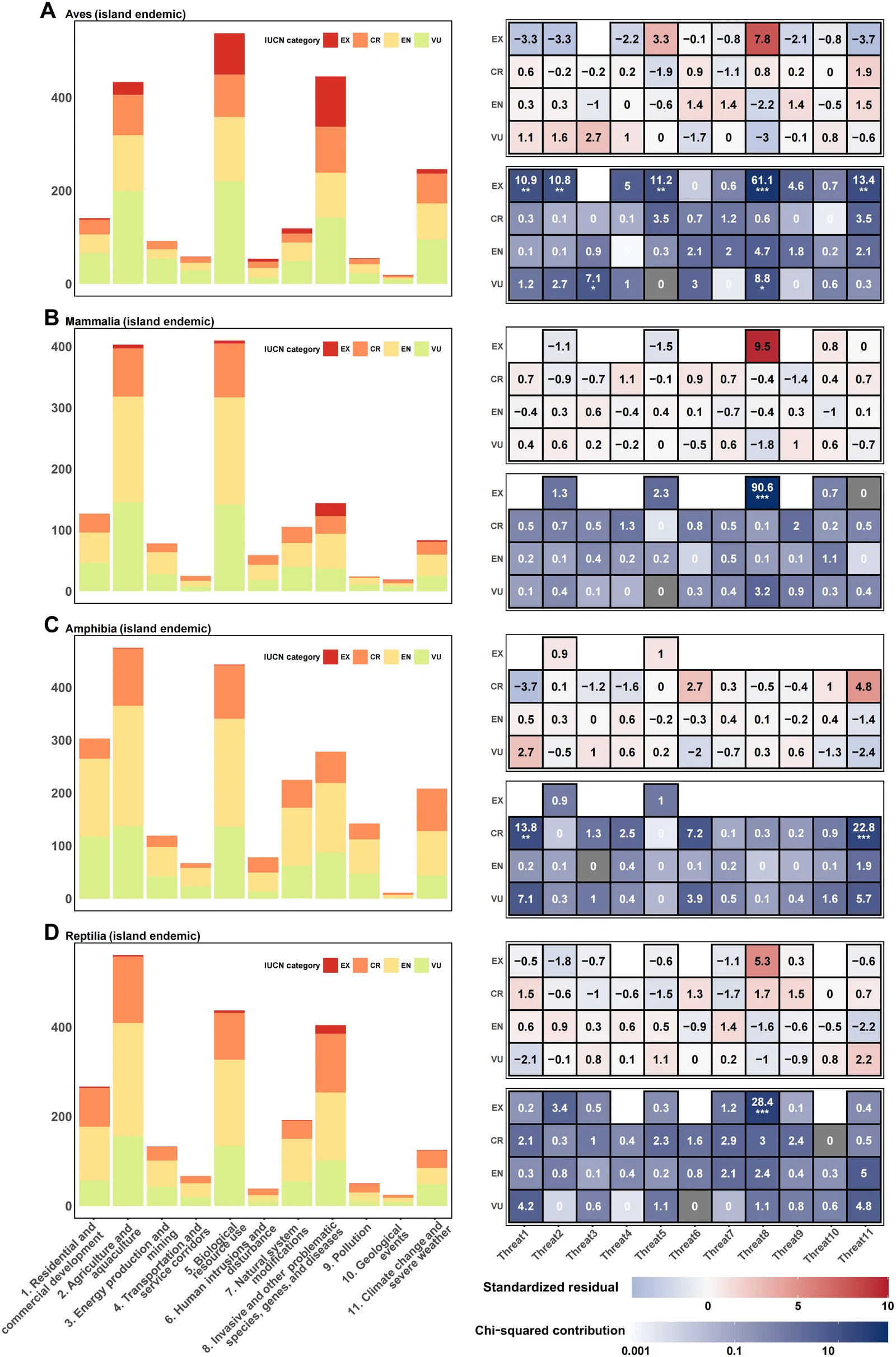
Association between threat types and IUCN Red List categories for island endemic terrestrial vertebrates. We compared threat types across IUCN Red List categories for island endemic terrestrial vertebrates: (**A**) Aves, (**B**) Mammalia, (**C**) Amphibia, and (**D**) Reptilia. For each class, the bar plots show the observed number of species per threat type, the heatmaps of standardized residuals highlight deviations from the expected counts under the chi-squared independence assumption, and the chi-squared contribution heatmaps show each cell’s contribution to the overall chi-squared statistic. Stars indicate false discovery rate–adjusted significance (*P < 0.05, **P < 0.01, and ***P < 0.001). Overall chi-square tests are significant for all classes, with all *P* < 0.001. Source data are provided in data S6.

Chi-square tests evaluating the association between IUCN threat categories and threat types indicate that, compared with continent endemics and dual-range species, island endemics display a stronger structured relationship between threat categories and types (data S6). For island endemics, significant deviations from expected threat distributions were detected in all four major classes (all *P* < 0.001). Continent endemics also exhibited significant threat structuring in amphibians and mammals (both *P* < 0.001). In contrast, dual-range species showed no significant association between threat categories and threat types in any taxonomic class (all *P* > 0.3). Island endemics are predominantly threatened by agriculture (threat 2), biological resource use (threat 5), and invasive species (threat 8) in terms of quantitative statistics (Fig. 4). Analysis across taxonomic groups further reveals pronounced differentiation: Invasive species are the primary driver of extinct and threatened bird, mammal, and reptile endemics (e.g., residual = 7.82 for EX birds), whereas climate change and severe weather (threat 11) seriously affect critically endangered amphibian endemics (residual = 4.77). Moreover, comparisons across threat categories indicate that past island extinctions were primarily associated with invasive species and overexploitation, and these pressures continue to exert profound impacts on extant species. Critically endangered island endemics are also simultaneously affected by emerging threats such as pollution and climate change (Fig. 4), indicating that the drivers of extinction risk have shifted over time. Continent endemics exhibited a restricted pattern of threat association, with invasive species (threat 8) overrepresented in the EX category of continental mammals (residual = 8.03; *P* < 0.001) and in both EX (residual = 2.98; *P* < 0.05) and CR (residual = 5.76; *P* < 0.001) categories of amphibians. By contrast, dual-range species exhibited no detectable linkage between threat types and Red List categories, suggesting a more diffuse distribution of threats (data S6).

### Biological, environmental, and anthropogenic drivers of extinction risk

**Fig. 5.**
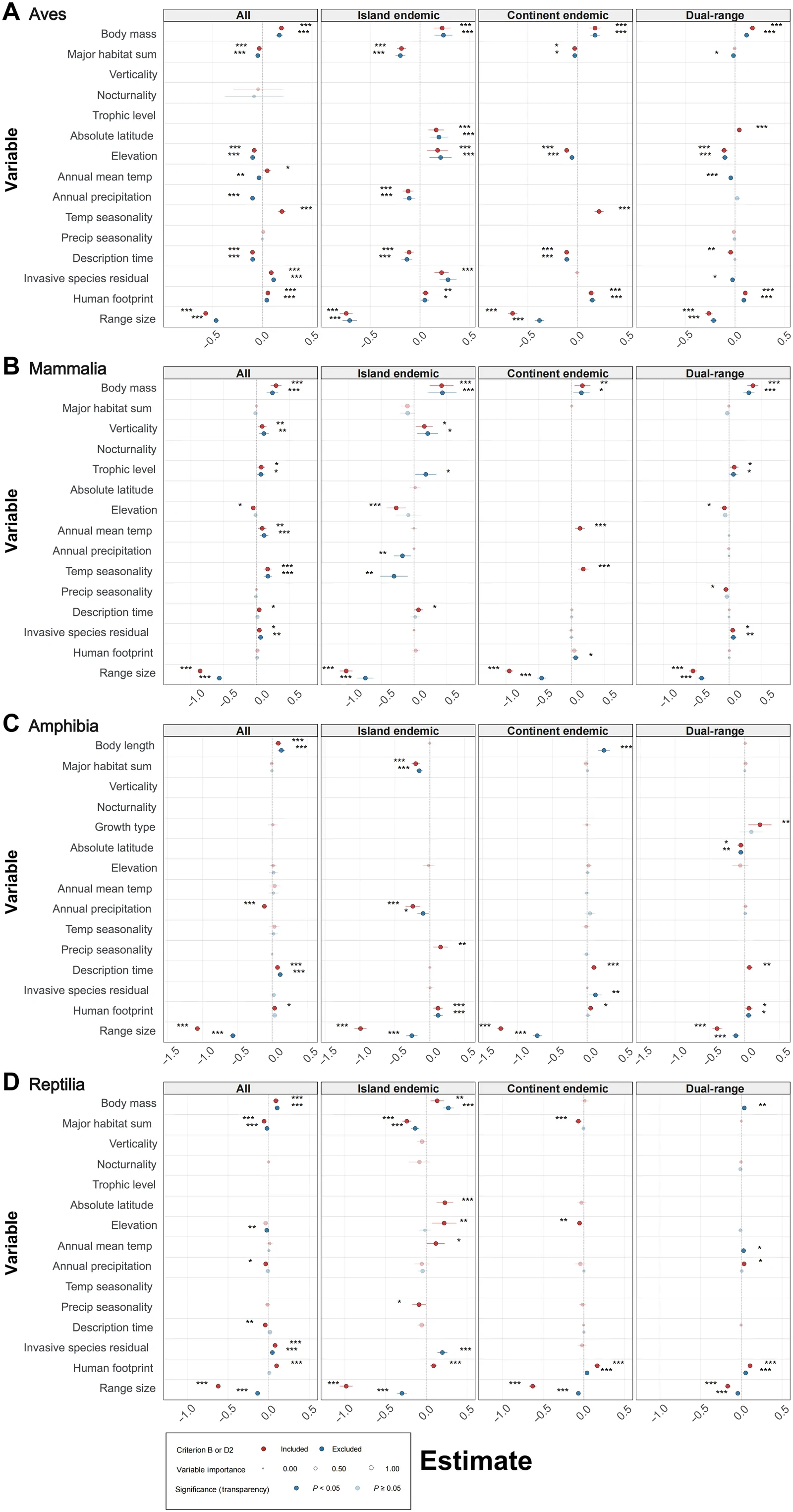
Model-averaged estimates from 100 paired datasets reveal key correlates of extinction risk across global terrestrial vertebrates. For ordinal extinction risk categories (0 to 5; from LC to EX), significant variables (*P* < 0.05; two sided) within the 95% confidence set are shown with model-averaged estimates derived from 100 paired datasets, incorporating both within-dataset and between-dataset variance. Panels (**A**) to (**D**) show results for (A) birds, (B) mammals, (C) amphibians, and (D) reptiles. Colors distinguish model types: red for models including all species; blue for models restricted to species not listed as threatened under criterion B or D2. Dot size represents variable importance, transparency indicates statistical significance, and asterisks denote significance levels (*P < 0.05, **P < 0.01, and ***P < 0.001). Horizontal bars indicate 95% confidence intervals. Variables not shown were not retained in the final model-averaged outputs, either because they did not receive sufficient support in univariate analyses or were not included in the best-supported models. Facets separate species by endemicity type: all species, island endemics, continent endemics, and dual-range species.

Across all vertebrate classes, geographic range size and habitat breadth emerged as the most robust predictors of extinction risk among island endemic species (Fig. 5 and data S8). Island endemics consistently showed a negative association between range size and extinction risk, a pattern remained even after excluding species threatened under IUCN criteria B (small geographic range) or D2 (area of occupancy <20 km^2^ or number of locations ≤5). A similar pattern was observed for habitat breadth, with species occupying fewer major habitat types generally exhibiting higher extinction risk in the subsets excluding species listed under IUCN criterion B or D2 (hereafter abbreviated as B- or D2-excluded subsets and B- or D2-listed species), although the effect was only marginally significant for mammals (model-averaged parameter estimates, hereafter abbreviated as estimate = −0.0964, *P* = 0.101).

Among island endemic species, body size represented the most influential predictor of extinction risk among ecological traits. Species with larger body sizes exhibited higher extinction risk in birds (estimate = 0.2363, *P* = 3.72 × 10^−7^), mammals (estimate = 0.4330, *P* = 7.79 × 10^−5^), and reptiles (estimate = 0.2705, *P* = 3.11 × 10^−15^) (Fig. 5). In contrast, for island endemic amphibians, no significant association was detected between body size and extinction risk in either the full dataset or the criterion B– or D2–excluded subset. This contrasts with continent endemic amphibians, for which body size became a significant positive predictor of extinction risk once criterion B– or D2–listed species were excluded (estimate = 0.2403, *P* = 4.54 × 10^−8^). Apart from body mass and habitat breadth, most ecological traits showed weak or inconsistent associations with extinction risk. Adult microhabitat height (estimate = 0.2803, *P* = 0.0107) and trophic level (estimate = 0.1790, *P* = 0.0303) were significant predictors only for island endemic mammals (Fig. 5). In contrast, other ecological attributes retained in the model-averaged results did not reach statistical significance. In particular, vertical stratum use and nocturnality appeared in the model-averaged set for island endemic reptiles in the parallel dataset including B- or D2-listed species, but their estimated effects were weak and nonsignificant.

For island endemics, univariate analyses indicated that extinction risk was negatively associated with the average elevation within the species’ distributional range in birds, mammals, and reptiles (fig. S5 and data S8). However, after multivariate model averaging and exclusion of 5794 species listed under IUCN criterion B or D2 that have very small geographic ranges or highly restricted area of occupancy, this relationship was retained only in island endemic birds, where it reversed in direction and became significantly positive (estimate = 0.2075, *P* = 2.67 × 10^−4^). Across criterion B– or D2–excluded island endemic groups, mean annual precipitation was consistently and significantly negatively associated with extinction risk in birds, mammals, and amphibians (estimate = −0.1077, *P* = 5.07 × 10^−4^; estimate = −0.1764, *P* = 0.00781; estimate = −0.095, *P* = 0.00234, respectively), whereas no such relationship was detected for reptiles (estimate = −0.0453, *P* = 0.145) (Fig. 5). Mean annual temperature showed no significant association with extinction risk in any island endemic group after exclusion of criterion B– or D2–listed species. However, when criterion B– or D2–listed species were retained, extinction risk in island endemic reptiles exhibited a positive association with mean annual temperature (estimate = 0.2284, *P* = 1.23 × 10^−5^), a pattern not observed in the other three vertebrate classes regardless of criterion B or D2 inclusion. On the basis of a combined assessment of the model-averaged results and the correlation structure among predictors, we found that the strongest divergence emerged between birds and mammals. Although latitude and temperature seasonality were strongly correlated with extinction risk in both island endemic birds (ρ = 0.81) and mammals (ρ = 0.90) (data S7), the two taxa exhibited opposite responses to these climatic gradients. Island endemic birds faced higher extinction risk in regions of higher latitude (estimate = 0.1912, *P* = 2.3 × 10^−5^), which typically correspond to stronger temperature seasonality, whereas island endemic mammals showed elevated extinction risk in regions with weaker temperature seasonality (estimate = −0.3053, *P* = 0.00429) that is generally associated with lower latitudes. In contrast, precipitation seasonality differentiated amphibians and reptiles only when criterion B– or D2–listed species were included. Specifically, precipitation seasonality increased with extinction risk in island endemic amphibians (estimate = 0.1557, *P* = 0.00356) but decreased with extinction risk in island endemic reptiles (estimate = −0.0894, *P* = 0.0335) (Fig. 5). No significant effect of precipitation seasonality was detected in birds or mammals nor in any taxonomic group after exclusion of criterion B– or D2–listed species.

For island endemics, human footprint in univariate analyses was generally positively associated with extinction risk (data S8). After excluding criterion B– or D2–listed species, however, human footprint remained positively associated with extinction risk only for amphibians (estimate = 0.1211, *P* = 4.06 × 10^−4^) and birds (estimate = 0.049, *P* = 0.0241). Invasion pressure was positively associated with extinction risk in island endemic birds and reptiles (estimate = 0.2827, *P* = 8.24 × 10^−11^; estimate = 0.1975, *P* = 6.5 × 10^−10^, respectively). Description time showed a negative correlation with extinction risk for island endemic birds (estimate = −0.1317, *P* = 1.78 × 10^−6^), whereas the effect was nonsignificant for island endemic mammals, reptiles, and amphibians (Fig. 5).

## DISCUSSION

Here, we assembled a global dataset of island endemic terrestrial vertebrates to assess their current extinction risk, identified spatial and taxonomic hotspots, and examined the ecological, environmental, and anthropogenic correlates of extinction vulnerability. This integrated framework reveals both common and island-specific pathways of biodiversity loss, offering crucial insights for predicting and mitigating future extinctions in these evolutionarily unique systems.

Although islands host less than one-quarter of all non-DD terrestrial vertebrate species, they account for the vast majority of known extinctions since 1500 CE (*10*, *20*). Among the 7569 island endemics analyzed, more than 3% have already gone extinct, and more than one-third are currently threatened, which is several times higher than the rate observed for continent endemic or dual-range species. Chi-square analyses confirmed that this disparity is highly significant across all vertebrate classes except amphibians, underscoring the disproportionate vulnerability of insular vertebrate faunas. Amphibians differ from other vertebrates in that their dominant extinction drivers, particularly chytrid fungal disease, pollution, and climate change, are globally pervasive and affect island and continental populations indiscriminately (*49*–*51*). In addition, both island and continental amphibians have already undergone severe historical declines and extinctions, resulting in strong extinction filtering. This process removes the most vulnerable lineages, leaving a surviving fauna with broadly similar sensitivities across both environments (*50*). Last, uneven survey effort and high proportions of DD species in amphibians may obscure true threat patterns, collectively diminishing the expected contrast in threat levels between island endemics and non-island endemics (*52*).

The loss of island vertebrates also has far-reaching ecological consequences because many insular ecosystems rely heavily on endemic species to perform key ecological functions (*53*, *54*). Large-bodied endemic vertebrates often act as primary seed dispersers, pollinators, or top consumers, and their disappearance can trigger cascading effects that restructure entire ecological communities. For example, the loss of frugivorous vertebrates on islands can disrupt seed dispersal networks, alter plant recruitment dynamics, and ultimately reshape vegetation composition (*47*, *48*, *55*). Similarly, declines of predators and large herbivores can propagate through food webs, leading to trophic imbalances and altered ecosystem processes (*56*). These cascading effects are a central feature of Anthropocene defaunation and draw attention to how species extinctions can erode ecosystem functioning well beyond the loss of individual taxa (*57*, *58*). Because island ecosystems typically contain fewer redundant species performing similar functions, the ecological impacts of endemic vertebrate extinctions are often amplified relative to continental systems (*59*). Consequently, preventing further losses of island endemic vertebrates is not only critical for preserving biodiversity but also for maintaining the ecological processes that sustain island ecosystems.

Our analyses show that island endemic vertebrates are unevenly distributed across archipelagos. This spatial mismatch raises the challenge of allocating limited conservation resources and effort most effectively (*60*). The long-standing SLOSS debate cautions against a priori favoring either a single large area or multiple small areas for conservation (*61*, *62*). Large archipelagos accumulated vast endemic richness and evolutionary history, so preserving intact habitat and connectivity there maintains both broad diversity and deep evolutionary lineages (*63*). At the same time, small or modest-richness islands that now host disproportionately high proportions of threatened taxa demand targeted measures including invasive species eradication, strict biosecurity, habitat restoration, and species-level interventions (*64*). Conservation interventions and protected area design need to balance broad-scale diversity preservation with targeted interventions, ensuring that conservation efforts are both effective and spatially strategic (*65*).

Island endemic vertebrates in several major clades face exceptionally high extinction risk, particularly parrots (Psittaciformes), primates (Primates), and tortoises (Testudines). We advocate prioritizing conservation for these flagship clades, which exhibit trait combinations, such as relatively large body size, slow life-history strategies, narrow habitat breadth, and specialized trophic roles, which are distinct from their continental relatives and are highly vulnerable in the Anthropocene (*39*, *66*, *67*). These intrinsic traits, coupled with extrinsic pressures such as habitat loss, hunting, and wildlife trade, amplify their extinction risk on islands (*67*–*69*). For example, a disproportionate number of 16 extinct parrot species were endemic to islands and single countries and were typically characterized by large body size and strong habitat specialization (*69*). Protecting them not only reduces their extinction risk directly but also helps maintain key ecological functions (e.g., seed dispersal, vegetation dynamics, and ecosystem engineering provided by large tortoises) (*11*, *68*) and benefits co-occurring island species through habitat and ecosystem safeguarding indirectly (*70*–*72*). In addition, within some taxonomic families, the island endemic subset consists of only a few members that are nonetheless almost entirely threatened (e.g., Loridae, Trionychidae, etc.) or already extinct at the species level (e.g., Mohoidae, Nesophontidae, etc.). They represent a unique evolutionary history and emphasize the need to prevent similar losses in related or ecologically comparable lineages (*73*).

Island endemic vertebrates exhibit an uneven pattern across extinction-risk categories and threat types, with clear distinctions between extinct and extant critically endangered species, as well as among threat categories and taxonomic groups. Whereas previous studies based on threatened proportions identified habitat alteration as the most important threat for all four classes, our residual-based analysis indicates a distinctive threat structure in island endemic vertebrates (*26*). Historical extinctions were primarily driven by invasive species and overexploitation (*20*), whereas many currently critically endangered species face a combination of these legacy threats and emerging pressures such as pollution and climate change, particularly in amphibians (*50*, *74*). Species at high extinction risk are often exposed to multiple concurrent threats, and interactions among threat categories may further shape vulnerability patterns (*26*, *75*, *76*), suggesting that future work should explore the co-occurrence and potential linkage of threats. However, the completeness of IUCN threat assessments varies across taxa and regions, meaning that some threats may be underrepresented, potentially biasing observed patterns (*77*). Moreover, even when threat categories are assigned, key information about the timing of impacts and the severity or scope of those impacts is frequently recorded as unknown, further limiting the reliability of cross-taxon comparisons. Recent predictive-model studies suggest that species currently classified as DD may, in fact, face higher extinction risk than data-sufficient species (*78*). Conservation strategies should therefore consider both the differences among extinction risk categories and the limitations of existing data, prioritizing islands and species where threats are known to be severe while acknowledging assessment gaps (*79*).

Our global analysis reveals that extinction risk in island endemics is distributed and driven by the concerted action of biological traits, environment condition, and anthropogenic pressures. The most consistent predictor across all taxa was small range size, a pattern that remained robust in analyses excluding species listed under IUCN criterion B or D2. While this pattern between range size and extinction risk does not directly test the species-area predictions of classical island biogeography theory (*80*), it aligns with the premise that range-restricted species face inherent vulnerability exacerbated by the insular context (*81*). Complementing this pattern, for island endemics, species occupying fewer macrohabitat types demonstrated consistently higher extinction risk, a relationship that was retained in the model-averaged results regardless of whether species assessed under IUCN criterion B or D2 were included or excluded, although its relative importance was lower in mammals. This consistency suggests that narrow habitat specialization constitutes an independent axis of vulnerability beyond range size alone. Beyond these two universal drivers, however, our results reveal a complex interplay of additional factors whose effects varied among taxonomic groups and between parallel analyses. By explicitly evaluating models with and without species assessed under IUCN criterion B or D2, our approach not only reduces potential circularity associated with including range size as a predictor in the extinction risk model to some extent but also explains that species classified under different threat criteria may exhibit distinct vulnerability profiles (*82*). This pattern is consistent with emerging evidence that range-restricted and widespread species often face different extinction processes and therefore may not conform to a single set of conservation priorities (*83*).

The relationships between biological traits and extinction risk revealed both commonalities and notable differences among taxa. The consistent vulnerability of larger-bodied species in mammals, birds, and reptiles aligns with global patterns of threat in *K*-selected species (*84*). This pattern likely reflects multiple mechanisms: Larger species typically exhibit lower mass-specific metabolic rates, reduced reproductive rates, larger area requirements, and are often disproportionately targeted by human hunting (*30*). In contrast, the lack of a strong body size effect in amphibians also carries important ecological implications (*85*). This pattern may be attributed to their unique physiological constraints, such as permeable skin and complex life cycles that heighten sensitivity to environmental change. In addition, both the largest and smallest amphibian species face elevated threats: Larger species are often targeted by hunting and collection, while smaller species may be more susceptible to microhabitat loss and disease (*27*). The combined pressures, including habitat degradation, emerging diseases, and climate change, likely overwhelm any signal of size-dependent vulnerability. Similarly, the variable importance of functional traits reveals distinct ecological mechanisms underlying taxon-specific vulnerabilities. For mammals, both higher trophic position and arboreality emerged as significant predictors of extinction risk. This pattern highlights the particular vulnerability of forest-dependent species occupying higher trophic levels such as Madagascar’s lemurs (Lemuridae), which face extreme extinction risk due to widespread habitat loss from deforestation, natural disasters and wild fire, hunting pressure, and their ecological specialization (*24*, *51*). Several traits, including activity patterns and vertical habitat use, did not emerge as significant predictors of extinction risk across all taxa in our global models. However, their absence in such macroscale analyses does not negate their potential importance for specific lineages or at finer spatial resolutions, where local ecological contexts may render these factors critical determinants of extinction risk. In the Pacific Ocean (e.g., Norfolk Island and Minami-Daitō Island), arboreal nesting songbirds experience high nest failure rates when their nests are structurally connected to surrounding vegetation, allowing invasive ship rats (*Rattus rattus*) to access them regardless of nest height, while habitat generalists with broader vertical occupancy show markedly higher nest survival (*86*, *87*). Likewise, nocturnal or crepuscular island lizards often evade diurnal predators (*88*, *89*). Comparative evidence from large-scale trait-based studies provides additional context for our island focused results. These analyses identify several functional groups with consistently elevated extinction risk across terrestrial vertebrates, including fossorial amphibians, scavenging birds, and terrestrial pedal squamates (*76*, *90*). Generation length, dispersal ability, and several other life-history traits were not included in our global models because of limited data availability. Previous study, however, suggests that these traits may also represent important predictors of vulnerability (*91*). While these analyses were not developed specifically for island endemics, they provide a useful comparative context and may help reveal functionally vulnerable groups relevant for island conservation planning.

The observed geographic and climatic patterns in extinction risk likely reflect the ecological sensitivities and habitat dependencies of island endemic vertebrates. Among the climatic variables examined, precipitation emerged as the most consistent predictor across multiple vertebrate classes. Higher mean annual precipitation was associated with lower extinction risk in birds, mammals, and amphibians, suggesting that wetter island environments may provide more stable primary productivity, freshwater availability, and habitat structure, all of which can buffer populations against demographic fluctuations and environmental stress (*38*). In contrast, mean annual temperature showed no consistent association with extinction risk once range-restricted species listed under criterion B or D2 were excluded, indicating that thermal conditions alone may not be the primary limiting factor for most island endemic vertebrates. The divergent responses of birds and mammals along latitudinal and temperature seasonality gradients illustrate taxon-specific sensitivities to climatic variability. Island endemic birds tend to exhibit lower flight capacity, which limits their ability to evade seasonal or episodic environmental stressors (*92*). High-latitude island populations face additional challenges, including short breeding seasons and limited overwintering refugia, which further intensify their extinction vulnerability. In contrast, island mammals on low-latitude tropical islands face an elevated extinction risk, largely due to stronger and earlier impacts of humans. Tropical islands were typically colonized sooner and developed more intensively, bringing with them invasive mammals such as rats, pigs, and dogs, as well as extensive forest clearing, hunting, and agricultural expansion (*20*, *93*). Amphibians are highly dependent on stable humidity, so even small changes in hydrology, soil moisture, or canopy structure can disproportionately affect survival and reproduction (*94*). For reptiles, the differences generated by including or excluding criterion B– or D2–listed species arise from both ecological and statistical mechanisms (*95*). Many range-restricted reptiles occupy extremely narrow climatic niches, and their inclusion amplifies apparent sensitivity to multiple climatic gradients. After removing these highly specialized taxa, we observe that for broader-ranging but still threatened island reptiles, no single environmental variable emerges as a dominant limiting factor for persistence. Overall, climate does not act as a uniform driver of extinction for island-endemic species but rather shapes extinction risk by amplifying existing ecological constraints, limited ranges, and life-history traits. As climate change progresses, these context-dependent effects are likely to intensify, emphasizing the need to identify and protect climatically stable refuges to buffer vulnerable island species from increasing environmental stress (*7*).

Anthropogenic pressures showed distinct pathways across taxa. Residuals from the relationship between invasive pressure and range size represent deviations from expected values. Island endemic birds and reptiles with positive residuals are more likely to be threatened or extinct. This pattern may arise because many island birds and reptiles forage on or near the ground, bringing them into frequent contact with invasive mammalian predators, while their body sizes often fall within the preferred prey range of these invaders (*43*, *96*). Furthermore, their naïveté toward previously unencountered predators further increases vulnerability (*96*). The lack of significance in some models does not mean invasion threats are overstated. Instead, it suggests that the aggregate invasion richness may mask the outsized effects of a few high-impact predators such as rats, cats, and snakes, whose ecological impacts exceed what coarse richness metrics can capture (*97*, *98*). On the basis of reducing overall invasion intensity through strengthened biosecurity, pathway management, and broad invasive control, conservation efforts should further prioritize preventing and eradicating key invasive predators (*99*). Birds are the best studied vertebrates, yet patterns related to species description indicate that many extinctions driven by human activities remain unfound, especially among insular species and those with restricted ranges (*31*). Conservation action should prioritize the discovery and formal description of new species, assess their extinction risk, and implement targeted protection of critical habitats to prevent dark extinction and undetected biodiversity loss (*100*).

Human footprint presented a broad yet complex signal. In univariate analyses, it was generally positively correlated with extinction risk across taxa. However, its influence tended to diminish after model averaging, partly because the human footprint index integrates multiple factors such as infrastructure, land use, and artificial light, which can dilute the effects of its most direct components. In addition, model-averaging distributes weight across numerous candidate models, so predictors that co-occur with other strong variables may receive lower averaged coefficients and importance scores. Island endemic birds and amphibians experience higher extinction risk in areas of intense human footprint, collectively illustrating that taxa at opposite ends of the dispersal spectrum are particularly vulnerable to human disturbance. Future research should focus on species with extreme dispersal abilities and range sizes, a pattern recently corroborated for reptiles by studies on wildlife trade (*83*).

Our global-scale study demonstrates that conservation strategies for island endemic species cannot be directly extrapolated from those developed for continental or dual-range taxa. In some cases, island and nonisland species exhibit opposite patterns in key drivers of extinction risk. For example, bird extinction risk on islands is negatively correlated with elevation, whereas for continental and dual-range birds, the relationship is positive. In addition, several factors are not significant predictors for continent endemic or dual-range species but show unique effects on island endemics, such as the importance of vertical habitat use in island endemic mammals. Future island development and management must avoid the uncritical application of strategies derived from continental experience or general rules (*42*).

Planners should adopt risk assessments tailored to islands, prioritize the maintenance of climatic and habitat diversity, and implement proactive measures such as targeted invasive species control, reducing habitat fragmentation and placing infrastructure to minimize exposure to extreme climatic events. Future research should also aim to overcome the current imbalance in knowledge, where island endemic species are both more threatened and less studied than their continental or dual-range counterparts (*95*). Given that more than half of island endemic vertebrates with known population trends are currently declining and that declining populations overwhelmingly dominate among threatened taxa (>70% across most classes), expanding long-term population monitoring for island endemic species should be a priority for future research. This monitoring will be essential for tracking demographic changes through time, distinguishing short-term fluctuations from sustained declines, and enabling timely conservation interventions before extinction becomes unavoidable (*101*). By providing early warning of irreversible population declines, these efforts can help prevent the delayed consequences of “extinction debt,” whereby species may continue to decline and eventually disappear even after the primary drivers of decline have been reduced (*102*).

We acknowledge several limitations in our study. First, data coverage varies among taxonomic groups, which may introduce biases in the assessment of extinction risk (*103*). Species trait data and expert-based distribution records may contain inaccuracies, which can potentially affect trait-risk correlations and spatial patterns (*104*). Moreover, the use of global databases may overlook locally adapted populations or finer-scale ecological variation. Certain remote or understudied islands remain underrepresented, potentially skewing species richness and threat patterns (*105*). In addition, DD species were excluded from our study. These species are often rare, have restricted ranges, and occur in poorly surveyed regions, meaning their omission could underestimate extinction risk and bias trait-risk and spatial analyses (*78*). Similarly, a subset of species was excluded from trait-based analyses due to insufficient ecological and life-history data required for reliable imputation and modeling. Although necessary for statistical robustness, this filtering may also disproportionately remove poorly studied species, which could themselves be vulnerable to extinction. Together, our results likely provide a conservative estimate of extinction risk for island endemics. Finally, although we identify correlations between intrinsic and extrinsic factors and extinction risk, the causal relationships underlying these patterns require further investigation through targeted mechanistic studies and long-term monitoring.

In summary, our study provides the first comprehensive global analysis of extinction risk in island endemic terrestrial vertebrates, integrating spatial, taxonomic, ecological, and anthropogenic perspectives. We demonstrate that islands harbor a disproportionate share of biodiversity and extinction risk, with threats and extinction vulnerability structured phylogenetically and taxon specifically. Intrinsic biological traits, such as small geographic range and habitat specialization, interact with external anthropogenic pressures, including invasive species, pollution, and overexploitation, to shape extinction risk of island endemic terrestrial vertebrates. These findings highlight the need for nuanced, clade-specific conservation strategies and emphasize the importance of protecting key biodiversity areas. Our framework offers a quantitative and actionable blueprint for prioritizing interventions to prevent the disproportionate loss of evolutionary distinct lineages on islands.

## MATERIALS AND METHODS

### Species list assembly and classification

We compiled a comprehensive global list of terrestrial vertebrate species from the IUCN Red List of Threatened Species, version 2024-1 (*21*). Using the advanced search function, we retrieved summary information for 35,498 species across four major vertebrate classes: Aves (*n* = 11,195), Mammalia (*n* = 5983), Amphibia (*n* = 8011), and Reptilia (*n* = 10,309). The downloaded data included each species’ scientific name, taxonomic classification, IUCN Red List category, population trend, assessment criteria, and authority and year of original description.

Taxa classified as Data Deficient (DD) were excluded following previous studies (*27*, *37*), resulting in a preliminary dataset of 32,214 species. We treat species listed as “Extinct” and “Extinct in the Wild” as extinct (*39*). To ensure taxonomic consistency and avoid duplication, we performed comprehensive taxonomic reconciliation and name standardization. Detailed data processing and cleaning steps are provided in Supplementary Methods and data S1.

To focus on terrestrial vertebrates, we used the IUCN classification of ecosystem types (terrestrial, freshwater, and marine). Species occurring exclusively in marine ecosystems were thus excluded, while those occupying terrestrial or freshwater environments were retained. After this filtering, the final dataset contained 31,865 terrestrial vertebrate species (data S2). Habitat data were programmatically retrieved using the rl_search function from rredlist (v0.7.1) (*106*).

Island endemic species are those whose geographic distributions are entirely restricted to oceanic islands smaller than Australia (*10*). To determine species endemicity, we classified each species according to whether its range polygon intersected exclusively with continental landmasses, exclusively with islands, or with both. Continental landmasses were derived from the land polygon layer in Natural Earth v.5.1.1 (*107*), while island polygons were sourced from v.4.1.0 for minor islands (<2 km^2^) and v.5.1.1 for major islands, respectively.

### Analyses of global hotspots of endemic species diversity and extinction risk

To identify global hotspots of island endemic diversity and extinction risk, we analyzed the spatial distribution of endemic species in relation to archipelagos delineated by Weigelt *et al.* (*108*). The geographic centroid of each species’ range was derived from expert-curated distribution maps (*21*, *109*, *110*), supplemented by additional procedures (see Supplementary Methods). A nearest-neighbor analysis was then performed in ArcGIS Pro v.3.2 to assign each species to the archipelago containing its primary area of occurrence. The number of species per archipelago were summarized to characterize global hotspots of endemic species diversity and extinction risk. The use of distribution centroids to summarize species locations has been validated in macroecological research for linking abundance patterns with range characteristics and is applicable in large-scale cross-taxonomic analyses (*111*, *112*).

To ensure robust comparison of island endemism while accounting for differences in species richness among taxonomic orders across vertebrate classes, we calculated the proportion of island endemics and ranked orders within each class by their degree of island endemism, restricting the analysis to orders containing at least 20 species. This revealed which orders in birds, mammals, reptiles, and amphibians harbor the highest numbers of island endemic species. For these orders, we summarized the extinction risk both for all species and specifically for island endemics, providing a coarse overview of which lineages contribute disproportionately to global island biodiversity and extinction risk.

To examine the phylogenetic distribution of extinction risk among island endemics, we compared the proportion of species with high extinction risk (EX, CR, EN, or VU) within each family to the overall class-level proportion. Exact binomial tests were used to evaluate whether the observed number of threatened species in a family differed significantly from the expected value under a random distribution, and this analysis was restricted to families containing at least five island endemic species to ensure sufficient statistical power and to avoid unstable estimates driven by very small sample sizes (*113*). For each vertebrate class, *P* values and confidence intervals were derived using the same exact binomial framework with Clopper-Pearson intervals. Dunn-Šidák corrections were applied to adjust for multiple comparisons because each family was tested independently. This approach ensures methodological consistency and provides reliable identification of families whose extinction risk deviates significantly from the class-level baseline (*113*–*115*).

To assess the contribution of different threat types to the extinction risk of terrestrial vertebrates, contingency tables were constructed between threatened status and IUCN threat categories within three endemism groups: island endemics, continent endemics, and species distributed on both islands and continents. Chi-square tests were then used to evaluate the association between threat categories and threatened status. Standardized residuals were subsequently examined to identify threat types that were significantly overrepresented or underrepresented within particular threatened categories. This analytical framework allows a quantitative assessment of the systematic influence of different threat factors on the extinction risk of terrestrial vertebrates, thereby providing a robust basis for understanding the drivers of extinction in island species (*22*).

### Assembly of species-level predictor variables

We assembled a comprehensive dataset of species-level variables for non-DD terrestrial vertebrates to investigate factors associated with extinction risk. The variables assembled encompassed three broad domains: (i) ecological and life-history traits, (ii) geographic and environmental characteristics, and (iii) anthropogenic and historical pressures. Ecological and life-history traits—including body size, activity pattern, vertical habitat use, trophic level, habitat breadth, and amphibian developmental mode—were primarily sourced from the TetrapodTraits database (*116*) and complemented with multiple published trait datasets to maximize data completeness across vertebrate classes (*117*–*122*). We selected these traits because they represent functional dimensions that are comparable and ecologically meaningful across classes, have been empirically linked to extinction risk or vulnerability in island endemic species, and are supported by adequate data availability for robust cross-class analysis (*24*, *27*).

Body size was represented by body mass for birds, mammals, and reptiles, and by body length for amphibians. Body mass is a standardized metric widely used in comparative ecological analyses and is strongly associated with metabolic and life-history constraints in birds, mammals, and reptiles (*84*). In amphibians, however, body length is more consistently reported across trait databases and therefore provides a more complete and comparable measure of body size for this group (*85*). Specifically for amphibians, developmental mode was used as a functional trait proxy in place of trophic level because amphibians undergo substantial dietary shifts between larval and adult stages, making direct comparisons of trophic level across their life-history challenging. To maintain consistency in the number of ecological traits included in our models for each vertebrate class, developmental mode was incorporated into the model. This trait has been shown to capture key aspects of ecological function in amphibians and has empirical links to extinction vulnerability, serving as a comparable functional dimension alongside trophic level in other vertebrate groups (*50*).

Geographic and environmental predictors included geographic range size derived from expert-curated and literature-informed distribution polygons, mean elevation across each species’ range, mean absolute latitude of the range centroid, and a set of bioclimatic variables summarizing annual means and seasonality of temperature and precipitation. To avoid potential circularity arising from the fact that range size is explicitly incorporated into IUCN threat assessments, we accounted for this issue in subsequent analyses by performing parallel models (see “Correlates of extinction risk”). These variables were originally sourced from the TetrapodTraits framework of Moura *et al.* (*116*), which is based on standardized global datasets such as EarthEnv (*123*) and CHELSA (*124*), and were further refined and supplemented in this study to correct inconsistencies arising from differences in taxonomic standards. Full details of data preprocessing and recalculation procedures are provided in Supplementary Methods.

Human-related pressures were characterized using human footprint, which provides temporally resolved estimates of anthropogenic land-use intensity (*125*, *126*). Species with larger geographic ranges naturally overlap with more invasive species, which leads to inflated raw invasion metrics. We therefore calculated relative invasion pressure as the residuals from a log1p-transformed regression of invasive species richness against range size (*64*, *127*, *128*). This approach reduces the effect of collinearity and isolates disproportionate invasion exposure (*129*). To control for potential biases in data availability and research effort, we included the time since formal species description as a predictor variable in our models. Long-known taxa may have benefited from greater conservation attention but have also been exposed to threats for longer durations (*130*), while newly described species frequently inhabit remote or already degraded regions, where threatening processes are often poorly quantified (*131*).

Continuous traits were recorded as numeric values and categorical traits as ordered factors. For instance, activity pattern was coded as 0 (nocturnal), 1 (diurnal), and 2 (cathemeral/crepuscular) to capture increasing behavioral flexibility following Davidson *et al.* (*132*). Full details of trait coding and ordinal ranking, geographic range construction and environmental extraction procedures, anthropogenic pressure calculations, and the derivation of description age are provided in Supplementary Methods and table S1.

### Data filtering and imputation

We restricted analyses to species having sufficient nonmissing trait data to ensure reliable imputation and modeling. Specifically, only species with values for two or more of the focal ecological and life-history traits were retained, except for a few classified as EX, which were retained to maintain representation of extinct taxa. Given their small number (*n* = 3), their inclusion is unlikely to affect the results. After this filtering, 30,711 species with sufficiently complete trait information were retained as the analytical subset for subsequent imputation and comparative modeling analyses, whereas 1154 species (≈3.6% of the initial dataset) were excluded because of insufficient trait data. Although cryptic species that were poorly studied may still face equal or greater extinction risk, the small proportion excluded is unlikely to substantially alter the inferred drivers of extinction risk and instead produces conservative estimates while also reducing the potential for large-scale bias in trait imputation and subsequent analytical inferences.

To address missing trait values while preserving biological structure, we used multiple imputation by chained equations (MICE) to estimate incomplete ecological and life-history variables (body size, activity pattern, trophic level, habitat breadth, and vertical habitat use), following established practices in macroecological analyses (*133*, *134*). MICE is advantageous for heterogeneous trait data, as it models each variable conditionally based on the others, can incorporate auxiliary information, and produces multiple plausible imputations that reflect uncertainty (*135*). Within our analytical framework, taxonomic information at the genus level was included as an auxiliary variable to enhance predictive accuracy and to reflect trait similarities constrained by phylogeny while avoiding the autocorrelation that could result from reusing phylogenetic information (*136*). Genera containing fewer than three species were grouped into an “other” category and treated as completely random during imputation to prevent poorly represented genera from exerting disproportionate influence. For genera with three or more species, trait values were imputed by referencing congeneric species. Details of data completeness before imputation and data sources are provided in data S3.

Multiple imputation approach was applied only to ecological and life-history traits, whereas all geographic, climatic, and anthropogenic variables were derived directly from global raster and polygon datasets without imputation. A total of 100 imputed datasets were generated, with each dataset produced after 100 iterations of chained equations to ensure convergence and stability. The imputations were performed in R using the mice package (*135*) under a fully conditional specification framework. We assumed that the missing data followed a missing-at-random mechanism, meaning that the probability of missingness depended only on observed variables (*133*). Predictive mean matching was applied to continuous variables and polytomous logistic regression to categorical variables. Model convergence was satisfactory, with no numerical instabilities or convergence problems reported during the imputation iterations. Rather than pooling the imputed values, each completed dataset was subsequently paired with one of 100 phylogenetic trees in the regression analyses (*137*), and results were synthesized later.

Phylogenetic relationships for each major vertebrate clade were obtained from published, time-calibrated supertrees (*74*, *138*–*140*). We verified the correspondence between binomial species names in the phylogenetic trees and our compiled species list. Synonyms and historical names were retained when referring to the same taxon. Species present in the phylogeny but absent from our list were removed. Species present in our list but missing from the phylogeny were appended using the add.species.to.genus function in phytools (*141*), which places missing taxa within their respective genera while maintaining the overall branch length structure. When insufficient congeneric representation prevented confident genus-level placement, species were randomly inserted within the corresponding clade to preserve topological balance. This reconstruction process was repeated 100 times for each clade, resulting in a set of 100 phylogenetic trees per group (*137*). These replicate trees were used in parallel with the 100 imputed datasets in subsequent models, thereby integrating both phylogenetic and data-imputation uncertainty.

### Correlates of extinction risk

To determine the correlates of extinction risk while accounting for shared evolutionary history, we fitted phylogenetic linear models (PLMs) using the phylolm() function in the phylolm R package (*142*), assuming a Brownian motion model of trait evolution. Before modeling, skewed predictor variables including body size, geographic range size, and description time were log transformed, whereas range centroid latitude was changed to absolute value. All continuous predictors were subsequently *z* standardized (mean = 0, SD = 1) when required (*143*). Full transformation procedures are detailed in table S1.

Analyses were performed separately for (i) all terrestrial vertebrates combined and (ii) three subsets based on species’ range types: island endemics, continent endemics, and dual-range species. Geographic range size is explicitly incorporated into the IUCN assessment framework, particularly under criterion B (small geographic range) and criterion D2 (area of occupancy <20 km^2^ or number of locations ≤5) (*144*). As a result, including range size as a predictor in correlative analyses may introduce circularity. In our dataset, 5794 of 30,711 assessed terrestrial vertebrate species were classified as threatened under criterion B or D2. To evaluate whether this potential circularity influenced our results, we repeated the full modeling workflow using two parallel datasets for each vertebrate class: one including all species and another excluding species assessed as threatened under criterion B or D2 (*145*).

The full analytical procedure consisted of four main steps. First, univariate PLMs were built to evaluate the impact of each variable on extinction risk, expressed as an ordinal IUCN threat score [Least Concern (LC) = 0 to EX = 5]. Variables with a marginal significance (*P* < 0.1) were retained for multivariate modeling following established recommendations to avoid prematurely excluding potentially important predictors during variable screening (*146*). Second, to reduce multicollinearity among predictors, pairwise Spearman rank correlation coefficients were calculated. When strong correlations were detected (|ρ| > 0.7), only one variable from each correlated pair with higher adjusted coefficient of determination (*R*^2^) estimated from univariate models was retained (*147*). Because correlation matrices were estimated separately across 100 paired datasets, Spearman correlation coefficients were transformed using Fisher’s *z* transformation before aggregation and subsequently back transformed for interpretation (*148*). This nonparametric approach was chosen because of its robustness to discrete ordinal variables and its ability to avoid assumptions of linearity or normality (*149*). The full set of correlation matrices for all classes and species subgroups is provided in data S7. Third, we constructed multivariate PLMs using all possible combinations of the retained variables. We used the Akaike information criterion (AIC) for model selection and evaluation. Competing models were ranked by AIC values, and models with ΔAIC ≤2 were considered statistically equivalent (*150*). Last, model averaging was applied across this top model set to estimate averaged coefficients, relative variable importance, and unconditional SEs (*151*).

To propagate both imputation and phylogenetic uncertainty, each of the 100 imputed datasets was paired with a randomly sampled phylogenetic tree, resulting in 100 PLMs realizations for each taxonomic class and endemicity subset. Model estimates were then synthesized using Rubin’s rule (*152*), which adjusts SEs and provides more accurate parameter estimates by incorporating both within-dataset and between-dataset variance. All analyses were conducted in R version 4.4.1 using the phylolm (*142*) and MuMIn (*153*) packages. Model-averaged estimates for each vertebrate group, endemicity category, and model type were summarized in data S9. As an additional robustness check, we also performed phylogenetic logistic regression using the phyloglm() function in the phylolm package, coding species classified as threatened (VU, EN, CR, and EX) as 1 and nonthreatened species (LC and NT) as 0 (*142*). The phyloglm results were broadly consistent with those obtained from the phylolm models in terms of effect directions and overall significance patterns. However, because phylogenetic logistic regression can only accommodate binary response variables (*154*), it inevitably removes the ordinal gradient of extinction risk among categories. By contrast, PLMs implemented in phylolm allow the ordered variation across IUCN threat categories to be retained. Therefore, we report the phylolm results as the primary analyses in the main text, while the phyloglm results are provided in the Supplementary Materials (data S10 and S11).

## Supplementary Materials

### The PDF file includes:

Supplementary Methods Legends for data S1 to S11 Tables S1 to S6 Figs. S1 to S5 References

## References

[1] A. D. Barnosky, N. Matzke, S. Tomiya, G. O. U. Wogan, B. Swartz, T. B. Quental, C. Marshall, J. L. McGuire, E. L. Lindsey, K. C. Maguire, B. Mersey, E. A. Ferrer, Has the Earth’s sixth mass extinction already arrived? Nature 471, 51–57 (2011). 21368823 10.1038/nature09678

[2] R. H. Cowie, P. Bouchet, B. Fontaine, The sixth mass extinction: Fact, fiction or speculation? Biol. Rev. 97, 640–663 (2022). 35014169 10.1111/brv.12816 PMC9786292

[3] J. E. M. Watson, N. Dudley, D. B. Segan, M. Hockings, The performance and potential of protected areas. Nature 515, 67–73 (2014). 25373676 10.1038/nature13947

[4] S. L. Pimm, C. N. Jenkins, R. Abell, T. M. Brooks, J. L. Gittleman, L. N. Joppa, P. H. Raven, C. M. Roberts, J. O. Sexton, The biodiversity of species and their rates of extinction, distribution, and protection. Science 344, 1246752 (2014). 24876501 10.1126/science.1246752

[5] G. Ceballos, P. R. Ehrlich, A. D. Barnosky, A. Garcia, R. M. Pringle, T. M. Palmer, Accelerated modern human-induced species losses: Entering the sixth mass extinction. Sci. Adv. 1, e1400253 (2015). 26601195 10.1126/sciadv.1400253 PMC4640606

[6] J. C. Garcia-R, M. Cimatti, M. Di Marco, Past and recent drivers of extinction risk in endemic New Zealand birds. Anim. Conserv. 28, 436–444 (2025).

[7] M. C. Urban, Climate change extinctions. Science 386, 1123–1128 (2024). 39636977 10.1126/science.adp4461

[8] N. Myers, R. A. Mittermeier, C. G. Mittermeier, G. A. B. Da Fonseca, J. Kent, Biodiversity hotspots for conservation priorities. Nature 403, 853–858 (2000). 10706275 10.1038/35002501

[9] A. Auber, C. Waldock, A. Maire, E. Goberville, C. Albouy, A. C. Algar, M. McLean, A. Brind’Amour, A. L. Green, M. Tupper, A functional vulnerability framework for biodiversity conservation. Nat. Commun. 13, 4774 (2022). 36050297 10.1038/s41467-022-32331-y PMC9437092

[10] J. M. Fernández-Palacios, H. Kreft, S. D. H. Irl, S. Norder, C. Ah-Peng, P. A. V. Borges, K. C. Burns, L. De Nascimento, J.-Y. Meyer, E. Montes, D. R. Drake, Scientists’ warning–The outstanding biodiversity of islands is in peril. Glob. Ecol. Conserv. 31, e01847 (2021). 34761079 10.1016/j.gecco.2021.e01847 PMC8556160

[11] S. Llorente-Culebras, C. P. Carmona, W. D. Carvalho, A. Menegotto, R. Molina-Venegas, R. J. Ladle, A. M. C. Santos, Island biodiversity in peril: Anticipating a loss of mammals’ functional diversity with future species extinctions. Glob. Chang. Biol. 30, e17375 (2024). 38895806 10.1111/gcb.17375

[12] R. Sayre, S. Noble, S. Hamann, R. Smith, D. Wright, S. Breyer, K. Butler, K. Van Graafeiland, C. Frye, D. Karagulle, D. Hopkins, D. Stephens, K. Kelly, Z. Basher, D. Burton, J. Cress, K. Atkins, D. P. Van Sistine, B. Friesen, R. Allee, T. Allen, P. Aniello, I. Asaad, M. J. Costello, K. Goodin, P. Harris, M. Kavanaugh, H. Lillis, E. Manca, F. Muller-Karger, B. Nyberg, R. Parsons, J. Saarinen, J. Steiner, A. Reed, A new 30 meter resolution global shoreline vector and associated global islands database for the development of standardized ecological coastal units. J. Oper. Oceanogr. 12, S47–S56 (2019).

[13] G. Kier, H. Kreft, T. M. Lee, W. Jetz, P. L. Ibisch, C. Nowicki, J. Mutke, W. Barthlott, A global assessment of endemism and species richness across island and mainland regions. Proc. Natl. Acad. Sci. U.S.A. 106, 9322–9327 (2009). 19470638 10.1073/pnas.0810306106 PMC2685248

[14] B. R. Tershy, K.-W. Shen, K. M. Newton, N. D. Holmes, D. A. Croll, The importance of islands for the protection of biological and linguistic diversity. BioScience 65, 592–597 (2015).

[15] K. E. Saban, J. J. Wiens, Unpacking the extinction crisis: Rates, patterns and causes of recent extinctions in plants and animals. Proc. R. Soc. B. Biol. Sci. 292, 20251717 (2025). 10.1098/rspb.2025.1717 PMC12520767 41086849

[16] T. Roedl, S. Berger, L. M. Romero, M. Wikelski, Tameness and stress physiology in a predator-naive island species confronted with novel predation threat. Proc. R. Soc. B. Biol. Sci. 274, 577–582 (2007). 10.1098/rspb.2006.3755 PMC1766385 17476779

[17] K. C. Rosenblad, D. L. Perret, D. F. Sax, Niche syndromes reveal climate-driven extinction threat to island endemic conifers. Nat. Clim. Chang. 9, 627–631 (2019).

[18] S. Baeckens, R. Van Damme, The island syndrome. Curr. Biol. 30, R338–R339 (2020). 32315628 10.1016/j.cub.2020.03.029

[19] R. J. Whittaker, J. M. Fernández-Palacios, T. J. Matthews, M. K. Borregaard, K. A. Triantis, Island biogeography: Taking the long view of nature’s laboratories. Science 357, eaam8326 (2017). 28860356 10.1126/science.aam8326

[20] J. R. Wood, J. A. Alcover, T. M. Blackburn, P. Bover, R. P. Duncan, J. P. Hume, J. Louys, H. J. M. Meijer, J. C. Rando, J. M. Wilmshurst, Island extinctions: Processes, patterns, and potential for ecosystem restoration. Environ. Conserv. 44, 348–358 (2017).

[21] The IUCN Red List of Threatened Species, www.iucnredlist.org.

[22] T. J. Matthews, J. P. Wayman, P. Cardoso, F. Sayol, J. P. Hume, W. Ulrich, J. A. Tobias, F. C. Soares, C. Thébaud, T. E. Martin, K. A. Triantis, Threatened and extinct island endemic birds of the world: Distribution, threats and functional diversity. J. Biogeogr. 49, 1920–1940 (2022).

[23] P. Pysek, P. E. Hulme, D. Simberloff, S. Bacher, T. M. Blackburn, J. T. Carlton, W. Dawson, F. Essl, L. C. Foxcroft, P. Genovesi, J. M. Jeschke, I. Kuehn, A. M. Liebhold, N. E. Mandrak, L. A. Meyerson, A. Pauchard, J. Pergl, H. E. Roy, H. Seebens, M. van Kleunen, M. Vila, M. J. Wingfield, D. M. Richardson, Scientists’ warning on invasive alien species. Biol. Rev. 95, 1511–1534 (2020). 32588508 10.1111/brv.12627 PMC7687187

[24] C. J. Brandon, W. D. Pearse, J. P. Herrera, The intrinsic and extrinsic drivers of extinction risk in lemurs (Lemuroidea). Biol. Conserv. 290, 110408 (2024).

[25] M. Cardillo, G. M. Mace, K. E. Jones, J. Bielby, O. R. P. Bininda-Emonds, W. Sechrest, C. D. L. Orme, A. Purvis, Multiple causes of high extinction risk in large mammal species. Science 309, 1239–1241 (2005). 16037416 10.1126/science.1116030

[26] S. Ducatez, R. Shine, Drivers of extinction risk in terrestrial vertebrates. Conserv. Lett. 10, 186–194 (2017).

[27] W. J. Ripple, C. Wolf, T. M. Newsome, M. Hoffmann, A. J. Wirsing, D. J. McCauley, Extinction risk is most acute for the world’s largest and smallest vertebrates. Proc. Natl. Acad. Sci. U.S.A. 114, 10678–10683 (2017). 28923917 10.1073/pnas.1702078114 PMC5635868

[28] A. G. Boyer, Consistent ecological selectivity through time in pacific island avian extinctions. Conserv. Biol. 24, 511–519 (2010). 19843128 10.1111/j.1523-1739.2009.01341.x

[29] R. Rozzi, M. V. Lomolino, A. A. E. Van Der Geer, D. Silvestro, S. K. Lyons, P. Bover, J. A. Alcover, A. Benítez-López, C.-H. Tsai, M. Fujita, M. O. Kubo, J. Ochoa, M. E. Scarborough, S. T. Turvey, A. Zizka, J. M. Chase, Dwarfism and gigantism drive human-mediated extinctions on islands. Science 379, 1054–1059 (2023). 36893233 10.1126/science.add8606

[30] Y. Xiong, R. Rozzi, Y. Zhang, L. Fan, J. Zhao, D. Li, Y. Yao, H. Xiao, J. Liu, X. Zeng, H. Xu, Y. Jiang, F. Lei, Convergent evolution toward a slow pace of life predisposes insular endotherms to anthropogenic extinctions. Sci. Adv. 10, eadm8240 (2024). 38996028 10.1126/sciadv.adm8240 PMC11244536

[31] R. Cooke, F. Sayol, T. Andermann, T. M. Blackburn, M. J. Steinbauer, A. Antonelli, S. Faurby, Undiscovered bird extinctions obscure the true magnitude of human-driven extinction waves. Nat. Commun. 14, 8116 (2023). 38114469 10.1038/s41467-023-43445-2 PMC10730700

[32] E. Barreto, T. F. Rangel, L. Pellissier, C. H. Graham, Area, isolation and climate explain the diversity of mammals on islands worldwide. Proc. R. Soc. B. Biol. Sci. 288, 20211879 (2021). 10.1098/rspb.2021.1879 PMC8670959 34905709

[33] J. Foufopoulos, A. M. Kilpatrick, A. R. Ives, Climate change and elevated extinction rates of reptiles from mediterranean islands. Am. Nat. 177, 119–129 (2011). 21091198 10.1086/657624 PMC3104022

[34] F. Orgeret, A. Thiebault, K. M. Kovacs, C. Lydersen, M. A. Hindell, S. A. Thompson, W. J. Sydeman, P. A. Pistorius, Climate change impacts on seabirds and marine mammals: The importance of study duration, thermal tolerance and generation time. Ecol. Lett. 25, 218–239 (2022). 34761516 10.1111/ele.13920

[35] R. L. White, P. M. Bennett, Elevational distribution and extinction risk in birds. PLOS ONE 10, e0121849 (2015). 25849620 10.1371/journal.pone.0121849 PMC4388662

[36] M. Böhm, R. Williams, H. R. Bramhall, K. M. McMillan, A. D. Davidson, A. Garcia, L. M. Bland, J. Bielby, B. Collen, Correlates of extinction risk in squamate reptiles: The relative importance of biology, geography, threat and range size. Glob. Ecol. Biogeogr. 25, 391–405 (2016).

[37] J. Guirguis, L. E. B. Goodyear, C. Finn, J. V. Johnson, D. Pincheira-Donoso, Risk of extinction increases towards higher elevations across the world’s amphibians. Glob. Ecol. Biogeogr. 32, 1952–1963 (2023).

[38] A. A. Hoffmann, C. M. Sgrò, Climate change and evolutionary adaptation. Nature 470, 479–485 (2011). 21350480 10.1038/nature09670

[39] C. Chen, J. Wang, M. Holyoak, L. Lin, Y. Wang, Global assessment of current extinction risks and future challenges for turtles and tortoises. Nat. Commun. 16, 7114 (2025). 40753074 10.1038/s41467-025-62441-2 PMC12318056

[40] P. H. Verburg, K. Neumann, L. Nol, Challenges in using land use and land cover data for global change studies. Glob. Chang. Biol. 17, 974–989 (2011).

[41] R. Tingley, R. A. Hitchmough, D. G. Chapple, Life-history traits and extrinsic threats determine extinction risk in New Zealand lizards. Biol. Conserv. 165, 62–68 (2013).

[42] S. Nogué, A. M. C. Santos, H. J. B. Birks, S. Björck, A. Castilla-Beltrán, S. Connor, E. J. de Boer, L. de Nascimento, V. A. Felde, J. M. Fernández-Palacios, C. A. Froyd, S. G. Haberle, H. Hooghiemstra, K. Ljung, S. J. Norder, J. Peñuelas, M. Prebble, J. Stevenson, R. J. Whittaker, K. J. Willis, J. M. Wilmshurst, M. J. Steinbauer, The human dimension of biodiversity changes on islands. Science 372, 488–491 (2021). 33926949 10.1126/science.abd6706

[43] T. M. Blackburn, P. Cassey, R. P. Duncan, K. L. Evans, K. J. Gaston, Avian extinction and mammalian introductions on oceanic islands. Science 305, 1955–1958 (2004). 15448269 10.1126/science.1101617

[44] D. R. Spatz, K. M. Zilliacus, N. D. Holmes, S. H. M. Butchart, P. Genovesi, G. Ceballos, B. R. Tershy, D. A. Croll, Globally threatened vertebrates on islands with invasive species. Sci. Adv. 3, e1603080 (2017). 29075662 10.1126/sciadv.1603080 PMC5656423

[45] K. Liversage, J. Kotta, I. Kuprijanov, M. Rätsep, K. Nõomaa, A trophic cascade facilitates native habitat providers within assemblages of multiple invasive marine species. Ecosphere 12, e03621 (2021).

[46] D. A. Croll, J. L. Maron, J. A. Estes, E. M. Danner, G. V. Byrd, Introduced predators transform subarctic islands from grassland to tundra. Science 307, 1959–1961 (2005). 15790855 10.1126/science.1108485

[47] J. H. Heinen, E. E. Van Loon, D. M. Hansen, W. D. Kissling, Extinction-driven changes in frugivore communities on oceanic islands. Ecography 41, 1245–1255 (2018).

[48] J. H. Heinen, F. B. V. Florens, C. Baider, J. P. Hume, W. D. Kissling, R. J. Whittaker, C. Rahbek, M. K. Borregaard, Novel plant–frugivore network on Mauritius is unlikely to compensate for the extinction of seed dispersers. Nat. Commun. 14, 1019 (2023). 36823195 10.1038/s41467-023-36669-9 PMC9950440

[49] B. C. Scheele, F. Pasmans, L. F. Skerratt, L. Berger, A. Martel, W. Beukema, A. A. Acevedo, P. A. Burrowes, T. Carvalho, A. Catenazzi, I. De la Riva, M. C. Fisher, S. V. Flechas, C. N. Foster, P. Frías-Álvarez, T. W. J. Garner, B. Gratwicke, J. M. Guayasamin, M. Hirschfeld, J. E. Kolby, T. A. Kosch, E. La Marca, D. B. Lindenmayer, K. R. Lips, A. V. Longo, R. Maneyro, C. A. McDonald, J. Mendelson, P. Palacios-Rodriguez, G. Parra-Olea, C. L. Richards-Zawacki, M.-O. Rödel, S. M. Rovito, C. Soto-Azat, L. F. Toledo, J. Voyles, C. Weldon, S. M. Whitfield, M. Wilkinson, K. R. Zamudio, S. Canessa, Amphibian fungal panzootic causes catastrophic and ongoing loss of biodiversity. Science 363, 1459–1463 (2019). 30923224 10.1126/science.aav0379

[50] J. A. Luedtke, J. Chanson, K. Neam, L. Hobin, A. O. Maciel, A. Catenazzi, A. Borzée, A. Hamidy, A. Aowphol, A. Jean, Á. Sosa-Bartuano, A. Fong G, A. de Silva, A. Fouquet, A. Angulo, A. A. Kidov, A. M. Saravia, A. C. Diesmos, A. Tominaga, B. Shrestha, B. Gratwicke, B. Tjaturadi, C. C. M. Rivera, C. R. V. Almazán, C. Señaris, S. R. Chandramouli, C. Strüssmann, C. F. C. Fernández, C. Azat, C. J. Hoskin, C. Hilton-Taylor, D. L. Whyte, D. J. Gower, D. H. Olson, D. F. Cisneros-Heredia, D. J. Santana, E. Nagombi, E. Najafi-Majd, E. S. H. Quah, F. Bolaños, F. Xie, F. Brusquetti, F. S. Álvarez, F. Andreone, F. Glaw, F. E. Castañeda, F. Kraus, G. Parra-Olea, G. Chaves, G. F. Medina-Rangel, G. González-Durán, H. M. Ortega-Andrade, I. F. Machado, I. Das, I. R. Dias, J. N. Urbina-Cardona, J. Crnobrnja-Isailović, J.-H. Yang, J. Jianping, J. T. Wangyal, J. J. L. Rowley, J. Measey, K. Vasudevan, K. O. Chan, K. V. Gururaja, K. Ovaska, L. C. Warr, L. Canseco-Márquez, L. F. Toledo, L. M. Díaz, M. M. H. Khan, M. Meegaskumbura, M. E. Acevedo, M. F. Napoli, M. A. Ponce, M. Vaira, M. Lampo, M. H. Yánez-Muñoz, M. D. Scherz, M.-O. Rödel, M. Matsui, M. Fildor, M. D. Kusrini, M. F. Ahmed, M. Rais, N. G. Kouamé, N. García, N. L. Gonwouo, P. A. Burrowes, P. Y. Imbun, P. Wagner, P. J. R. Kok, R. L. Joglar, R. J. Auguste, R. A. Brandão, R. Ibáñez, R. von May, S. B. Hedges, S. D. Biju, S. R. Ganesh, S. Wren, S. Das, S. V. Flechas, S. L. Ashpole, S. J. Robleto-Hernández, S. P. Loader, S. J. Incháustegui, S. Garg, S. Phimmachak, S. J. Richards, T. Slimani, T. Osborne-Naikatini, T. P. F. Abreu-Jardim, T. H. Condez, T. R. De Carvalho, T. P. Cutajar, T. W. Pierson, T. Q. Nguyen, U. Kaya, Z. Yuan, B. Long, P. Langhammer, S. N. Stuart, Ongoing declines for the world’s amphibians in the face of emerging threats. Nature 622, 308–314 (2023). 37794184 10.1038/s41586-023-06578-4 PMC10567568

[51] F. Gonçalves, H. Farooq, M. Harfoot, M. M. Pires, N. Villar, L. Sales, C. Carvalho, C. Bello, C. Emer, R. S. Bovendorp, C. Mendes, G. Beca, L. Lautenschlager, Y. Souza, F. Pedrosa, C. Paz, V. B. Zipparro, P. Akkawi, W. Bercê, F. Farah, A. V. L. Freitas, L. F. Silveira, F. Olmos, J. Geldmann, B. Dalsgaard, M. Galetti, A global map of species at risk of extinction due to natural hazards. Proc. Natl. Acad. Sci. U.S.A. 121, e2321068121 (2024). 38885390 10.1073/pnas.2321068121 PMC11214083

[52] G. F. Ficetola, Habitat conservation research for amphibians: Methodological improvements and thematic shifts. Biodivers. Conserv. 24, 1293–1310 (2015).

[53] J. R. Wood, J. M. Wilmshurst, T. H. Worthy, A. S. Holzapfel, A. Cooper, A lost link between a flightless parrot and a parasitic plant and the potential role of coprolites in conservation paleobiology. Conserv. Biol. 26, 1091–1099 (2012). 23025275 10.1111/j.1523-1739.2012.01931.x

[54] T. J. Matthews, K. A. Triantis, J. P. Wayman, T. E. Martin, J. P. Hume, P. Cardoso, S. Faurby, C. D. Mendenhall, P. Dufour, F. Rigal, R. Cooke, R. J. Whittaker, A. L. Pigot, C. Thébaud, M. W. Jørgensen, E. Benavides, F. C. Soares, W. Ulrich, Y. Kubota, J. P. Sadler, J. A. Tobias, F. Sayol, The global loss of avian functional and phylogenetic diversity from anthropogenic extinctions. Science 386, 55–60 (2024). 39361743 10.1126/science.adk7898

[55] M. Galetti, R. Guevara, M. C. Côrtes, R. Fadini, S. Von Matter, A. B. Leite, F. Labecca, T. Ribeiro, C. S. Carvalho, R. G. Collevatti, M. M. Pires, P. R. Guimarães, P. H. Brancalion, M. C. Ribeiro, P. Jordano, Functional extinction of birds drives rapid evolutionary changes in seed size. Science 340, 1086–1090 (2013). 23723235 10.1126/science.1233774

[56] R. M. Pringle, J. O. Abraham, T. M. Anderson, T. C. Coverdale, A. B. Davies, C. L. Dutton, A. Gaylard, J. R. Goheen, R. M. Holdo, M. C. Hutchinson, D. M. Kimuyu, R. A. Long, A. L. Subalusky, M. P. Veldhuis, Impacts of large herbivores on terrestrial ecosystems. Curr. Biol. 33, R584–R610 (2023). 37279691 10.1016/j.cub.2023.04.024

[57] R. Dirzo, H. S. Young, M. Galetti, G. Ceballos, N. J. B. Isaac, B. Collen, Defaunation in the anthropocene. Science 345, 401–406 (2014). 25061202 10.1126/science.1251817

[58] H. S. Young, D. J. McCauley, M. Galetti, R. Dirzo, Patterns, causes, and consequences of anthropocene defaunation. Annu. Rev. Ecol. Evol. Syst. 47, 333–358 (2016).

[59] K. R. McConkey, D. R. Drake, Low redundancy in seed dispersal within an island frugivore community. AoB Plants 7, plv088 (2015). 26194167 10.1093/aobpla/plv088 PMC4583771

[60] K. A. Wilson, M. F. McBride, M. Bode, H. P. Possingham, Prioritizing global conservation efforts. Nature 440, 337–340 (2006). 16541073 10.1038/nature04366

[61] D. Simberloff, L. G. Abele, Refuge design and island biogeographic theory: Effects of fragmentation. Am. Nat. 120, 41–50 (1982).

[62] L. Fahrig, J. I. Watling, C. A. Arnillas, V. Arroyo-Rodríguez, T. Jörger-Hickfang, J. Müller, H. M. Pereira, F. Riva, V. Rösch, S. Seibold, T. Tscharntke, F. May, Resolving the SLOSS dilemma for biodiversity conservation: A research agenda. Biol. Rev. 97, 99–114 (2022). 34453405 10.1111/brv.12792 PMC9290967

[63] R. J. Whittaker, J. M. Fernández-Palacios, T. J. Matthews, “The biogeography of island life: Biodiversity hotspots in context,” in *Island Biogeography: Geo-Environmental Dynamics, Ecology, Evolution, Human Impact, and Conservation*, R. J. Whittaker, J. M. Fernández-Palacios, T. J. Matthews, Eds. (Oxford Univ. Press, 2023), 57–90.

[64] Y. Li, Y. Wang, X. Liu, Half of global islands have reached critical area thresholds for undergoing rapid increases in biological invasions. Proc. R. Soc. B. Biol. Sci. 291, rspb.2024.0844 (2024). 10.1098/rspb.2024.0844 PMC11285800 38889781

[65] G. Li, C. Fang, J. E. M. Watson, S. Sun, W. Qi, Z. Wang, J. Liu, Mixed effectiveness of global protected areas in resisting habitat loss. Nat. Commun. 15, 8389 (2024). 39333073 10.1038/s41467-024-52693-9 PMC11437083

[66] M. T. Jezierski, W. J. Smith, S. M. Clegg, The island syndrome in birds. J. Biogeogr. 51, 1607–1622 (2024).

[67] M. J. A. Creighton, C. L. Nunn, Explaining the primate extinction crisis: Predictors of extinction risk and active threats. Proc. R. Soc. B. Biol. Sci. 290, 20231441 (2023). 10.1098/rspb.2023.1441 PMC10510445 37670584

[68] D. M. Hansen, C. J. Donlan, C. J. Griffiths, K. J. Campbell, Ecological history and latent conservation potential: Large and giant tortoises as a model for taxon substitutions. Ecography 33, 272–284 (2010).

[69] G. Olah, S. H. M. Butchart, A. Symes, I. M. Guzmán, R. Cunningham, D. J. Brightsmith, R. Heinsohn, Ecological and socio-economic factors affecting extinction risk in parrots. Biodivers. Conserv. 25, 205–223 (2016).

[70] J.-M. Roberge, P. Angelstam, Usefulness of the umbrella species concept as a conservation tool. Conserv. Biol. 18, 76–85 (2004).

[71] M. Branton, J. S. Richardson, Assessing the value of the umbrella-species concept for conservation planning with meta-analysis. Conserv. Biol. 25, 9–20 (2011). 21091767 10.1111/j.1523-1739.2010.01606.x

[72] I. Breckheimer, N. M. Haddad, W. F. Morris, A. M. Trainor, W. R. Fields, R. T. Jobe, B. R. Hudgens, A. Moody, J. R. Walters, Defining and evaluating the umbrella species concept for conserving and restoring landscape connectivity. Conserv. Biol. 28, 1584–1593 (2014). 25115148 10.1111/cobi.12362

[73] N. S. Upham, Past and present of insular Caribbean mammals: Understanding Holocene extinctions to inform modern biodiversity conservation. J. Mammal. 98, 913–917 (2017).

[74] P. Pottier, M. R. Kearney, N. C. Wu, A. R. Gunderson, J. E. Rej, A. N. Rivera-Villanueva, P. Pollo, S. Burke, S. M. Drobniak, S. Nakagawa, Vulnerability of amphibians to global warming. Nature 639, 954–961 (2025). 40044855 10.1038/s41586-025-08665-0 PMC11946914

[75] C. Leclerc, F. Courchamp, C. Bellard, Insular threat associations within taxa worldwide. Sci. Rep. 8, 6393 (2018). 29686360 10.1038/s41598-018-24733-0 PMC5913315

[76] M. J. Munstermann, N. A. Heim, D. J. McCauley, J. L. Payne, N. S. Upham, S. C. Wang, M. L. Knope, A global ecological signal of extinction risk in terrestrial vertebrates. Conserv. Biol. 36, e13852 (2022). 34668599 10.1111/cobi.13852 PMC9299904

[77] G. M. Mace, N. J. Collar, K. J. Gaston, C. Hilton-Taylor, H. R. Akçakaya, N. Leader-Williams, E. J. Milner-Gulland, S. N. Stuart, Quantification of extinction risk: IUCN’s system for classifying threatened species. Conserv. Biol. 22, 1424–1442 (2008). 18847444 10.1111/j.1523-1739.2008.01044.x

[78] J. Borgelt, M. Dorber, M. A. Høiberg, F. Verones, More than half of data deficient species predicted to be threatened by extinction. Commun. Biol. 5, 679 (2022). 35927327 10.1038/s42003-022-03638-9 PMC9352662

[79] I. R. Geijzendorffer, E. C. Regan, H. M. Pereira, L. Brotons, N. Brummitt, Y. Gavish, P. Haase, C. S. Martin, J.-B. Mihoub, C. Secades, D. S. Schmeller, S. Stoll, F. T. Wetzel, M. Walters, Bridging the gap between biodiversity data and policy reporting needs: An essential biodiversity variables perspective. J. Appl. Ecol. 53, 1341–1350 (2016).

[80] R. H. MacArthur, E. O. Wilson, *The Theory of Island Biogeography* (Princeton Univ. Press, 1967).

[81] R. Frankham, D. A. Briscoe, J. D. Ballou, *Introduction to Conservation Genetics* (Cambridge Univ. Press, 2012).

[82] Y. Wang, X. Si, P. M. Bennett, C. Chen, D. Zeng, Y. Zhao, Y. Wu, P. Ding, Ecological correlates of extinction risk in Chinese birds. Ecography 41, 782–794 (2018).

[83] S. Zhang, S. Meiri, M. Holyoak, J. Wang, Y. Wang, C. Chen, Demand for small- and large-ranged reptiles in worldwide wildlife trade. Conserv. Biol. 39, e70095 (2025). 40551634 10.1111/cobi.70095

[84] R. K. Kopf, S. Banks, L. J. N. Brent, P. Humphries, C. J. Jolly, P. C. Lee, O. J. Luiz, D. Nimmo, K. O. Winemiller, Loss of Earth’s old, wise, and large animals. Science 387, eado2705 (2025). 39571003 10.1126/science.ado2705

[85] M. Cardillo, Clarifying the relationship between body size and extinction risk in amphibians by complete mapping of model space. Proc. R. Soc. B. Biol. Sci. 288, 20203011 (2021). 10.1098/rspb.2020.3011 PMC7893221 33529561

[86] S. Matsui, M. Hisaka, M. Takagi, Arboreal nesting and utilization of open-cup bird nests by introduced Ship Rats *Rattus rattus* on an oceanic island. Bird Conserv. Int. 20, 34–42 (2010).

[87] A. H. Nance, W. F. Mitchell, F. Dawlings, C. N. Cook, R. H. Clarke, Rodent predation and specialised avian habitat requirements drive extinction risk for endemic island songbirds in the south-west Pacific. Emu 123, 217–231 (2023).

[88] J. M. Hoare, S. Pledger, N. J. Nelson, C. H. Daugherty, Avoiding aliens: Behavioural plasticity in habitat use enables large, nocturnal geckos to survive Pacific rat invasions. Biol. Conserv. 136, 510–519 (2007).

[89] N. P. Mohanty, S. Harikrishnan, K. Vasudevan, Watch out where you sleep: Nocturnal sleeping behaviour of Bay Island lizards. PeerJ 4, e1856 (2016). 27168958 10.7717/peerj.1856 PMC4860295

[90] S. Baeckens, S. Meiri, R. Shine, Foraging mode affects extinction risk of snakes and lizards, but in different ways. Conserv. Lett. 16, e12977 (2023).

[91] F. Chichorro, F. Urbano, D. Teixeira, H. Väre, T. Pinto, N. Brummitt, X. He, A. Hochkirch, J. Hyvönen, L. Kaila, A. Juslén, P. Cardoso, Trait-based prediction of extinction risk across terrestrial taxa. Biol. Conserv. 274, 109738 (2022).

[92] F. Sayol, M. J. Steinbauer, T. M. Blackburn, A. Antonelli, S. Faurby, Anthropogenic extinctions conceal widespread evolution of flightlessness in birds. Sci. Adv. 6, eabb6095 (2020). 33268368 10.1126/sciadv.abb6095 PMC7710364

[93] T. P. Leppard, E. E. Cochrane, D. Gaffney, C. L. Hofman, J. E. Laffoon, M. M. E. Bunbury, C. Broodbank, Global patterns in island colonization during the holocene. J. World Prehist. 35, 163–232 (2022).

[94] W. Beukema, F. Pasmans, S. Van Praet, F. Ferri-Yáñez, M. Kelly, A. E. Laking, J. Erens, J. Speybroeck, K. Verheyen, L. Lens, A. Martel, Microclimate limits thermal behaviour favourable to disease control in a nocturnal amphibian. Ecol. Lett. 24, 27–37 (2021). 33022129 10.1111/ele.13616

[95] S. F. Nunes, K. Powell, P. Griffith, M. A. Carretero, R. Rebelo, M. Cabeza, R. Rocha, Island-restricted reptiles are more threatened but less studied than their mainland counterparts. Conservat. Sci. and Prac. 8, e70184 (2025).

[96] W. E. Cooper, R. A. Pyron, T. Garland, Island tameness: Living on islands reduces flight initiation distance. Proc. R. Soc. B. Biol. Sci. 281, 20133019 (2014). 10.1098/rspb.2013.3019 PMC3896029 24403345

[97] G. C. Cummins, T. C. Theimer, E. H. Paxton, Responses to terrestrial nest predators by endemic and introduced Hawaiian birds. Ecol. Evol. 10, 1949–1958 (2020). 32128128 10.1002/ece3.6021 PMC7042753

[98] E. Montes, F. Kraus, B. Chergui, J. M. Pleguezuelos, Collapse of the endemic lizard *Podarcis pityusensis* on the island of Ibiza mediated by an invasive snake. Curr. Zool. 68, 295–303 (2022). 35592342 10.1093/cz/zoab022 PMC9113342

[99] H. P. Jones, N. D. Holmes, S. H. M. Butchart, B. R. Tershy, P. J. Kappes, I. Corkery, A. Aguirre-Muñoz, D. P. Armstrong, E. Bonnaud, A. A. Burbidge, K. Campbell, F. Courchamp, P. E. Cowan, R. J. Cuthbert, S. Ebbert, P. Genovesi, G. R. Howald, B. S. Keitt, S. W. Kress, C. M. Miskelly, S. Oppel, S. Poncet, M. J. Rauzon, G. Rocamora, J. C. Russell, A. Samaniego-Herrera, P. J. Seddon, D. R. Spatz, D. R. Towns, D. A. Croll, Invasive mammal eradication on islands results in substantial conservation gains. Proc. Natl. Acad. Sci. U.S.A. 113, 4033–4038 (2016). 27001852 10.1073/pnas.1521179113 PMC4839448

[100] M. M. A. Boehm, Q. C. B. Cronk, Dark extinction: The problem of unknown historical extinctions. Biol. Lett. 17, 20210007 (2021). 33653097 10.1098/rsbl.2021.0007 PMC8086982

[101] B. Leung, A. Gonzalez, Global monitoring for biodiversity: Uncertainty, risk, and power analyses to support trend change detection. Sci. Adv. 10, eadj1448 (2024). 38363843 10.1126/sciadv.adj1448 PMC11639671

[102] K. A. Triantis, P. A. V. Borges, R. J. Ladle, J. Hortal, P. Cardoso, C. Gaspar, F. Dinis, E. Mendonça, L. M. A. Silveira, R. Gabriel, C. Melo, A. M. C. Santos, I. R. Amorim, S. P. Ribeiro, A. R. M. Serrano, J. A. Quartau, R. J. Whittaker, Extinction debt on oceanic islands. Ecography 33, 285–294 (2010).

[103] J. Troudet, P. Grandcolas, A. Blin, R. Vignes-Lebbe, F. Legendre, Taxonomic bias in biodiversity data and societal preferences. Sci. Rep. 7, 9132 (2017). 28831097 10.1038/s41598-017-09084-6 PMC5567328

[104] Y. Fourcade, Comparing species distributions modelled from occurrence data and from expert-based range maps. Implication for predicting range shifts with climate change. Ecol. Inform. 36, 8–14 (2016).

[105] V. Cazalis, L. Santini, P. M. Lucas, M. González-Suárez, M. Hoffmann, A. Benítez-López, M. Pacifici, A. M. Schipper, M. Böhm, A. Zizka, V. Clausnitzer, C. Meyer, M. Jung, S. H. M. Butchart, P. Cardoso, G. Mancini, H. R. Akçakaya, B. E. Young, G. Patoine, M. Di Marco, Prioritizing the reassessment of data-deficient species on the IUCN Red List. Conserv. Biol. 37, e14139 (2023). 37394972 10.1111/cobi.14139

[106] S. Chamberlain, rredlist: “IUCN” Red List Client, version 0.7.1 (2022); https://cran.r-project.org/web/packages/rredlist/index.html.

[107] Natural Earth, Free vector and raster map data at 1:10m, 1:50m, and 1:110m scales; www.naturalearthdata.com/.

[108] P. Weigelt, W. Jetz, H. Kreft, Bioclimatic and physical characterization of the world’s islands. Proc. Natl. Acad. Sci. U.S.A. 110, 15307–15312 (2013). 24003123 10.1073/pnas.1306309110 PMC3780862

[109] BirdLife International, www.birdlife.org/.

[110] U. Roll, A. Feldman, M. Novosolov, A. Allison, A. M. Bauer, R. Bernard, M. Böhm, F. Castro-Herrera, L. Chirio, B. Collen, G. R. Colli, L. Dabool, I. Das, T. M. Doan, L. L. Grismer, M. Hoogmoed, Y. Itescu, F. Kraus, M. LeBreton, A. Lewin, M. Martins, E. Maza, D. Meirte, Z. T. Nagy, C. De, C. Nogueira, O. S. G. Pauwels, D. Pincheira-Donoso, G. D. Powney, R. Sindaco, O. J. S. Tallowin, O. Torres-Carvajal, J.-F. Trape, E. Vidan, P. Uetz, P. Wagner, Y. Wang, C. D. L. Orme, R. Grenyer, S. Meiri, The global distribution of tetrapods reveals a need for targeted reptile conservation. Nat. Ecol. Evol. 1, 1677–1682 (2017). 28993667 10.1038/s41559-017-0332-2

[111] Q. Huang, J. R. Sauer, A. Swatantran, R. Dubayah, A centroid model of species distribution with applications to the Carolina wren *Thryothorus ludovicianus* and house finch *Haemorhous mexicanus* in the United States. Ecography 39, 54–66 (2016).

[112] Y. Sun, Y. Deng, S. Yao, Y. Sun, A. A. Degen, L. Dong, J. Luo, S. Xie, Q. Hou, D. Tang, Y. Sun, J. Xiong, J. Peng, W. Hu, J. Ran, J. Deng, Distribution range and richness of plant species are predicted to increase by 2100 due to a warmer and wetter climate in Northern China. Glob. Chang. Biol. 31, e70334 (2025). 40613311 10.1111/gcb.70334 PMC12232221

[113] R. Sokal, F. Rohlf, *Biometry: The Principles and Practice of Statistics in Biological Research* (W. H. Freeman & Company, ed. 2, 2012).

[114] Z. Šidák, Rectangular confidence regions for the means of multivariate normal distributions. J. Am. Stat. Assoc. 62, 626–633 (1967).

[115] O. J. Dunn, Multiple comparisons among means. J. Am. Stat. Assoc. 56, 52–64 (1961).

[116] M. R. Moura, K. Ceron, J. J. M. Guedes, R. Chen-Zhao, Y. V. Sica, J. Hart, W. Dorman, J. M. Portmann, P. González-del-Pliego, A. Ranipeta, A. Catenazzi, F. P. Werneck, L. F. Toledo, N. S. Upham, J. F. R. Tonini, T. J. Colston, R. Guralnick, R. C. K. Bowie, R. A. Pyron, W. Jetz, A phylogeny-informed characterisation of global tetrapod traits addresses data gaps and biases. PLoS Biol. 22, e3002658 (2024). 38991106 10.1371/journal.pbio.3002658 PMC11239118

[117] B. F. Oliveira, V. A. São-Pedro, G. Santos-Barrera, C. Penone, G. C. Costa, AmphiBIO, a global database for amphibian ecological traits. Sci. Data 4, 170123 (2017). 28872632 10.1038/sdata.2017.123 PMC5584397

[118] J. P. Bird, R. Martin, H. R. Akçakaya, J. Gilroy, I. J. Burfield, S. T. Garnett, A. Symes, J. Taylor, Ç. H. Şekercioğlu, S. H. M. Butchart, Generation lengths of the world’s birds and their implications for extinction risk. Conserv. Biol. 34, 1252–1261 (2020). 32058610 10.1111/cobi.13486

[119] C. D. Soria, M. Pacifici, M. Di Marco, S. M. Stephen, C. Rondinini, COMBINE: A coalesced mammal database of intrinsic and extrinsic traits. Ecology 102, e03344 (2021). 33742448 10.1002/ecy.3344

[120] J. A. Tobias, C. Sheard, A. L. Pigot, A. J. M. Devenish, J. Yang, F. Sayol, M. H. C. Neate-Clegg, N. Alioravainen, T. L. Weeks, R. A. Barber, P. A. Walkden, H. E. A. MacGregor, S. E. I. Jones, C. Vincent, A. G. Phillips, N. M. Marples, F. A. Montaño-Centellas, V. Leandro-Silva, S. Claramunt, B. Darski, B. G. Freeman, T. P. Bregman, C. R. Cooney, E. C. Hughes, E. J. R. Capp, Z. K. Varley, N. R. Friedman, H. Korntheuer, A. Corrales-Vargas, C. H. Trisos, B. C. Weeks, D. M. Hanz, T. Töpfer, G. A. Bravo, V. Remeš, L. Nowak, L. S. Carneiro, A. J. Moncada R., B. Matysioková, D. T. Baldassarre, A. Martínez-Salinas, J. D. Wolfe, P. M. Chapman, B. G. Daly, M. C. Sorensen, A. Neu, M. A. Ford, R. J. Mayhew, L. F. Silveira, D. J. Kelly, N. N. D. Annorbah, H. S. Pollock, A. M. Grabowska-Zhang, J. P. McEntee, J. C. T. Gonzalez, C. G. Meneses, M. C. Muñoz, L. L. Powell, G. A. Jamie, T. J. Matthews, O. Johnson, G. R. R. Brito, K. Zyskowski, R. Crates, M. G. Harvey, M. J. Zevallos, P. A. Hosner, T. Bradfer-Lawrence, J. M. Maley, F. G. Stiles, H. S. Lima, K. L. Provost, M. Chibesa, M. Mashao, J. T. Howard, E. Mlamba, M. A. H. Chua, B. Li, M. I. Gómez, N. C. García, M. Päckert, J. Fuchs, J. R. Ali, E. P. Derryberry, M. L. Carlson, R. C. Urriza, K. E. Brzeski, D. M. Prawiradilaga, M. J. Rayner, E. T. Miller, R. C. K. Bowie, R.-M. Lafontaine, R. P. Scofield, Y. Lou, L. Somarathna, D. Lepage, M. Illif, E. L. Neuschulz, M. Templin, D. M. Dehling, J. C. Cooper, O. S. G. Pauwels, K. Analuddin, J. Fjeldså, N. Seddon, P. R. Sweet, F. A. J. DeClerck, L. N. Naka, J. D. Brawn, A. Aleixo, K. Böhning-Gaese, C. Rahbek, S. A. Fritz, G. H. Thomas, M. Schleuning, AVONET: Morphological, ecological and geographical data for all birds. Ecol. Lett. 25, 581–597 (2022). 35199922 10.1111/ele.13898

[121] F. Sayol, J. P. Wayman, P. Dufour, T. E. Martin, J. P. Hume, M. W. Jørgensen, N. Martínez-Rubio, A. Sanglas, F. C. Soares, R. Cooke, C. D. Mendenhall, J. R. Margolis, J. C. Illera, R. Lemoine, E. Benavides, O. Lapiedra, K. A. Triantis, A. L. Pigot, J. A. Tobias, S. Faurby, T. J. Matthews, AVOTREX: A global dataset of extinct birds and their traits. Glob. Ecol. Biogeogr. 33, e13927 (2024).

[122] O. Oskyrko, C. Mi, S. Meiri, W. Du, ReptTraits: A comprehensive dataset of ecological traits in reptiles. Sci. Data 11, 243 (2024). 38413613 10.1038/s41597-024-03079-5 PMC10899194

[123] G. Amatulli, S. Domisch, M.-N. Tuanmu, B. Parmentier, A. Ranipeta, J. Malczyk, W. Jetz, A suite of global, cross-scale topographic variables for environmental and biodiversity modeling. Sci. Data 5, 180040 (2018). 29557978 10.1038/sdata.2018.40 PMC5859920

[124] D. N. Karger, O. Conrad, J. Böhner, T. Kawohl, H. Kreft, R. W. Soria-Auza, N. E. Zimmermann, H. P. Linder, M. Kessler, Climatologies at high resolution for the earth’s land surface areas. Sci. Data 4, 170122 (2017). 28872642 10.1038/sdata.2017.122 PMC5584396

[125] H. Mu, X. Li, Y. Wen, J. Huang, P. Du, W. Su, S. Miao, M. Geng, A global record of annual terrestrial Human Footprint dataset from 2000 to 2018. Sci. Data 9, 176 (2022). 35440581 10.1038/s41597-022-01284-8 PMC9018937

[126] F. Gassert, O. Venter, J. E. M. Watson, S. P. Brumby, J. C. Mazzariello, S. C. Atkinson, S. Hyde, An operational approach to near real time global high resolution mapping of the terrestrial Human Footprint. Front. Remote Sens. 4, 1130896 (2023).

[127] E. E. Dyer, D. W. Redding, T. M. Blackburn, The global avian invasions atlas, a database of alien bird distributions worldwide. Sci. Data 4, 170041 (2017). 28350387 10.1038/sdata.2017.41 PMC5369319

[128] D. Biancolini, V. Vascellari, B. Melone, T. M. Blackburn, P. Cassey, S. L. Scrivens, C. Rondinini, DAMA: The global distribution of alien mammals database. Ecology 102, e03474 (2021). 34273183 10.1002/ecy.3474

[129] D. Wang, Y. Zhao, S. Tang, X. Liu, W. Li, P. Han, D. Zeng, Y. Yang, G. Wei, Y. Kang, X. Si, Nearby large islands diminish biodiversity of the focal island by a negative target effect. J. Anim. Ecol. 92, 492–502 (2023). 36478128 10.1111/1365-2656.13856

[130] G. Ceballos, P. R. Ehrlich, R. Dirzo, Biological annihilation via the ongoing sixth mass extinction signaled by vertebrate population losses and declines. Proc. Natl. Acad. Sci. U.S.A. 114, E6089–E6096 (2017). 28696295 10.1073/pnas.1704949114 PMC5544311

[131] J. Liu, F. Slik, S. Zheng, D. B. Lindenmayer, Undescribed species have higher extinction risk than known species. Conserv. Lett. 15, e12876 (2022).

[132] A. D. Davidson, M. J. Hamilton, A. G. Boyer, J. H. Brown, G. Ceballos, Multiple ecological pathways to extinction in mammals. Proc. Natl. Acad. Sci. U.S.A. 106, 10702–10705 (2009). 19528635 10.1073/pnas.0901956106 PMC2705575

[133] J. Yang, C. Yang, H. Lin, A. C. Lees, J. A. Tobias, Elevational constraints on flight efficiency shape global gradients in avian wing morphology. Curr. Biol. 35, 1890–1900.e5 (2025). 40120580 10.1016/j.cub.2025.02.068

[134] B. E. Crowley, L. R. Godfrey, R. J. Bankoff, G. H. Perry, B. J. Culleton, D. J. Kennett, M. R. Sutherland, K. E. Samonds, D. A. Burney, Island-wide aridity did not trigger recent megafaunal extinctions in Madagascar. Ecography 40, 901–912 (2017).

[135] S. van Buuren, K. Groothuis-Oudshoorn, mice: Multivariate imputation by chained equations in R. J. Stat. Softw. 45, 1–67 (2011).

[136] R. Molina-Venegas, How to get the most out of phylogenetic imputation without abusing it. Methods Ecol. Evol. 15, 456–463 (2024).

[137] P. Li, E. A. Stuart, D. B. Allison, Multiple imputation: A flexible tool for handling missing data. JAMA 314, 1966–1967 (2015). 26547468 10.1001/jama.2015.15281 PMC4638176

[138] J. Stiller, S. Feng, A.-A. Chowdhury, I. Rivas-González, D. A. Duchêne, Q. Fang, Y. Deng, A. Kozlov, A. Stamatakis, S. Claramunt, J. M. T. Nguyen, S. Y. W. Ho, B. C. Faircloth, J. Haag, P. Houde, J. Cracraft, M. Balaban, U. Mai, G. Chen, R. Gao, C. Zhou, Y. Xie, Z. Huang, Z. Cao, Z. Yan, H. A. Ogilvie, L. Nakhleh, B. Lindow, B. Morel, J. Fjeldså, P. A. Hosner, R. R. Da Fonseca, B. Petersen, J. A. Tobias, T. Székely, J. D. Kennedy, A. H. Reeve, A. Liker, M. Stervander, A. Antunes, D. T. Tietze, M. F. Bertelsen, F. Lei, C. Rahbek, G. R. Graves, M. H. Schierup, T. Warnow, E. L. Braun, M. T. P. Gilbert, E. D. Jarvis, S. Mirarab, G. Zhang, Complexity of avian evolution revealed by family-level genomes. Nature 629, 851–860 (2024). 38560995 10.1038/s41586-024-07323-1 PMC11111414

[139] N. S. Upham, J. A. Esselstyn, W. Jetz, Inferring the mammal tree: Species-level sets of phylogenies for questions in ecology, evolution, and conservation. PLoS Biol. 17, e3000494 (2019). 31800571 10.1371/journal.pbio.3000494 PMC6892540

[140] N. Cox, B. E. Young, P. Bowles, M. Fernandez, J. Marin, G. Rapacciuolo, M. Böhm, T. M. Brooks, S. B. Hedges, C. Hilton-Taylor, M. Hoffmann, R. K. B. Jenkins, M. F. Tognelli, G. J. Alexander, A. Allison, N. B. Ananjeva, M. Auliya, L. J. Avila, D. G. Chapple, D. F. Cisneros-Heredia, H. G. Cogger, G. R. Colli, A. de Silva, C. C. Eisemberg, J. Els, A. Fong G., T. D. Grant, R. A. Hitchmough, D. T. Iskandar, N. Kidera, M. Martins, S. Meiri, N. J. Mitchell, S. Molur, C. de C. Nogueira, J. C. Ortiz, J. Penner, A. G. J. Rhodin, G. A. Rivas, M.-O. Rödel, U. Roll, K. L. Sanders, G. Santos-Barrera, G. M. Shea, S. Spawls, B. L. Stuart, K. A. Tolley, J.-F. Trape, M. A. Vidal, P. Wagner, B. P. Wallace, Y. Xie, A global reptile assessment highlights shared conservation needs of tetrapods. Nature 605, 285–290 (2022). 35477765 10.1038/s41586-022-04664-7 PMC9095493

[141] L. J. Revell, phytools: An R package for phylogenetic comparative biology (and other things). Methods Ecol. Evol. 3, 217–223 (2012).

[142] L. si Tung Ho, C. Ané, A linear-time algorithm for gaussian and non-gaussian trait evolution models. Syst. Biol. 63, 397–408 (2014). 24500037 10.1093/sysbio/syu005

[143] J. A. Britnell, Y. Zhu, G. I. H. Kerley, S. Shultz, Ecological marginalization is widespread and increases extinction risk in mammals. Proc. Natl. Acad. Sci. U.S.A. 120, e2205315120 (2023). 36623195 10.1073/pnas.2205315120 PMC9934025

[144] IUCN, Guidelines for using the IUCN red list categories and criteria, version 16 (2024); www.iucnredlist.org/resources/redlistguidelines.

[145] R. P. Powers, W. Jetz, Global habitat loss and extinction risk of terrestrial vertebrates under future land-use-change scenarios. Nat. Clim. Chang. 9, 323–329 (2019).

[146] A. Purvis, J. L. Gittleman, G. Cowlishaw, G. M. Mace, Predicting extinction risk in declining species. Proc. R. Soc. B. Biol. Sci. 267, 1947–1952 (2000). 10.1098/rspb.2000.1234 PMC1690772 11075706

[147] L. Souviron-Priego, A. L. Márquez, N. Korbee, F. L. Figueroa, R. Real, Understanding the invasion of the macroalga *Rugulopteryx okamurae* (Ochrophyta) in the northern Alboran Sea through the use of biogeographic models. Sci. Total Environ. 955, 176851 (2024). 39396794 10.1016/j.scitotenv.2024.176851

[148] G. Horváth, L. Z. Garamszegi, G. Herczeg, Phylogenetic meta-analysis reveals system-specific behavioural type–behavioural predictability correlations. R. Soc. Open Sci. 10, 230303 (2023). 37680498 10.1098/rsos.230303 PMC10480700

[149] C. Spearman, The proof and measurement of association between two things. Am. J. Psychol. 15, 72 (1904).

[150] M. R. E. Symonds, A. Moussalli, A brief guide to model selection, multimodel inference and model averaging in behavioural ecology using Akaike’s information criterion. Behav. Ecol. Sociobiol. 65, 13–21 (2011).

[151] K. P. Burnham, D. R. Anderson, Eds., *Model Selection and Multimodel Inference* (Springer, 2004).

[152] D. B. Rubin, *Multiple Imputation for Nonresponse in Surveys* (Wiley, ed. 1, 1987).

[153] K. Bartoń, MuMIn: Multi-model inference, version 1.48.11 (2025); https://cran.r-project.org/web/packages/MuMIn/index.html.

[154] A. R. Ives, T. Garland Jr., Phylogenetic logistic regression for binary dependent variables. Syst. Biol. 59, 9–26 (2010). 20525617 10.1093/sysbio/syp074

[155] T. B. Atwood, S. A. Valentine, E. Hammill, D. J. McCauley, E. M. P. Madin, K. H. Beard, W. D. Pearse, Herbivores at the highest risk of extinction among mammals, birds, and reptiles. Sci. Adv. 6, eabb8458 (2020). 32923612 10.1126/sciadv.abb8458 PMC7457337

[156] R. J. Hijmans, M. Barbosa, R. Bivand, A. Brown, M. Chirico, E. Cordano, K. Dyba, E. Pebesma, B. Rowlingson, M. D. Sumner, terra: Spatial data analysis, version 1.8-93 (2026); https://cran.r-project.org/web/packages/terra/index.html.

[157] G. Kalinkat, S. C. Jähnig, J. M. Jeschke, Exceptional body size–extinction risk relations shed new light on the freshwater biodiversity crisis. Proc. Natl. Acad. Sci. U.S.A. 114, E10263–E10264 (2017). 29122937 10.1073/pnas.1717087114 PMC5715793

[158] P. M. Lucas, M. Di Marco, V. Cazalis, J. Luedtke, K. Neam, M. H. Brown, P. F. Langhammer, G. Mancini, L. Santini, Using comparative extinction risk analysis to prioritize the IUCN Red List reassessments of amphibians. Conserv. Biol. 38, e14316 (2024). 38946355 10.1111/cobi.14316 PMC11589027

[159] S. Ducatez, R. Tingley, R. Shine, Using species co-occurrence patterns to quantify relative habitat breadth in terrestrial vertebrates. Ecosphere 5, 1–12 (2014).

[160] G. Murali, R. Gumbs, S. Meiri, U. Roll, Global determinants and conservation of evolutionary and geographic rarity in land vertebrates. Sci. Adv. 7, eabe5582 (2021). 34644103 10.1126/sciadv.abe5582 PMC8514094

[161] M. R. Cardoso, A. M. Bastidas-Urrutia, K. Frac, C. Hof, H. Kreft, J. Albrecht, K. Böhning-Gaese, S. A. Fritz, Urbanisation is related to the prevalence of threatened species on islands across the globe. Glob. Ecol. Biogeogr. 34, e70125 (2025).

[162] T. S. Doherty, A. S. Glen, D. G. Nimmo, E. G. Ritchie, C. R. Dickman, Invasive predators and global biodiversity loss. Proc. Natl. Acad. Sci. U.S.A. 113, 11261–11265 (2016). 27638204 10.1073/pnas.1602480113 PMC5056110

