# Supplementary material for: Global patterns and drivers of extinction risk in island endemic terrestrial vertebrates
Source: Sci Adv. 2026 Aug 5;12(32):eaef9781. doi: 10.1126/sciadv.aef9781 (PMC13440397; doi:10.1126/sciadv.aef9781)
Supplement: Supplementary file 1 [file sciadv.aef9781_sm.pdf]

Science Advances
AAAS

Supplementary Materials for

Global patterns and drivers of extinction risk in island endemic terrestrial vertebrates

Yuyang Zhang *et al.*

Corresponding author: Yanping Wang, wangyanping@njnu.edu.cn

Sci. Adv. **12**, eaef9781 (2026)
DOI: 10.1126/sciadv.aef9781

The PDF file includes:

Supplementary Methods
Legends for data S1 to S11
Tables S1 to S6
Figs. S1 to S5
References

Other Supplementary Material for this manuscript includes the following:

Data S1 to S11

Supplementary Methods

Taxonomic reconciliation and name standardization

Species names and synonymy were cross-checked against multiple authoritative databases: BirdLife International (www.birdlife.org) for birds, the GBIF Backbone Taxonomy (www.gbif.org) for mammals, AmphibiaWeb (amphibiaweb.org) for amphibians, and The Reptile Database (reptile-database.org) for reptiles. Subspecies recognized in these databases were excluded, and synonyms were merged following the most widely accepted nomenclatural consensus. For taxonomically uncertain groups or species complexes, we adopted the taxonomy currently followed by the IUCN Red List (version 2024-1) to maintain comparability. Species names were further verified for spelling consistency and synonymy using the rl_synonymsfunction from the R package rredlist (v0.7.1), complemented by manual inspection of remaining inconsistencies.

Data Compilation of Ecological, Environmental, and Anthropogenic variables

S1. Compilation of species-level variables

We assembled a comprehensive dataset of species-level variables for all non-Data Deficient terrestrial vertebrates, encompassing ecological and life-history traits, geographic and environmental attributes, and anthropogenic or historical pressures. The baseline source for biological and ecological traits was the pre-imputation version of the TetrapodTraits database (*116*). To improve trait coverage and ensure reliance on published, non-imputed data, we supplemented TetrapodTraits with additional empirically derived datasets (*117–122*). All supplementary datasets are literature-based and contain original, non-imputed measurements. We retained the body length values that had been previously imputed by Moura et al. (*116*). Because these sources differ in coding schemes and measurement units, we harmonized all raw trait values following the standards defined in Table S1.

Taxonomic inconsistencies between our species checklist and database entries were handled systematically. When two names referred to the same biological entity (synonyms, previous names, orthographic variants), we directly adopted the trait values provided in the original dataset. In cases where a newly recognized species (species A) had historically been recorded under another name or treated as a subspecies or species complex member (species B), we retained the updated taxonomy but used species B's morphological and ecological traits as the best available approximation for species A. Only environmental variables were recalculated for such newly recognized species, while morphological and ecological traits were not removed or subjected to imputation in these cases.

S2. Ecological and life-history traits

Body size was represented by body mass for birds, mammals, and reptiles, and by body length for amphibians. Ecological traits included activity pattern, vertical habitat use, trophic level, and habitat breadth, calculated as the number of IUCN-defined major habitat types associated with each species. Amphibians were additionally assigned a developmental mode. This set of traits was selected because they capture fundamental axes of ecological variation that are both comparable across vertebrate classes and have established links to extinction risk in island species (*24*, *27*, *155*). Body mass was used for birds, mammals, and reptiles as it is a standardized measure of size with direct ties to metabolic and life-history constraints.

Body length was used for amphibians due to its broader availability and consistency in trait databases for this group (*85*). All supplements, except for amphibian body length, were based on original or measured data rather than secondary interpolation results. The retention of amphibian body length data previously imputed by Moura et al. (*116*) was based on considerations of data completeness and imputation performance, while ensuring consistency within the taxonomic group.

For discrete ecological strategies, ordinal categories were arranged following biologically meaningful progressions to maintain consistent directionality for modelling. For instance, activity pattern was coded as 0 (nocturnal), 1 (diurnal), and 2 (cathemeral/crepuscular) to capture increasing behavioral flexibility following Davidson et al. (*132*). All trait values used were taken directly from literature-based trait datasets or transferred from closely related species in taxonomic revision cases, as described above.

S3. Geographic and environmental attributes

Geographic range size was quantified as the number of 1km×1km grid cells intersecting each species' IUCN distribution polygon. For species lacking range polygons in the literature, we applied the following hierarchical approach:

(1) we searched biogeographical information databases for expert-defined distributions, with mammals and amphibians from the IUCN (www.iucnredlist.org), birds from BirdLife International (www.datazone.birdlife.org), and reptiles from the GARD 1.7 dataset (*110*);

(2) if no polygon was available, we downloaded GBIF occurrence points and constructed a 10 km radius buffer around all points to approximate the range (*116*);

(3) for species completely lacking digital range data, we consulted the primary literature cited in the IUCN Red List species assessment accounts and constructed distribution polygons based on province- or region-level occurrence information reported in those sources.

The supplemented geographic and environmental attributes were derived from standardized global datasets and represent arithmetic means extracted from rasterized versions of each species' distribution polygon. Elevation values were obtained from the EarthEnv digital elevation model (*123*), and climatic variables—including mean annual temperature, annual precipitation, and the seasonality of temperature and precipitation—were sourced from CHELSA v2.1 (*124*). All spatial extractions were performed in R using the *terra* package (*156*). A subset of environmental values originally included in TetrapodTraits (*116*) was recalculated after data cleaning to avoid obvious geographic or climatic inconsistencies in the raw dataset.

S4. Anthropogenic pressures

Anthropogenic land-use intensity was quantified using the Global 100 m Terrestrial Human Footprint (HFP-100) v1.2 dataset to improve coverage in remote areas (*126*), supplemented with the Global Annual Terrestrial Human Footprint (2000–2018) dataset (*125*). For each species, mean human footprint intensity was computed across its native range.

Invasive species pressure was quantified using the residuals from a log–log regression between invasive species richness and geographic range size (both log1p-transformed), representing deviation from expected invasion levels given spatial extent. Invasive range maps represent non-native, invaded ranges—not natural distributions. For birds and mammals,

invaded polygons were obtained directly from published global databases (*127*, *128*). For reptiles and amphibians, invaded ranges were generated by applying 10 km radius buffers around known invasion occurrence points (*64*).

To address potential bias associated with discovery history (*31*, *131*), we recorded the year of formal species description and calculated the elapsed time since description as 2024 minus description year. This metric served as a proxy for taxonomic discovery bias across vertebrate groups.

Correlates of Extinction Risk

Pairwise correlations among candidate predictor variables were assessed using Spearman's rank correlation coefficient (ρ). Correlation analyses were conducted separately within each of the 100 paired datasets following identical preprocessing procedures. Because correlation coefficients are bounded between −1 and 1 and are not additive, Fisher's z-transformation was applied to each Spearman correlation coefficient prior to aggregation (*148*):

$$z=\frac{1}{2}\ln\left(\frac{1+\rho}{1-\rho}\right)$$

Fisher's z values were then averaged across datasets for each variable pair, and the mean z values were back-transformed to correlation coefficients using the inverse Fisher transformation:

$$\rho=\frac{e^{2z}-1}{e^{2z}+1}$$

The resulting averaged correlation matrices were used to identify strongly correlated predictor pairs ($|\rho| > 0.7$) (*64*). For each correlated pair, variable selection was guided by explanatory power estimated from univariate phylogenetic regression models. Specifically, the marginal coefficient of determination (R^2) was calculated for each variable, and the variable with the higher R^2 was preferentially retained (*147*). When R^2 values were similar, biological interpretability and data availability were used as additional selection criteria.

Supplementary Data

Data S1. Taxonomic filtering and dataset construction for 32,214 non-Data Deficient terrestrial vertebrate species.

Full data are available as Supplementary Data 1 (separate file), which contains the following columns:

iucn_assessment_id and iucn_taxon_id: IUCN Red List identifiers.

scientific_name: Accepted binomial name after taxonomic reconciliation.

iucn_category: IUCN Red List category (2024-1 assessment). Species listed as Extinct (EX) or Extinct in the Wild (EW) were retained and treated as extinct.

excluded_dd: Logical flag indicating exclusion of Data Deficient (DD) species during the first filtering step.

synonym_or_subspecies and synonym_type: Records of resolved synonyms or excluded subspecies.

marine_system, freshwater_system, terrestrial_system: Habitat system flags used to exclude strictly marine taxa.

excluded_taxonomic_issue and excluded_non_terrestrial: Logical flags for subsequent filtering steps.

retained_in_final_dataset: Indicates retention after all taxonomic and habitat filters (n = 31,892 species).

missing_core_traits: Logical flag for species lacking any ecological or life-history trait beyond major habitat type.

included_in_analysis: Final 30,711 species with sufficient trait data that entered phylogenetic imputation and comparative modelling.

Data S2. Summary of non-DD Terrestrial vertebrate species

This dataset contains the core taxonomic, biogeographic, and conservation assessment attributes for 31,865 non-Data Deficient (non-DD) terrestrial vertebrate species included in this study. For each species, we report the scientific name, unique IUCN assessment identifier, taxonomic classification (class, order, and family), endemicity category, IUCN Red List category, assessment criteria, criteria version, and population trend as documented in the IUCN Red List database. Endemicity is coded as three categories: 1 = island endemic, 2 = continental endemic, and 3 = dual-range species occurring on both islands and continents. Red List categories follow the IUCN classification system (version 3.1), and population trends are reported as assessed by the IUCN (version 2024-1). Full data are available as Supplementary Data 2 (separate file).

Data S3. Raw data for the 30,711 analyzed tetrapod species before any imputation

This dataset contains the original, unimputed values compiled and harmonized from multiple sources for all variables as they were before taxonomic imputation, log-transformation, or standardization. Missing values are marked as NA (not available). Full data are available as Supplementary Data 3 (separate file).

Data S4. Species richness of island endemic terrestrial vertebrates by archipelago and taxonomic class

This dataset contains 11 sheets: (1) Archipelago label and name correspondence table; (2) Overall endemic richness (all tetrapods); (3) high-risk endemic richness (all tetrapods); (4–7) endemic richness for birds/mammals/reptiles/amphibians; (8–11) high-risk endemic richness for each class. Archipelago labels match those shown in Fig. 3. Full data are available as Supplementary Data 4 (separate file).

Data S5. Taxonomic clustering of extinction risk among island endemic terrestrial vertebrate families

This dataset contains four worksheets, one for each vertebrate class.

Aves: 154 families (85 with ≥5 island endemic species tested, 69 with $n < 5$ reported descriptively), comprising all 2,661 island endemic bird species

Mammalia: 75 families (40 with ≥5 island endemic species tested, 35 with $n < 5$ reported descriptively), comprising all 1,081 island endemic mammal species

Amphibia: 36 families (17 with ≥5 island endemic species tested, 19 with $n < 5$ reported descriptively), comprising all 1,462 island endemic amphibian species

Reptilia: 62 families (38 with ≥5 island endemic species tested, 24 with $n < 5$ reported descriptively), comprising all 2,365 island endemic reptile species

For each family, the worksheets report total island endemic species (n_species) and number of high-risk species (Extinct + Critically Endangered + Endangered + Vulnerable), observed and class-level expected high-risk proportions, exact binomial test two-sided P-value, Clopper–Pearson 95% confidence interval on the observed proportion, Dunn–Šidák-adjusted α and corresponding adjusted confidence level (applied separately within each class to control family-wise error rate), significance after correction (corrected P < adjusted α), and classification as "Higher ($p < 0.05$)"/"Lower ($p < 0.05$)"/"Not significant" for tested families ($n \geq 5$); descriptive categories for small families ($n < 5$). The background high-risk proportion for each class is calculated from all island endemic species in that class (including small families), ensuring an unbiased reference. Full data are available as Supplementary Data 5 (separate file).

Data S6. Contingency table analyses of the association between major IUCN threat types and Red List categories in threatened non-Data Deficient terrestrial vertebrates, separated by taxonomic class and endemicity group

This Excel workbook contains 14 sheets with the following content:

Sheet 1: Summary of Pearson's χ^2 tests of independence for each of the eight taxonomic class × endemism combinations (island endemic, continental endemic, dual-range). Columns include: taxonomic class, endemism status, total χ^2 statistic, degrees of freedom, raw p-value, and significance level (***$p < 0.001$, **$p < 0.01$, *$p < 0.05$, ns = not significant).

Sheets 2–13: Detailed cell-by-cell results for each class × endemism combination (e.g., "Aves_Island_Endemic", "Aves_Continental_Endemic", "Aves_Dual-Range"). Columns report, for every combination of the 11 major IUCN threat types (threat1 to threat11) and the four Red List categories (VU, EN, CR, EX): observed count, expected count under the null hypothesis of independence, standardized Pearson residual ((observed - expected)/√expected), χ^2 contribution ((observed - expected)²/expected), raw cell p-value, FDR-adjusted p-value

(Benjamini–Hochberg correction within each group), and significance of the cell-level χ^2 contribution based on adjusted p-value (***p_adj < 0.001, **p_adj < 0.01, *p_adj < 0.05, blank otherwise).
Sheet 14: IUCN threats classification scheme (version 3.1), Threat 12 (Other threat) and Unknown were excluded.
Full data are available as Supplementary Data 6 (separate file).

Data S7. Species-level correlation matrices for univariate predictor variables across vertebrate classes and subgroups

This file contains 32 sheets of correlation matrices showing pairwise Spearman correlation coefficients among all univariate predictor variables used in the extinction risk analyses. Correlations were calculated for four vertebrate classes (birds, mammals, amphibians, reptiles) and multiple species subgroups defined by endemicity and IUCN criteria, respectively. Notably, "criteria2" sheets correspond to subsets of species within each class and endemicity type for which species classified as threatened under IUCN Criteria B or D2 were excluded. For each dataset, Spearman correlation coefficients were first Fisher z-transformed, averaged across 100 paired datasets, and then inverse-transformed to obtain mean correlation values. Each sheet corresponds to one class-subgroup combination, with variable names mapped to standardized analysis identifiers. Values range from –1 to 1, with positive values indicating positive associations between variables. Full data are available as Supplementary Data 7 (separate file).

Data S8. Univariate phylogenetic linear model results implemented in phylolm

This dataset contains the results of univariate phylogenetic linear models (PLMs) implemented using the phylolm framework, evaluating the association between individual predictor variables and extinction risk (ordinal IUCN categories, LC = 0 to EX = 5) across global terrestrial vertebrates. For each predictor, effect estimates, confidence intervals, and statistical significance are reported separately for each vertebrate group and endemicity category. These univariate analyses were used to characterize the marginal effects of species traits and external variables prior to multivariable model averaging. Full data are available as Supplementary Data 8 (separate file).

Data S9. Model-averaged phylogenetic linear model results implemented in phylolm

This dataset summarizes model-averaged parameter estimates derived from phylogenetic linear models (PLMs) implemented using the phylolm framework fitted to 100 paired datasets. Estimates incorporate both within-dataset and between-dataset variance, and are reported for each vertebrate group, endemicity category, and extinction risk criteria subset. Full data are available as Supplementary Data 9 (separate file). This dataset provides the source data for the synthesis of extinction risk correlates shown in Fig. 5.

Data S10. Univariate phylogenetic logistic regression model results implemented in phyloglm

This dataset contains the results of univariate phylogenetic logistic regression models implemented using the phyloglm framework, evaluating the association between individual

predictor variables and extinction risk across global terrestrial vertebrates. Extinction risk is treated as a binary response based on IUCN red list category. For each predictor variable, model outputs include effect estimates, standard errors, z-values, p-values, and 95% confidence intervals. In addition, phylogenetically corrected model parameters, including the phylogenetic signal parameter where applicable, are reported. Results are provided separately for each vertebrate class, endemicity category, and extinction risk criterion subset. Full data are available as Supplementary Data 10 (separate file).

Data S11. Model-averaged phylogenetic logistic regression results implemented in phyloglm

This dataset summarizes model-averaged parameter estimates derived from phylogenetic logistic regression models implemented using the phyloglm framework fitted across multiple candidate model sets. Estimates incorporate both within-dataset and between-dataset variance, and are reported for each vertebrate class, endemicity category and extinction risk criteria subset. For each predictor variable, the dataset provides model-averaged effect estimates, standard errors, z-values, p-values, and model importance values, together with odds ratios and their corresponding 95% confidence intervals. Full data are available as Supplementary Data 11 (separate file).

Supplementary Tables

Table S1. Description of predictors used in the extinction-risk correlation and regression analyses

Raw_Variable_Name_in_Dataset	Description_and_Unit	Preprocessing_before_Model_Averaging	References
BodyMass	Adult body mass (g) – Aves, Mammalia, Reptilia	log(BodyMass_g) → scale()	(*25*, *27*)
BodyLength	Maximum body length (mm) – Amphibia only	log(BodyLength) → scale()	(*157*, *85*)
Nocturnality	Activity pattern: 0=nocturnal, 1=diurnal, 2=cathemeral/crepuscular	Recoded to 0/1/2, used as-is	(*132*)
Verticality	Vertical stratum preference: 0=fossorial, 1=semifossorial, 2=terrestrial/aquatic, 3=semi-arboreal, 4=arboreal/aerial	Recoded to 0–4, used as-is	(*24*)
TrophicLevel	Trophic level: 0=omnivore, 1=herbivore, 2=carnivore	Recoded to 0/1/2, used as-is	(*76*)
GrowthType	Larval development mode (Amphibia): 1=indirect, 2=direct, 3=viviparous	Converted to numeric, used as-is	(*158*)
MajorHabitatSum	Habitat breadth (number of IUCN major habitat types, 1–13)	Converted to numeric, used as-is	(*159*)
RangeSize	Geographic range size (number of occupied 1 km × 1 km grid cells)	log(rangesize + 1) → scale()	(*36*)
Latitude	Range centroid latitude (decimal degrees)	abs(Latitude) → scale()	(*8*, *63*)
Elevation	Mean elevation within range (m)	scale()	(*35*, *37*)
AnnuMeanTemp	Annual mean temperature within range (°C)	scale()	(*160*)
AnnuPrecip	Annual mean precipitation within range (mm)	scale()	(*160*)
TempSeasonality	Temperature seasonality (SD × 100)	scale()	(*160*)
PrecipSeasonality	Precipitation seasonality (CV)	scale()	(*160*)
HumanFootprint	Human Footprint index (0–50)	NA→0; mean → ÷1000 → scale()	(*161*)
InvasiveSpeciesResidual	Residual invasive species richness after controlling for range size, Residuals of lm(log(invasive richness + 1) ~ log(rangesize + 1))	Used directly as residuals	(*162*)
DescriptionTime	Years since first description (2024 − YearOfDescription)	log() → scale()	(*131*)
EndemicType	Endemic status (1=island endemic,2=continent endemic, 3=dual-range)	Used only for subsetting	(*11*)
Criteria	IUCN listing criteria (1=include B/D2, 2= exclude B/D2)	Used only for subsetting	(*39*)

Table S2. Extinction risk status of island endemic and non-endemic terrestrial vertebrate species by taxonomic class

Numbers and proportions of species in each IUCN Red List category for terrestrial vertebrates across three endemism classes (island endemic, continental endemic, and dual-range species), broken down by the four major taxonomic classes (Aves, Mammalia, Reptilia, Amphibia) and summarised for all classes combined. Proportions are calculated relative to the total number of non-Data Deficient species within each class and endemism group.

Class_Name	Endemic_Type	Threat_Level	Count	Total	Proportion
Aves	Island endemic	EX	141	2661	0.0530
Aves	Island endemic	CR	128	2661	0.0481
Aves	Island endemic	EN	168	2661	0.0631
Aves	Island endemic	VU	294	2661	0.1105
Aves	Island endemic	NT	362	2661	0.1360
Aves	Island endemic	LC	1568	2661	0.5893
Aves	Continental endemic	EX	5	3159	0.0016
Aves	Continental endemic	CR	55	3159	0.0174
Aves	Continental endemic	EN	142	3159	0.0450
Aves	Continental endemic	VU	221	3159	0.0700
Aves	Continental endemic	NT	271	3159	0.0858
Aves	Continental endemic	LC	2465	3159	0.7803
Aves	Dual-range	EX	6	5286	0.0011
Aves	Dual-range	CR	42	5286	0.0079
Aves	Dual-range	EN	87	5286	0.0165
Aves	Dual-range	VU	193	5286	0.0365
Aves	Dual-range	NT	300	5286	0.0568
Aves	Dual-range	LC	4658	5286	0.8812
Mammalia	Island endemic	EX	48	1081	0.0444
Mammalia	Island endemic	CR	114	1081	0.1055
Mammalia	Island endemic	EN	220	1081	0.2035
Mammalia	Island endemic	VU	188	1081	0.1739
Mammalia	Island endemic	NT	64	1081	0.0592
Mammalia	Island endemic	LC	447	1081	0.4135
Mammalia	Continental endemic	EX	23	1896	0.0121
Mammalia	Continental endemic	CR	95	1896	0.0501
Mammalia	Continental endemic	EN	219	1896	0.1155
Mammalia	Continental endemic	VU	206	1896	0.1086
Mammalia	Continental endemic	NT	150	1896	0.0791
Mammalia	Continental endemic	LC	1203	1896	0.6345
Mammalia	Dual-range	EX	13	2073	0.0063
Mammalia	Dual-range	CR	21	2073	0.0101
Mammalia	Dual-range	EN	94	2073	0.0453
Mammalia	Dual-range	VU	149	2073	0.0719

Mammalia	Dual-range	NT	154	2073	0.0743
Mammalia	Dual-range	LC	1642	2073	0.7921
Amphibia	Island endemic	EX	16	1462	0.0109
Amphibia	Island endemic	CR	141	1462	0.0964
Amphibia	Island endemic	EN	274	1462	0.1874
Amphibia	Island endemic	VU	177	1462	0.1211
Amphibia	Island endemic	NT	108	1462	0.0739
Amphibia	Island endemic	LC	746	1462	0.5103
Amphibia	Continental endemic	EX	20	4207	0.0048
Amphibia	Continental endemic	CR	623	4207	0.1481
Amphibia	Continental endemic	EN	933	4207	0.2218
Amphibia	Continental endemic	VU	582	4207	0.1383
Amphibia	Continental endemic	NT	317	4207	0.0754
Amphibia	Continental endemic	LC	1732	4207	0.4117
Amphibia	Dual-range	EX	3	1350	0.0022
Amphibia	Dual-range	CR	11	1350	0.0081
Amphibia	Dual-range	EN	31	1350	0.0230
Amphibia	Dual-range	VU	41	1350	0.0304
Amphibia	Dual-range	NT	26	1350	0.0193
Amphibia	Dual-range	LC	1238	1350	0.9170
Reptilia	Island endemic	EX	31	2365	0.0131
Reptilia	Island endemic	CR	239	2365	0.1011
Reptilia	Island endemic	EN	368	2365	0.1556
Reptilia	Island endemic	VU	263	2365	0.1112
Reptilia	Island endemic	NT	240	2365	0.1015
Reptilia	Island endemic	LC	1224	2365	0.5175
Reptilia	Continental endemic	EX	2	3769	0.0005
Reptilia	Continental endemic	CR	149	3769	0.0395
Reptilia	Continental endemic	EN	358	3769	0.0950
Reptilia	Continental endemic	VU	280	3769	0.0743
Reptilia	Continental endemic	NT	251	3769	0.0666
Reptilia	Continental endemic	LC	2729	3769	0.7241
Reptilia	Dual-range	CR	38	2556	0.0149
Reptilia	Dual-range	EN	56	2556	0.0219
Reptilia	Dual-range	VU	75	2556	0.0293
Reptilia	Dual-range	NT	71	2556	0.0278
Reptilia	Dual-range	LC	2316	2556	0.9061
All	Island endemic	EX	236	7569	0.0312
All	Island endemic	CR	622	7569	0.0822
All	Island endemic	EN	1030	7569	0.1361
All	Island endemic	VU	922	7569	0.1218
All	Island endemic	NT	774	7569	0.1023
All	Island endemic	LC	3985	7569	0.5265
All	Continental endemic	EX	50	13031	0.0038

All	Continental endemic	CR	922	13031	0.0708
All	Continental endemic	EN	1652	13031	0.1268
All	Continental endemic	VU	1289	13031	0.0989
All	Continental endemic	NT	989	13031	0.0759
All	Continental endemic	LC	8129	13031	0.6238
All	Dual-range	EX	22	11265	0.0020
All	Dual-range	CR	112	11265	0.0099
All	Dual-range	EN	268	11265	0.0238
All	Dual-range	VU	458	11265	0.0407
All	Dual-range	NT	551	11265	0.0489
All	Dual-range	LC	9854	11265	0.8747

Table S3. Chi-square tests comparing extinction risk between island endemic and non-endemic terrestrial vertebrates across taxonomic classes

Results of 2 × 2 Pearson chi-square tests with Yates' continuity correction comparing the proportion of threatened species between island endemic and non-endemic terrestrial vertebrates, both for the pooled dataset and for each major vertebrate class (birds, mammals, amphibians, and reptiles). High-risk species are defined as those listed as Extinct, Critically Endangered, Endangered, or Vulnerable under the IUCN Red List. Prop1 and Prop2 represent the proportions of high-risk species in island endemic and non-endemic groups listed in the comparison, and Prop_Ratio denotes the ratio of these two proportions. Raw species counts underlying each test are provided in Supplementary Data S2.

ClassName	Chi_Sq_Stat	P_Value	Prop1	Prop2	Prop_Ratio
Overall	971.4250	2.92E-213	0.3713	0.1965	1.8898
Aves	602.3405	5.18E-133	0.2747	0.0889	3.0891
Mammalia	436.3885	6.61E-97	0.5273	0.2066	2.5522
Amphibia	0.6481	0.420799954	0.4159	0.4038	1.0298
Reptilia	537.8377	5.57E-119	0.3810	0.1515	2.5153

Table S4. Chi-square tests of differences in extinction risk across three endemic types

Results of chi-square tests comparing the proportion of threatened species among different endemism categories across four major classes of terrestrial vertebrates (birds, mammals, amphibians, and reptiles). For each class, an overall Pearson chi-square test was first conducted to assess differences among the three endemism groups — island endemic (IE), continental endemic (CE), and dual-range species (DR). This was followed by pairwise 2 × 2 chi-square tests with Yates' continuity correction to compare high-risk proportions between specific endemism categories. For pairwise comparisons, Prop1 and Prop2 represent the proportions of high-risk species in the first and second groups listed in the comparison, and Prop_Ratio denotes the ratio of these two proportions. Proportions were calculated based on all non–Data Deficient species, with high-risk species defined as those classified as Extinct, Critically Endangered, Endangered, or Vulnerable under the IUCN Red List. Raw species counts underlying each test are provided in Supplementary Data S2.

ClassName	Comparison	Chi_Sq_Stat	Df	P_Value	Prop1	Prop2	Prop_Ratio
Overall	All 3 groups	2643.2670	2	0	NA	NA	NA
Overall	IE vs CE	109.3607	1	1.35E-25	0.3713	0.3003	1.2363
Overall	IE vs DR	2507.7351	1	0	0.3713	0.0763	4.8630
Overall	CE vs DR	1918.0195	1	0	0.3003	0.0763	3.9334
Aves	All 3 groups	692.2279	2	4.84E-151	NA	NA	NA
Aves	IE vs CE	179.2540	1	7.05E-41	0.2747	0.1339	2.0515
Aves	IE vs DR	691.1865	1	2.47E-152	0.2747	0.0621	4.4272
Aves	CE vs DR	125.1138	1	4.81E-29	0.1339	0.0621	2.1580
Mammalia	All 3 groups	553.8503	2	5.41E-121	NA	NA	NA
Mammalia	IE vs CE	169.6456	1	8.84E-39	0.5273	0.2864	1.8411
Mammalia	IE vs DR	558.5462	1	1.74E-123	0.5273	0.1336	3.9461
Mammalia	CE vs DR	140.0654	1	2.58E-32	0.2864	0.1336	2.1433
Amphibia	All 3 groups	855.8057	2	1.46E-186	NA	NA	NA
Amphibia	IE vs CE	40.5422	1	1.92E-10	0.4159	0.5130	0.8107
Amphibia	IE vs DR	466.3850	1	1.96E-103	0.4159	0.0637	6.5282
Amphibia	CE vs DR	854.9366	1	6.14E-188	0.5130	0.0637	8.0522
Reptilia	All 3 groups	724.9873	2	3.72E-158	NA	NA	NA
Reptilia	IE vs CE	213.5992	1	2.25E-48	0.3810	0.2093	1.8199
Reptilia	IE vs DR	713.8085	1	2.97E-157	0.3810	0.0661	5.7619
Reptilia	CE vs DR	241.9731	1	1.46E-54	0.2093	0.0661	3.1661

Table S5. Population trends of (a) all island endemic terrestrial vertebrates by taxonomic class; (b) threatened island endemic terrestrial vertebrates by taxonomic class.

Percentages are calculated relative to the total number of species within each taxonomic class in the respective subset (a: all island endemics; b: threatened island endemics only). Extinct (EX) and Data Deficient (DD) species are excluded from both panels. Raw data are provided in Supplementary Data 2.

**(a)**			
**Taxonomic Group**	**Population Trend**	**Species Count**	**Percentage (%)**
Aves	Increasing	86	3.2319
Aves	Stable	950	35.7009
Aves	Decreasing	1270	47.7264
Aves	Unknown	355	13.3408
Mammalia	Increasing	7	0.6475
Mammalia	Stable	190	17.5763
Mammalia	Decreasing	541	50.0463
Mammalia	Unknown	343	31.7299
Amphibia	Increasing	2	0.1368
Amphibia	Stable	426	29.1382
Amphibia	Decreasing	826	56.4979
Amphibia	Unknown	208	14.2271

Reptilia	Increasing	31	1.3108
Reptilia	Stable	681	28.7949
Reptilia	Decreasing	609	25.7505
Reptilia	Unknown	1044	44.1438

(b)

**Taxonomic Group**	**Population Trend**	**Species Count**	**Percentage (%)**
Aves	Increasing	40	6.7797
Aves	Stable	89	15.0847
Aves	Decreasing	432	73.2203
Aves	Unknown	29	4.9153
Mammalia	Increasing	3	0.5747
Mammalia	Stable	23	4.4061
Mammalia	Decreasing	429	82.1839
Mammalia	Unknown	67	12.8352
Amphibia	Increasing	2	0.3378
Amphibia	Stable	28	4.7297
Amphibia	Decreasing	529	89.3581
Amphibia	Unknown	33	5.5743
Reptilia	Increasing	20	2.2989
Reptilia	Stable	46	5.2874
Reptilia	Decreasing	457	52.5287
Reptilia	Unknown	347	39.8851

Table S6. Island endemism and extinction risk in 20 selected vertebrate orders representing the highest proportions of island endemics

High-risk categories include species classified as Extinct (EX), Critically Endangered (CR), Endangered (EN), or Vulnerable (VU) on the IUCN Red List, while Low-risk categories include Near Threatened (NT) and Least Concern (LC). The 20 orders comprise the top seven orders in Aves (n = 7), top seven orders in Mammalia (n = 7), top three orders in Amphibia (n = 3), and top three orders in Reptilia (n = 3).

Order_Name	Class_Name	Total_Species_Count	Island endemics_Count	Endemic_Ratio	High_Risk_Count	High_Risk_Ratio	Endemic_High_Risk_Count	Endemic_High_Risk_Ratio
Columbiformes	Aves	368	217	0.5897	81	0.2201	68	0.3134
Psittaciformes	Aves	421	166	0.3943	125	0.2969	63	0.3795
Passeriformes	Aves	6673	1528	0.2290	668	0.1001	308	0.2016
Galliformes	Aves	307	48	0.1564	75	0.2443	18	0.3750
Caprimulgiformes	Aves	595	84	0.1412	53	0.0891	12	0.1429
Charadriiformes	Aves	382	47	0.1230	57	0.1492	23	0.4894
Piciformes	Aves	483	42	0.0870	21	0.0435	9	0.2143
Diprotodontia	Mammalia	147	60	0.4082	60	0.4082	31	0.5167
Primates	Mammalia	510	172	0.3373	350	0.6863	166	0.9651
Chiroptera	Mammalia	1097	304	0.2771	230	0.2097	138	0.4539
Eulipotyphla	Mammalia	390	67	0.1718	83	0.2128	32	0.4776
Rodentia	Mammalia	1967	336	0.1708	364	0.1851	136	0.4048
Carnivora	Mammalia	289	33	0.1142	84	0.2907	20	0.6061
Artiodactyla	Mammalia	228	25	0.1096	107	0.4693	18	0.7200
Anura	Amphibia	6238	1380	0.2212	2434	0.3902	557	0.4036
Gymnophiona	Amphibia	111	18	0.1622	19	0.1712	6	0.3333
Caudata	Amphibia	670	64	0.0955	399	0.5955	45	0.7031
Squamata	Reptilia	8411	2318	0.2756	1670	0.1985	862	0.3719

Testudines	Reptilia	258	43	0.1667	178	0.6899	37	0.8605
Crocodylia	Reptilia	20	3	0.1500	11	0.5500	2	0.6667

Supplementary Figures

Fig. S1. Phylogenetic clustering of extinction risk across families of island endemic birds (Aves)

The proportion of high-risk species across families of island endemic birds. Only families with at least five island endemic species (n ≥ 5) were statistically tested. Points represent the observed proportion of high-risk species (Extinct, Critically Endangered, Endangered, and Vulnerable combined) within each family, and horizontal error bars indicate confidence intervals derived from exact binomial tests using the Clopper–Pearson method. The dashed line denotes the class-level background extinction risk proportion (27.47%), calculated from all island endemic bird species. Significance was assessed using two-sided exact binomial tests with Dunn–Šidák correction applied within the class. Red points indicate families with significantly higher proportions than the background, blue points indicate significantly lower proportions, and grey points indicate non-significant results. Point size is proportional to the number of island endemic species in each family. Triangles represent families with 5–9 species, and circles represent families with 10 or more species.

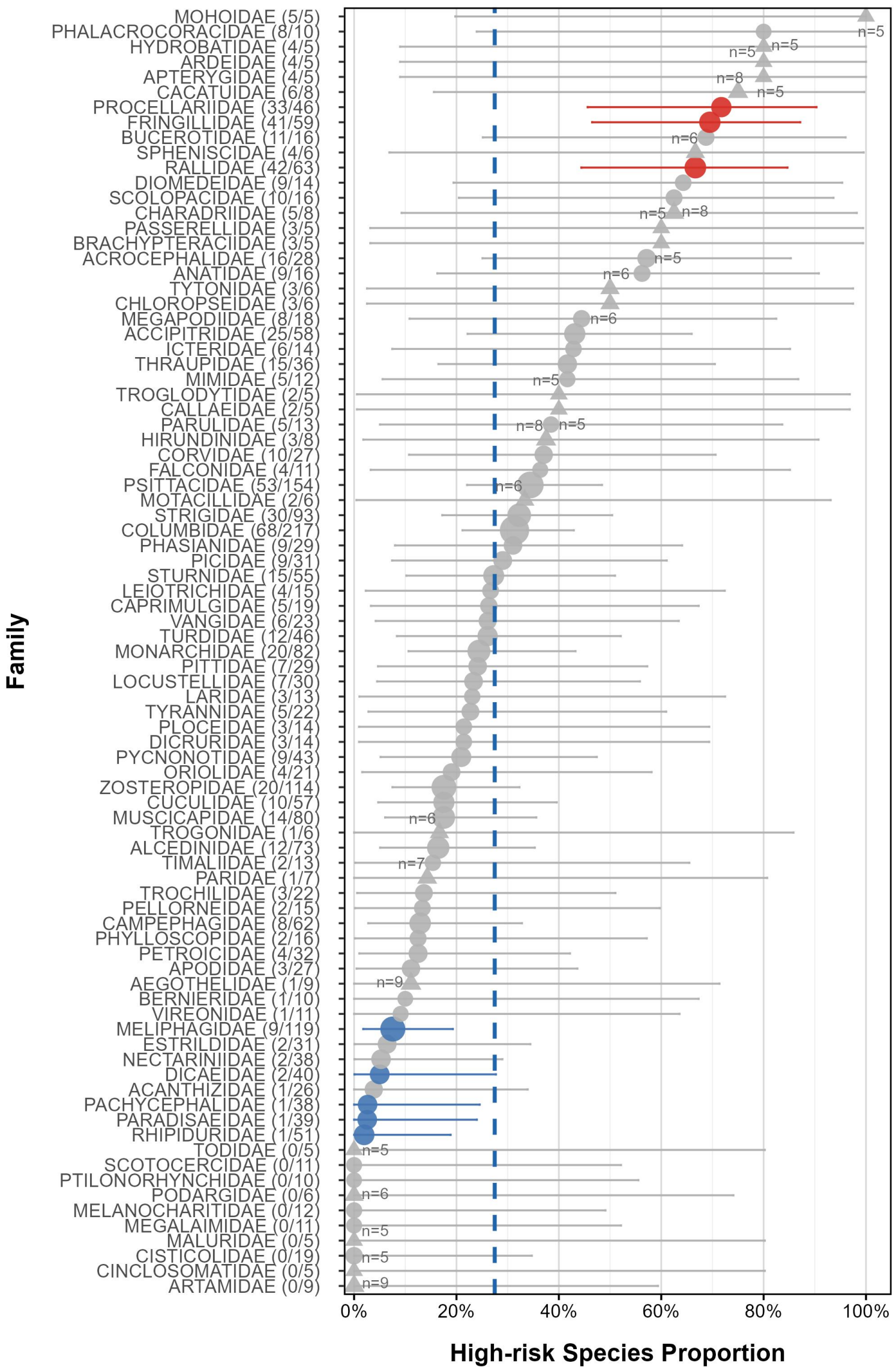

Fig. S2. Phylogenetic clustering of extinction risk across families of island endemic mammals (Mammalia)

The proportion of high-risk species across families of island endemic mammals. Only families with at least five island endemic species ($n \geq 5$) were statistically tested. Points indicate the observed proportion of high-risk species within each family, with horizontal error bars showing confidence intervals from exact binomial tests. The dashed line represents the class-level background extinction risk proportion (52.73%), calculated from all island endemic mammal species. Significance was evaluated using two-sided exact binomial tests with Dunn–Šidák correction applied within the class. Red points indicate families with significantly higher proportions than the background, blue points indicate significantly lower proportions, and grey points indicate non-significant results. Point size is proportional to the number of island endemic species in each family.Triangles represent families with 5–9 species, and circles represent families with 10 or more species.

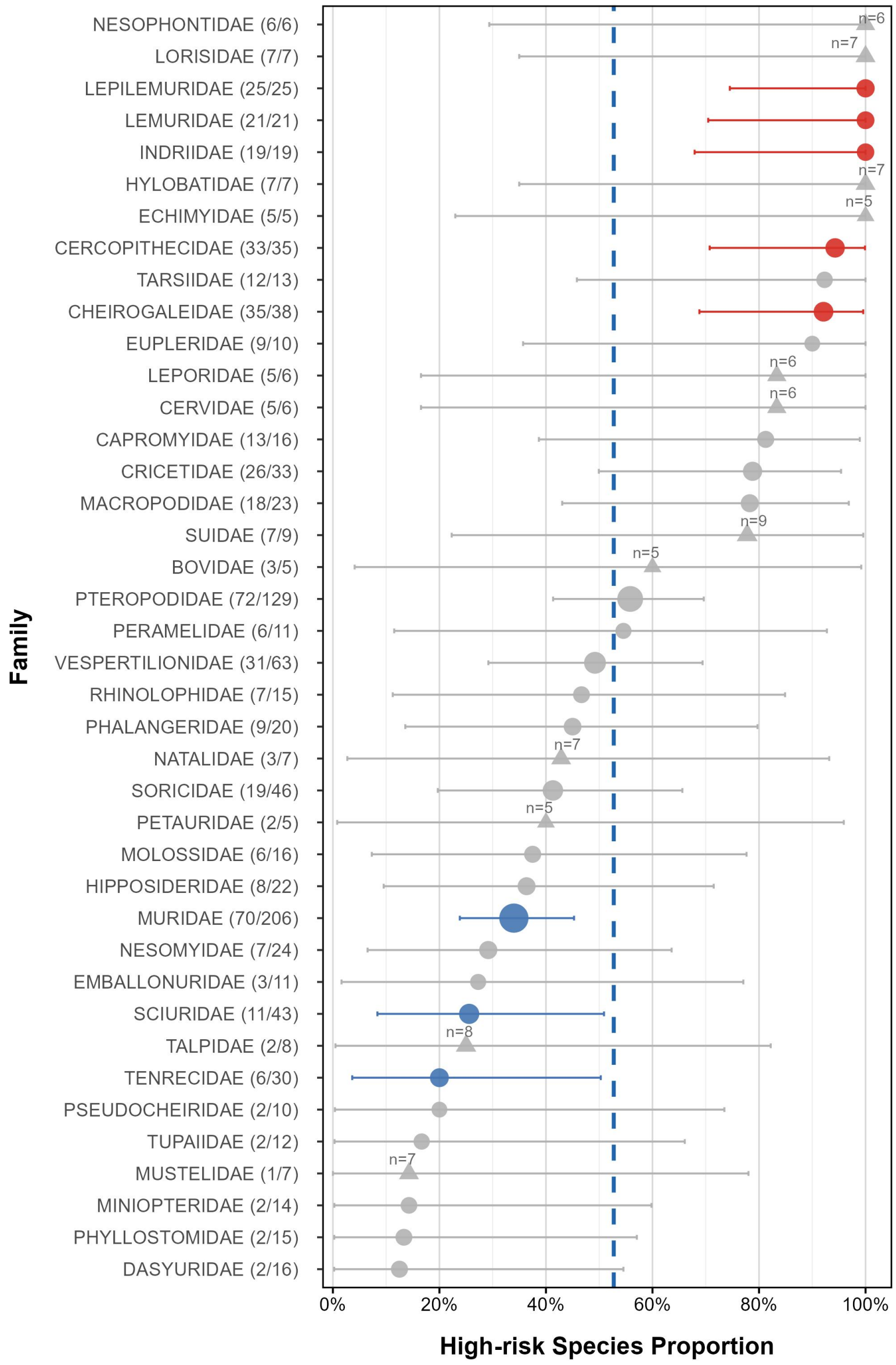

Fig. S3. Phylogenetic clustering of extinction risk across families of island endemic amphibians (Amphibia)

The proportion of high-risk species across families of island endemic amphibians. Only families with at least five island endemic species ($n \geq 5$) were statistically tested. Points represent the observed proportion of high-risk species within each family, with horizontal error bars showing confidence intervals from exact binomial tests. The dashed line indicates the class-level background extinction risk proportion (41.58%), calculated from all island endemic amphibian species. Significance was assessed using two-sided exact binomial tests with Dunn–Šidák correction applied within the class. Red points indicate families with significantly higher proportions than the background, blue points indicate significantly lower proportions, and grey points indicate non-significant results. Point size is proportional to the number of island endemic species in each family. Triangles represent families with 5–9 species, and circles represent families with 10 or more species.

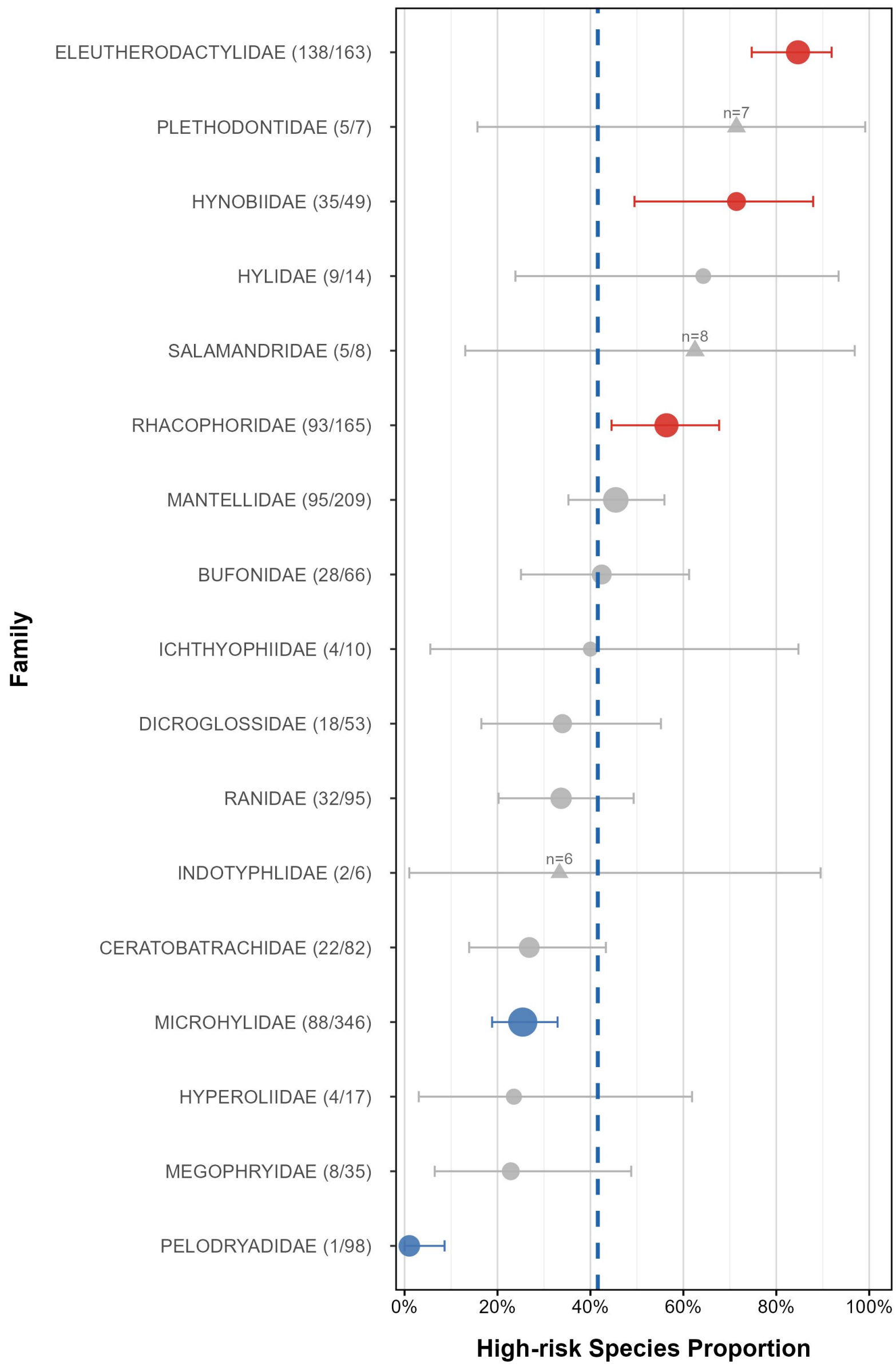

Fig. S4. Phylogenetic clustering of extinction risk across families of island endemic reptiles (Reptilia)

The proportion of high-risk species across families of island endemic reptiles. Only families with at least five island endemic species (n ≥ 5) were statistically tested. Points show the observed proportion of high-risk species within each family, with horizontal error bars representing confidence intervals from exact binomial tests. The dashed line denotes the class-level background extinction risk proportion (38.10%), calculated from all island endemic reptile species. Significance was evaluated using two-sided exact binomial tests with Dunn–Šidák correction applied within the class. Orange points indicate families with significantly higher proportions than the background, blue points indicate significantly lower proportions, and grey points indicate non-significant results. Point size is proportional to the number of island endemic species in each family. Triangles represent families with 5 – 9 species, and circles represent families with 10 or more species.

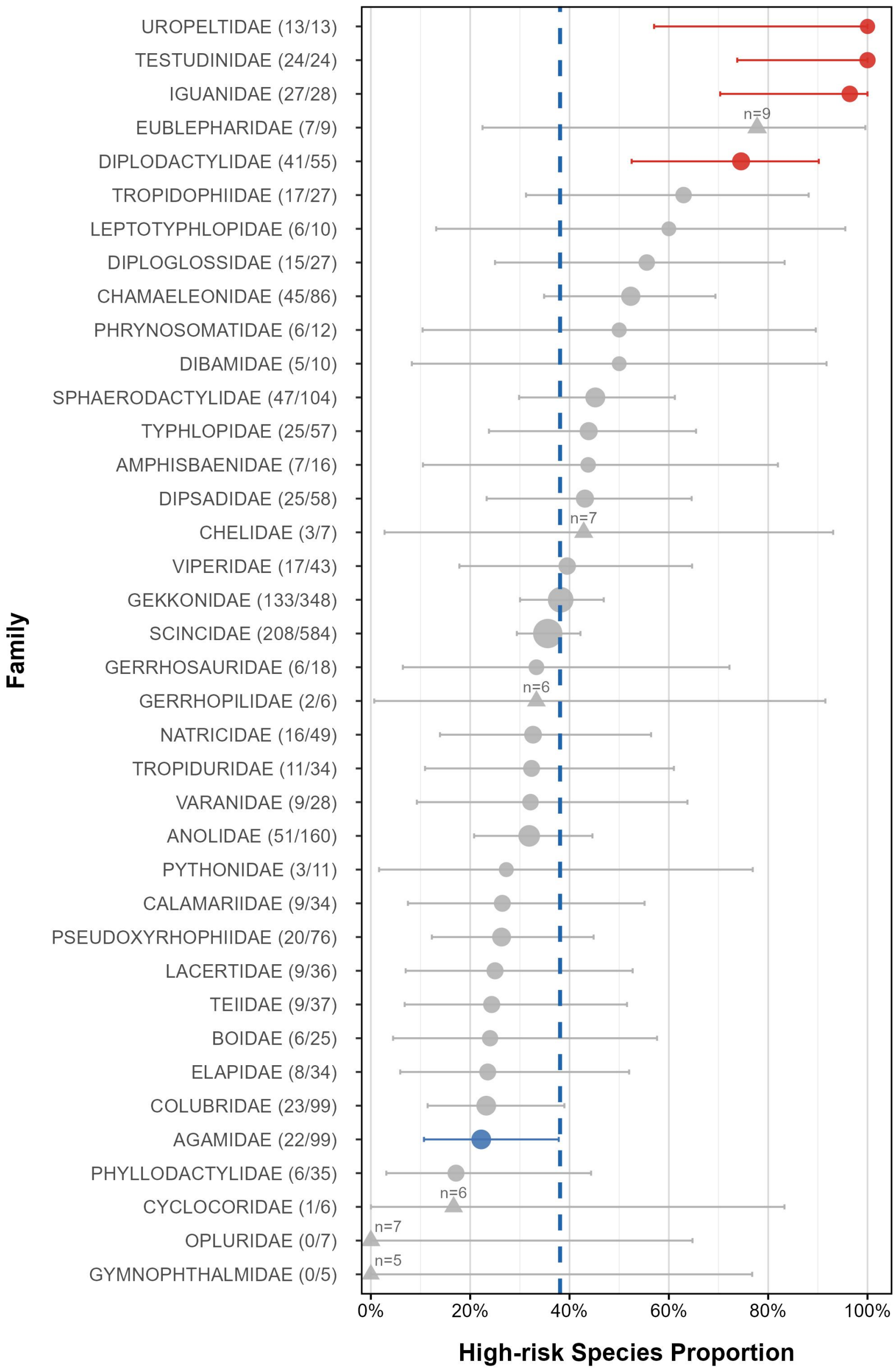

Fig. S5. Univariate phylogenetic regressions identify consistent correlates of extinction risk across terrestrial vertebrates

For ordinal extinction risk categories (0–5, from Least Concern to Extinct), univariate phylogenetic linear model-averaged estimates derived from 100 paired datasets are shown, combined using Rubin's rules to incorporate both within- and between-dataset variance. Panels a–d show results for (a) birds, (b) mammals, (c) amphibians, and (d) reptiles. Colours distinguish model types: red indicates models including all species, and blue indicates models restricted to species not listed as threatened under IUCN Criterion B or D2. Points represent model estimates, with horizontal bars indicating confidence intervals. Transparency denotes statistical significance, and asterisks indicate significance levels (*** $p < 0.001$, ** $p < 0.01$, * $p < 0.05$). Facets separate species by endemicity type: all species, island endemics, continent endemics, and dual-range species occurring in both regions.

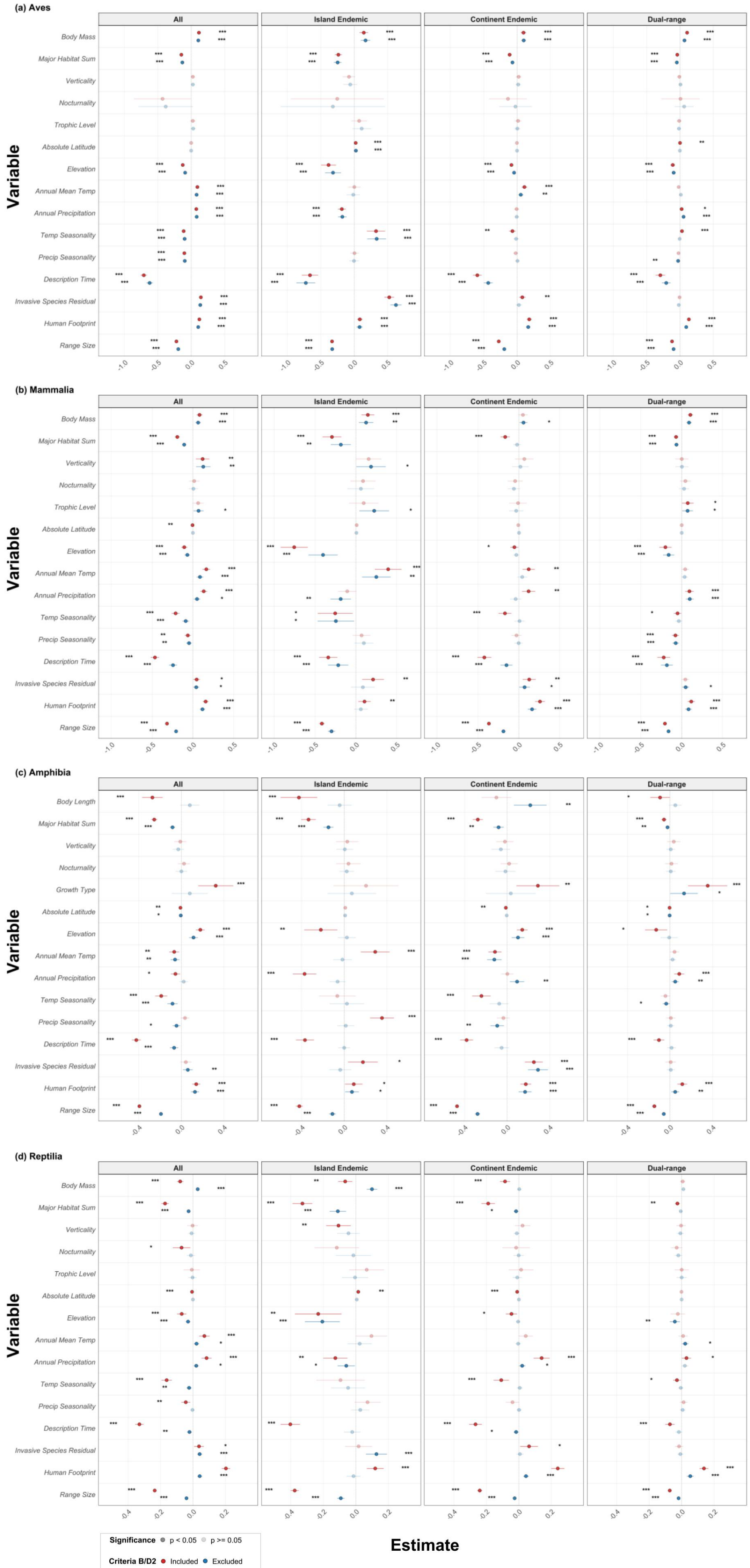
(a) Aves
All
Island Endemic
Continent Endemic
Dual-range
Body Mass
Major Habitat Sum
Verticality
Nocturnality
Trophic Level
Absolute Latitude
Elevation
Annual Mean Temp
Annual Precipitation
Temp Seasonality
Precip Seasonality
Description Time
Invasive Species Residual
Human Footprint
Range Size
Variable
(b) Mammalia
All
Island Endemic
Continent Endemic
Dual-range
Body Mass
Major Habitat Sum
Verticality
Nocturnality
Trophic Level
Absolute Latitude
Elevation
Annual Mean Temp
Annual Precipitation
Temp Seasonality
Precip Seasonality
Description Time
Invasive Species Residual
Human Footprint
Range Size
Variable
(c) Amphibia
All
Island Endemic
Continent Endemic
Dual-range
Body Length
Major Habitat Sum
Verticality
Nocturnality
Growth Type
Absolute Latitude
Elevation
Annual Mean Temp
Annual Precipitation
Temp Seasonality
Precip Seasonality
Description Time
Invasive Species Residual
Human Footprint
Range Size
Variable
(d) Reptilia
All
Island Endemic
Continent Endemic
Dual-range
Body Mass
Major Habitat Sum
Verticality
Nocturnality
Trophic Level
Absolute Latitude
Elevation
Annual Mean Temp
Annual Precipitation
Temp Seasonality
Precip Seasonality
Description Time
Invasive Species Residual
Human Footprint
Range Size
Variable
Significance ● p < 0.05 ● p >= 0.05
Criteria B/D2 ● Included ● Excluded
Estimate

REFERENCES

1. A. D. Barnosky, N. Matzke, S. Tomiya, G. O. U. Wogan, B. Swartz, T. B. Quental, C. Marshall, J. L. McGuire, E. L. Lindsey, K. C. Maguire, B. Mersey, E. A. Ferrer, Has the Earth's sixth mass extinction already arrived? *Nature* **471**, 51–57 (2011).

2. R. H. Cowie, P. Bouchet, B. Fontaine, The sixth mass extinction: Fact, fiction or speculation? *Biol. Rev.* **97**, 640–663 (2022).

3. J. E. M. Watson, N. Dudley, D. B. Segan, M. Hockings, The performance and potential of protected areas. *Nature* **515**, 67–73 (2014).

4. S. L. Pimm, C. N. Jenkins, R. Abell, T. M. Brooks, J. L. Gittleman, L. N. Joppa, P. H. Raven, C. M. Roberts, J. O. Sexton, The biodiversity of species and their rates of extinction, distribution, and protection. *Science* **344**, 1246752 (2014).

5. G. Ceballos, P. R. Ehrlich, A. D. Barnosky, A. Garcia, R. M. Pringle, T. M. Palmer, Accelerated modern human-induced species losses: Entering the sixth mass extinction. *Sci. Adv.* **1**, e1400253 (2015).

6. J. C. Garcia-R, M. Cimatti, M. Di Marco, Past and recent drivers of extinction risk in endemic New Zealand birds. *Anim. Conserv.* **28**, 436–444 (2025).

7. M. C. Urban, Climate change extinctions. *Science* **386**, 1123–1128 (2024).

8. N. Myers, R. A. Mittermeier, C. G. Mittermeier, G. A. B. Da Fonseca, J. Kent, Biodiversity hotspots for conservation priorities. *Nature* **403**, 853–858 (2000).

9. A. Auber, C. Waldock, A. Maire, E. Goberville, C. Albouy, A. C. Algar, M. McLean, A. Brind'Amour, A. L. Green, M. Tupper, A functional vulnerability framework for biodiversity conservation. *Nat. Commun.* **13**, 4774 (2022).

10. J. M. Fernández-Palacios, H. Kreft, S. D. H. Irl, S. Norder, C. Ah-Peng, P. A. V. Borges, K. C. Burns, L. De Nascimento, J.-Y. Meyer, E. Montes, D. R. Drake, Scientists' warning–The outstanding biodiversity of islands is in peril. *Glob. Ecol. Conserv.* **31**, e01847 (2021).

11. S. Llorente-Culebras, C. P. Carmona, W. D. Carvalho, A. Menegotto, R. Molina-Venegas, R. J. Ladle, A. M. C. Santos, Island biodiversity in peril: Anticipating a loss of mammals' functional diversity with future species extinctions. *Glob. Chang. Biol.* **30**, e17375 (2024).

12. R. Sayre, S. Noble, S. Hamann, R. Smith, D. Wright, S. Breyer, K. Butler, K. Van Graafeiland, C. Frye, D. Karagulle, D. Hopkins, D. Stephens, K. Kelly, Z. Basher, D. Burton, J. Cress, K. Atkins, D. P. Van Sistine, B. Friesen, R. Allee, T. Allen, P. Aniello, I. Asaad, M. J. Costello, K. Goodin, P. Harris, M. Kavanaugh, H. Lillis, E. Manca, F. Muller-Karger, B. Nyberg, R. Parsons, J. Saarinen, J. Steiner, A. Reed, A new 30 meter resolution global shoreline vector and associated global islands database for the development of standardized ecological coastal units. *J. Oper. Oceanogr.* **12**, S47–S56 (2019).

13. G. Kier, H. Kreft, T. M. Lee, W. Jetz, P. L. Ibisch, C. Nowicki, J. Mutke, W. Barthlott, A global assessment of endemism and species richness across island and mainland regions. *Proc. Natl. Acad. Sci. U.S.A.* **106**, 9322–9327 (2009).

14. B. R. Tershy, K.-W. Shen, K. M. Newton, N. D. Holmes, D. A. Croll, The importance of islands for the protection of biological and linguistic diversity. *BioScience* **65**, 592–597 (2015).

15. K. E. Saban, J. J. Wiens, Unpacking the extinction crisis: Rates, patterns and causes of recent extinctions in plants and animals. *Proc. R. Soc. B. Biol. Sci.* **292**, 20251717 (2025).

16. T. Roedl, S. Berger, L. M. Romero, M. Wikelski, Tameness and stress physiology in a predator-naive island species confronted with novel predation threat. *Proc. R. Soc. B. Biol. Sci.* **274**, 577–582 (2007).

17. K. C. Rosenblad, D. L. Perret, D. F. Sax, Niche syndromes reveal climate-driven extinction threat to island endemic conifers. *Nat. Clim. Chang.* **9**, 627–631 (2019).

18. S. Baeckens, R. Van Damme, The island syndrome. *Curr. Biol.* **30**, R338–R339 (2020).

19. R. J. Whittaker, J. M. Fernández-Palacios, T. J. Matthews, M. K. Borregaard, K. A. Triantis, Island biogeography: Taking the long view of nature's laboratories. *Science* **357**, eaam8326 (2017).

20. J. R. Wood, J. A. Alcover, T. M. Blackburn, P. Bover, R. P. Duncan, J. P. Hume, J. Louys, H. J. M. Meijer, J. C. Rando, J. M. Wilmshurst, Island extinctions: Processes, patterns, and potential for ecosystem restoration. *Environ. Conserv.* **44**, 348–358 (2017).

21. The IUCN Red List of Threatened Species, www.iucnredlist.org.

22. T. J. Matthews, J. P. Wayman, P. Cardoso, F. Sayol, J. P. Hume, W. Ulrich, J. A. Tobias, F. C. Soares, C. Thébaud, T. E. Martin, K. A. Triantis, Threatened and extinct island endemic birds of the world: Distribution, threats and functional diversity. *J. Biogeogr.* **49**, 1920–1940 (2022).

23. P. Pysek, P. E. Hulme, D. Simberloff, S. Bacher, T. M. Blackburn, J. T. Carlton, W. Dawson, F. Essl, L. C. Foxcroft, P. Genovesi, J. M. Jeschke, I. Kuehn, A. M. Liebhold, N. E. Mandrak, L. A. Meyerson, A. Pauchard, J. Pergl, H. E. Roy, H. Seebens, M. van Kleunen, M. Vila, M. J. Wingfield, D. M. Richardson, Scientists' warning on invasive alien species. *Biol. Rev.* **95**, 1511–1534 (2020).

24. C. J. Brandon, W. D. Pearse, J. P. Herrera, The intrinsic and extrinsic drivers of extinction risk in lemurs (Lemuroidea). *Biol. Conserv.* **290**, 110408 (2024).

25. M. Cardillo, G. M. Mace, K. E. Jones, J. Bielby, O. R. P. Bininda-Emonds, W. Sechrest, C. D. L. Orme, A. Purvis, Multiple causes of high extinction risk in large mammal species. *Science* **309**, 1239–1241 (2005).

26. S. Ducatez, R. Shine, Drivers of extinction risk in terrestrial vertebrates. *Conserv. Lett.* **10**, 186–194 (2017).

27. W. J. Ripple, C. Wolf, T. M. Newsome, M. Hoffmann, A. J. Wirsing, D. J. McCauley, Extinction risk is most acute for the world's largest and smallest vertebrates. *Proc. Natl. Acad. Sci. U.S.A.* **114**, 10678–10683 (2017).

28. A. G. Boyer, Consistent ecological selectivity through time in pacific island avian extinctions. *Conserv. Biol.* **24**, 511–519 (2010).

29. R. Rozzi, M. V. Lomolino, A. A. E. Van Der Geer, D. Silvestro, S. K. Lyons, P. Bover, J. A. Alcover, A. Benítez-López, C.-H. Tsai, M. Fujita, M. O. Kubo, J. Ochoa, M. E. Scarborough, S. T. Turvey, A. Zizka, J. M. Chase, Dwarfism and gigantism drive human-mediated extinctions on islands. *Science* **379**, 1054–1059 (2023).

30. Y. Xiong, R. Rozzi, Y. Zhang, L. Fan, J. Zhao, D. Li, Y. Yao, H. Xiao, J. Liu, X. Zeng, H. Xu, Y. Jiang, F. Lei, Convergent evolution toward a slow pace of life predisposes insular endotherms to anthropogenic extinctions. *Sci. Adv.* **10**, eadm8240 (2024).

31. R. Cooke, F. Sayol, T. Andermann, T. M. Blackburn, M. J. Steinbauer, A. Antonelli, S. Faurby, Undiscovered bird extinctions obscure the true magnitude of human-driven extinction waves. *Nat. Commun.* **14**, 8116 (2023).

32. E. Barreto, T. F. Rangel, L. Pellissier, C. H. Graham, Area, isolation and climate explain the diversity of mammals on islands worldwide. *Proc. R. Soc. B. Biol. Sci.* **288**, 20211879 (2021).

33. J. Foufopoulos, A. M. Kilpatrick, A. R. Ives, Climate change and elevated extinction rates of reptiles from mediterranean islands. *Am. Nat.* **177**, 119–129 (2011).

34. F. Orgeret, A. Thiebault, K. M. Kovacs, C. Lydersen, M. A. Hindell, S. A. Thompson, W. J. Sydeman, P. A. Pistorius, Climate change impacts on seabirds and marine mammals: The importance of study duration, thermal tolerance and generation time. *Ecol. Lett.* **25**, 218–239 (2022).

35. R. L. White, P. M. Bennett, Elevational distribution and extinction risk in birds. *PLOS ONE* **10**, e0121849 (2015).

36. M. Böhm, R. Williams, H. R. Bramhall, K. M. McMillan, A. D. Davidson, A. Garcia, L. M. Bland, J. Bielby, B. Collen, Correlates of extinction risk in squamate reptiles: The relative

importance of biology, geography, threat and range size. *Glob. Ecol. Biogeogr.* **25**, 391–405 (2016).

37. J. Guirguis, L. E. B. Goodyear, C. Finn, J. V. Johnson, D. Pincheira-Donoso, Risk of extinction increases towards higher elevations across the world's amphibians. *Glob. Ecol. Biogeogr.* **32**, 1952–1963 (2023).

38. A. A. Hoffmann, C. M. Sgrò, Climate change and evolutionary adaptation. *Nature* **470**, 479–485 (2011).

39. C. Chen, J. Wang, M. Holyoak, L. Lin, Y. Wang, Global assessment of current extinction risks and future challenges for turtles and tortoises. *Nat. Commun.* **16**, 7114 (2025).

40. P. H. Verburg, K. Neumann, L. Nol, Challenges in using land use and land cover data for global change studies. *Glob. Chang. Biol.* **17**, 974–989 (2011).

41. R. Tingley, R. A. Hitchmough, D. G. Chapple, Life-history traits and extrinsic threats determine extinction risk in New Zealand lizards. *Biol. Conserv.* **165**, 62–68 (2013).

42. S. Nogué, A. M. C. Santos, H. J. B. Birks, S. Björck, A. Castilla-Beltrán, S. Connor, E. J. de Boer, L. de Nascimento, V. A. Felde, J. M. Fernández-Palacios, C. A. Froyd, S. G. Haberle, H. Hooghiemstra, K. Ljung, S. J. Norder, J. Peñuelas, M. Prebble, J. Stevenson, R. J. Whittaker, K. J. Willis, J. M. Wilmshurst, M. J. Steinbauer, The human dimension of biodiversity changes on islands. *Science* **372**, 488–491 (2021).

43. T. M. Blackburn, P. Cassey, R. P. Duncan, K. L. Evans, K. J. Gaston, Avian extinction and mammalian introductions on oceanic islands. *Science* **305**, 1955–1958 (2004).

44. D. R. Spatz, K. M. Zilliacus, N. D. Holmes, S. H. M. Butchart, P. Genovesi, G. Ceballos, B. R. Tershy, D. A. Croll, Globally threatened vertebrates on islands with invasive species. *Sci. Adv.* **3**, e1603080 (2017).

45. K. Liversage, J. Kotta, I. Kuprijanov, M. Rätsep, K. Nõomaa, A trophic cascade facilitates native habitat providers within assemblages of multiple invasive marine species. *Ecosphere* **12**, e03621 (2021).

46. D. A. Croll, J. L. Maron, J. A. Estes, E. M. Danner, G. V. Byrd, Introduced predators transform subarctic islands from grassland to tundra. *Science* **307**, 1959–1961 (2005).

47. J. H. Heinen, E. E. Van Loon, D. M. Hansen, W. D. Kissling, Extinction-driven changes in frugivore communities on oceanic islands. *Ecography* **41**, 1245–1255 (2018).

48. J. H. Heinen, F. B. V. Florens, C. Baider, J. P. Hume, W. D. Kissling, R. J. Whittaker, C. Rahbek, M. K. Borregaard, Novel plant–frugivore network on Mauritius is unlikely to compensate for the extinction of seed dispersers. *Nat. Commun.* **14**, 1019 (2023).

49. B. C. Scheele, F. Pasmans, L. F. Skerratt, L. Berger, A. Martel, W. Beukema, A. A. Acevedo, P. A. Burrowes, T. Carvalho, A. Catenazzi, I. De la Riva, M. C. Fisher, S. V. Flechas, C. N. Foster, P. Frías-Álvarez, T. W. J. Garner, B. Gratwicke, J. M. Guayasamin, M. Hirschfeld, J. E. Kolby, T. A. Kosch, E. La Marca, D. B. Lindenmayer, K. R. Lips, A. V. Longo, R. Maneyro, C. A. McDonald, J. Mendelson, P. Palacios-Rodriguez, G. Parra-Olea, C. L. Richards-Zawacki, M.-O. Rödel, S. M. Rovito, C. Soto-Azat, L. F. Toledo, J. Voyles, C. Weldon, S. M. Whitfield, M. Wilkinson, K. R. Zamudio, S. Canessa, Amphibian fungal panzootic causes catastrophic and ongoing loss of biodiversity. *Science* **363**, 1459–1463 (2019).

50. J. A. Luedtke, J. Chanson, K. Neam, L. Hobin, A. O. Maciel, A. Catenazzi, A. Borzée, A. Hamidy, A. Aowphol, A. Jean, Á. Sosa-Bartuano, A. Fong G, A. de Silva, A. Fouquet, A. Angulo, A. A. Kidov, A. M. Saravia, A. C. Diesmos, A. Tominaga, B. Shrestha, B. Gratwicke, B. Tjaturadi, C. C. M. Rivera, C. R. V. Almazán, C. Señaris, S. R. Chandramouli, C. Strüssmann, C. F. C. Fernández, C. Azat, C. J. Hoskin, C. Hilton-Taylor, D. L. Whyte, D. J. Gower, D. H. Olson, D. F. Cisneros-Heredia, D. J. Santana, E. Nagombi, E. Najafi-Majd, E. S. H. Quah, F. Bolaños, F. Xie, F. Brusquetti, F. S. Álvarez, F. Andreone, F. Glaw, F. E. Castañeda, F. Kraus, G. Parra-Olea, G. Chaves, G. F. Medina-Rangel, G. González-Durán, H. M. Ortega-Andrade, I. F. Machado, I. Das, I. R. Dias, J. N. Urbina-Cardona, J. Crnobrnja-Isailović, J.-H. Yang, J. Jianping, J. T. Wangyal, J. J. L. Rowley, J. Measey, K. Vasudevan, K. O. Chan, K. V. Gururaja, K. Ovaska, L. C. Warr, L. Canseco-Márquez, L. F. Toledo, L. M. Díaz, M. M. H. Khan, M. Meegaskumbura, M. E. Acevedo, M. F. Napoli, M. A. Ponce, M. Vaira, M. Lampo, M. H. Yánez-Muñoz, M. D. Scherz, M.-O. Rödel, M. Matsui, M. Fildor,

M. D. Kusrini, M. F. Ahmed, M. Rais, N. G. Kouamé, N. García, N. L. Gonwouo, P. A. Burrowes, P. Y. Imbun, P. Wagner, P. J. R. Kok, R. L. Joglar, R. J. Auguste, R. A. Brandão, R. Ibáñez, R. von May, S. B. Hedges, S. D. Biju, S. R. Ganesh, S. Wren, S. Das, S. V. Flechas, S. L. Ashpole, S. J. Robleto-Hernández, S. P. Loader, S. J. Incháustegui, S. Garg, S. Phimmachak, S. J. Richards, T. Slimani, T. Osborne-Naikatini, T. P. F. Abreu-Jardim, T. H. Condez, T. R. De Carvalho, T. P. Cutajar, T. W. Pierson, T. Q. Nguyen, U. Kaya, Z. Yuan, B. Long, P. Langhammer, S. N. Stuart, Ongoing declines for the world's amphibians in the face of emerging threats. *Nature* **622**, 308–314 (2023).

51. F. Gonçalves, H. Farooq, M. Harfoot, M. M. Pires, N. Villar, L. Sales, C. Carvalho, C. Bello, C. Emer, R. S. Bovendorp, C. Mendes, G. Beca, L. Lautenschlager, Y. Souza, F. Pedrosa, C. Paz, V. B. Zipparro, P. Akkawi, W. Bercê, F. Farah, A. V. L. Freitas, L. F. Silveira, F. Olmos, J. Geldmann, B. Dalsgaard, M. Galetti, A global map of species at risk of extinction due to natural hazards. *Proc. Natl. Acad. Sci. U.S.A.* **121**, e2321068121 (2024).

52. G. F. Ficetola, Habitat conservation research for amphibians: Methodological improvements and thematic shifts. *Biodivers. Conserv.* **24**, 1293–1310 (2015).

53. J. R. Wood, J. M. Wilmshurst, T. H. Worthy, A. S. Holzapfel, A. Cooper, A lost link between a flightless parrot and a parasitic plant and the potential role of coprolites in conservation paleobiology. *Conserv. Biol.* **26**, 1091–1099 (2012).

54. T. J. Matthews, K. A. Triantis, J. P. Wayman, T. E. Martin, J. P. Hume, P. Cardoso, S. Faurby, C. D. Mendenhall, P. Dufour, F. Rigal, R. Cooke, R. J. Whittaker, A. L. Pigot, C. Thébaud, M. W. Jørgensen, E. Benavides, F. C. Soares, W. Ulrich, Y. Kubota, J. P. Sadler, J. A. Tobias, F. Sayol, The global loss of avian functional and phylogenetic diversity from anthropogenic extinctions. *Science* **386**, 55–60 (2024).

55. M. Galetti, R. Guevara, M. C. Côrtes, R. Fadini, S. Von Matter, A. B. Leite, F. Labecca, T. Ribeiro, C. S. Carvalho, R. G. Collevatti, M. M. Pires, P. R. Guimarães, P. H. Brancalion, M. C. Ribeiro, P. Jordano, Functional extinction of birds drives rapid evolutionary changes in seed size. *Science* **340**, 1086–1090 (2013).

56. R. M. Pringle, J. O. Abraham, T. M. Anderson, T. C. Coverdale, A. B. Davies, C. L. Dutton, A. Gaylard, J. R. Goheen, R. M. Holdo, M. C. Hutchinson, D. M. Kimuyu, R. A. Long, A. L. Subalusky, M. P. Veldhuis, Impacts of large herbivores on terrestrial ecosystems. *Curr. Biol.* **33**, R584–R610 (2023).

57. R. Dirzo, H. S. Young, M. Galetti, G. Ceballos, N. J. B. Isaac, B. Collen, Defaunation in the anthropocene. *Science* **345**, 401–406 (2014).

58. H. S. Young, D. J. McCauley, M. Galetti, R. Dirzo, Patterns, causes, and consequences of anthropocene defaunation. *Annu. Rev. Ecol. Evol. Syst.* **47**, 333–358 (2016).

59. K. R. McConkey, D. R. Drake, Low redundancy in seed dispersal within an island frugivore community. *AoB Plants* **7**, plv088 (2015).

60. K. A. Wilson, M. F. McBride, M. Bode, H. P. Possingham, Prioritizing global conservation efforts. *Nature* **440**, 337–340 (2006).

61. D. Simberloff, L. G. Abele, Refuge design and island biogeographic theory: Effects of fragmentation. *Am. Nat.* **120**, 41–50 (1982).

62. L. Fahrig, J. I. Watling, C. A. Arnillas, V. Arroyo-Rodríguez, T. Jörger-Hickfang, J. Müller, H. M. Pereira, F. Riva, V. Rösch, S. Seibold, T. Tscharntke, F. May, Resolving the SLOSS dilemma for biodiversity conservation: A research agenda. *Biol. Rev.* **97**, 99–114 (2022).

63. R. J. Whittaker, J. M. Fernández-Palacios, T. J. Matthews, "The biogeography of island life: Biodiversity hotspots in context," in *Island Biogeography: Geo-Environmental Dynamics, Ecology, Evolution, Human Impact, and Conservation*, R. J. Whittaker, J. M. Fernández-Palacios, T. J. Matthews, Eds. (Oxford Univ. Press, 2023), 57–90.

64. Y. Li, Y. Wang, X. Liu, Half of global islands have reached critical area thresholds for undergoing rapid increases in biological invasions. *Proc. R. Soc. B. Biol. Sci.* **291**, rspb.2024.0844 (2024).

65. G. Li, C. Fang, J. E. M. Watson, S. Sun, W. Qi, Z. Wang, J. Liu, Mixed effectiveness of global protected areas in resisting habitat loss. *Nat. Commun.* **15**, 8389 (2024).

66. M. T. Jezierski, W. J. Smith, S. M. Clegg, The island syndrome in birds. *J. Biogeogr.* **51**, 1607–1622 (2024).

67. M. J. A. Creighton, C. L. Nunn, Explaining the primate extinction crisis: Predictors of extinction risk and active threats. *Proc. R. Soc. B. Biol. Sci.* **290**, 20231441 (2023).

68. D. M. Hansen, C. J. Donlan, C. J. Griffiths, K. J. Campbell, Ecological history and latent conservation potential: Large and giant tortoises as a model for taxon substitutions. *Ecography* **33**, 272–284 (2010).

69. G. Olah, S. H. M. Butchart, A. Symes, I. M. Guzmán, R. Cunningham, D. J. Brightsmith, R. Heinsohn, Ecological and socio-economic factors affecting extinction risk in parrots. *Biodivers. Conserv.* **25**, 205–223 (2016).

70. J.-M. Roberge, P. Angelstam, Usefulness of the umbrella species concept as a conservation tool. *Conserv. Biol.* **18**, 76–85 (2004).

71. M. Branton, J. S. Richardson, Assessing the value of the umbrella-species concept for conservation planning with meta-analysis. *Conserv. Biol.* **25**, 9–20 (2011).

72. I. Breckheimer, N. M. Haddad, W. F. Morris, A. M. Trainor, W. R. Fields, R. T. Jobe, B. R. Hudgens, A. Moody, J. R. Walters, Defining and evaluating the umbrella species concept for conserving and restoring landscape connectivity. *Conserv. Biol.* **28**, 1584–1593 (2014).

73. N. S. Upham, Past and present of insular Caribbean mammals: Understanding Holocene extinctions to inform modern biodiversity conservation. *J. Mammal.* **98**, 913–917 (2017).

74. P. Pottier, M. R. Kearney, N. C. Wu, A. R. Gunderson, J. E. Rej, A. N. Rivera-Villanueva, P. Pollo, S. Burke, S. M. Drobniak, S. Nakagawa, Vulnerability of amphibians to global warming. *Nature* **639**, 954–961 (2025).

75. C. Leclerc, F. Courchamp, C. Bellard, Insular threat associations within taxa worldwide. *Sci. Rep.* **8**, 6393 (2018).

76. M. J. Munstermann, N. A. Heim, D. J. McCauley, J. L. Payne, N. S. Upham, S. C. Wang, M. L. Knope, A global ecological signal of extinction risk in terrestrial vertebrates. *Conserv. Biol.* **36**, e13852 (2022).

77. G. M. Mace, N. J. Collar, K. J. Gaston, C. Hilton-Taylor, H. R. Akçakaya, N. Leader-Williams, E. J. Milner-Gulland, S. N. Stuart, Quantification of extinction risk: IUCN's system for classifying threatened species. *Conserv. Biol.* **22**, 1424–1442 (2008).

78. J. Borgelt, M. Dorber, M. A. Høiberg, F. Verones, More than half of data deficient species predicted to be threatened by extinction. *Commun. Biol.* **5**, 679 (2022).

79. I. R. Geijzendorffer, E. C. Regan, H. M. Pereira, L. Brotons, N. Brummitt, Y. Gavish, P. Haase, C. S. Martin, J.-B. Mihoub, C. Secades, D. S. Schmeller, S. Stoll, F. T. Wetzel, M. Walters, Bridging the gap between biodiversity data and policy reporting needs: An essential biodiversity variables perspective. *J. Appl. Ecol.* **53**, 1341–1350 (2016).

80. R. H. MacArthur, E. O. Wilson, *The Theory of Island Biogeography* (Princeton Univ. Press, 1967).

81. R. Frankham, D. A. Briscoe, J. D. Ballou, *Introduction to Conservation Genetics* (Cambridge Univ. Press, 2012).

82. Y. Wang, X. Si, P. M. Bennett, C. Chen, D. Zeng, Y. Zhao, Y. Wu, P. Ding, Ecological correlates of extinction risk in Chinese birds. *Ecography* **41**, 782–794 (2018).

83. S. Zhang, S. Meiri, M. Holyoak, J. Wang, Y. Wang, C. Chen, Demand for small- and large-ranged reptiles in worldwide wildlife trade. *Conserv. Biol.* **39**, e70095 (2025).

84. R. K. Kopf, S. Banks, L. J. N. Brent, P. Humphries, C. J. Jolly, P. C. Lee, O. J. Luiz, D. Nimmo, K. O. Winemiller, Loss of Earth's old, wise, and large animals. *Science* **387**, eado2705 (2025).

85. M. Cardillo, Clarifying the relationship between body size and extinction risk in amphibians by complete mapping of model space. *Proc. R. Soc. B. Biol. Sci.* **288**, 20203011 (2021).

86. S. Matsui, M. Hisaka, M. Takagi, Arboreal nesting and utilization of open-cup bird nests by introduced Ship Rats *Rattus rattus* on an oceanic island. *Bird Conserv. Int.* **20**, 34–42 (2010).

87. A. H. Nance, W. F. Mitchell, F. Dawlings, C. N. Cook, R. H. Clarke, Rodent predation and specialised avian habitat requirements drive extinction risk for endemic island songbirds in the south-west Pacific. *Emu* **123**, 217–231 (2023).

88. J. M. Hoare, S. Pledger, N. J. Nelson, C. H. Daugherty, Avoiding aliens: Behavioural plasticity in habitat use enables large, nocturnal geckos to survive Pacific rat invasions. *Biol. Conserv.* **136**, 510–519 (2007).

89. N. P. Mohanty, S. Harikrishnan, K. Vasudevan, Watch out where you sleep: Nocturnal sleeping behaviour of Bay Island lizards. *PeerJ* **4**, e1856 (2016).

90. S. Baeckens, S. Meiri, R. Shine, Foraging mode affects extinction risk of snakes and lizards, but in different ways. *Conserv. Lett.* **16**, e12977 (2023).

91. F. Chichorro, F. Urbano, D. Teixeira, H. Väre, T. Pinto, N. Brummitt, X. He, A. Hochkirch, J. Hyvönen, L. Kaila, A. Juslén, P. Cardoso, Trait-based prediction of extinction risk across terrestrial taxa. *Biol. Conserv.* **274**, 109738 (2022).

92. F. Sayol, M. J. Steinbauer, T. M. Blackburn, A. Antonelli, S. Faurby, Anthropogenic extinctions conceal widespread evolution of flightlessness in birds. *Sci. Adv.* **6**, eabb6095 (2020).

93. T. P. Leppard, E. E. Cochrane, D. Gaffney, C. L. Hofman, J. E. Laffoon, M. M. E. Bunbury, C. Broodbank, Global patterns in island colonization during the holocene. *J. World Prehist.* **35**, 163–232 (2022).

94. W. Beukema, F. Pasmans, S. Van Praet, F. Ferri-Yáñez, M. Kelly, A. E. Laking, J. Erens, J. Speybroeck, K. Verheyen, L. Lens, A. Martel, Microclimate limits thermal behaviour favourable to disease control in a nocturnal amphibian. *Ecol. Lett.* **24**, 27–37 (2021).

95. S. F. Nunes, K. Powell, P. Griffith, M. A. Carretero, R. Rebelo, M. Cabeza, R. Rocha, Island-restricted reptiles are more threatened but less studied than their mainland counterparts. *Conservat. Sci. and Prac.* **8**, e70184 (2025).

96. W. E. Cooper, R. A. Pyron, T. Garland, Island tameness: Living on islands reduces flight initiation distance. *Proc. R. Soc. B. Biol. Sci.* **281**, 20133019 (2014).

97. G. C. Cummins, T. C. Theimer, E. H. Paxton, Responses to terrestrial nest predators by endemic and introduced Hawaiian birds. *Ecol. Evol.* **10**, 1949–1958 (2020).

98. E. Montes, F. Kraus, B. Chergui, J. M. Pleguezuelos, Collapse of the endemic lizard *Podarcis pityusensis* on the island of Ibiza mediated by an invasive snake. *Curr. Zool.* **68**, 295–303 (2022).

99. H. P. Jones, N. D. Holmes, S. H. M. Butchart, B. R. Tershy, P. J. Kappes, I. Corkery, A. Aguirre-Muñoz, D. P. Armstrong, E. Bonnaud, A. A. Burbidge, K. Campbell, F. Courchamp, P. E. Cowan, R. J. Cuthbert, S. Ebbert, P. Genovesi, G. R. Howald, B. S. Keitt, S. W. Kress, C. M. Miskelly, S. Oppel, S. Poncet, M. J. Rauzon, G. Rocamora, J. C. Russell, A. Samaniego-Herrera, P. J. Seddon, D. R. Spatz, D. R. Towns, D. A. Croll, Invasive mammal eradication on islands results in substantial conservation gains. *Proc. Natl. Acad. Sci. U.S.A.* **113**, 4033–4038 (2016).

100. M. M. A. Boehm, Q. C. B. Cronk, Dark extinction: The problem of unknown historical extinctions. *Biol. Lett.* **17**, 20210007 (2021).

101. B. Leung, A. Gonzalez, Global monitoring for biodiversity: Uncertainty, risk, and power analyses to support trend change detection. *Sci. Adv.* **10**, eadj1448 (2024).

102. K. A. Triantis, P. A. V. Borges, R. J. Ladle, J. Hortal, P. Cardoso, C. Gaspar, F. Dinis, E. Mendonça, L. M. A. Silveira, R. Gabriel, C. Melo, A. M. C. Santos, I. R. Amorim, S. P. Ribeiro, A. R. M. Serrano, J. A. Quartau, R. J. Whittaker, Extinction debt on oceanic islands. *Ecography* **33**, 285–294 (2010).

103. J. Troudet, P. Grandcolas, A. Blin, R. Vignes-Lebbe, F. Legendre, Taxonomic bias in biodiversity data and societal preferences. *Sci. Rep.* **7**, 9132 (2017).

104. Y. Fourcade, Comparing species distributions modelled from occurrence data and from expert-based range maps. Implication for predicting range shifts with climate change. *Ecol. Inform.* **36**, 8–14 (2016).

105. V. Cazalis, L. Santini, P. M. Lucas, M. González-Suárez, M. Hoffmann, A. Benítez-López, M. Pacifici, A. M. Schipper, M. Böhm, A. Zizka, V. Clausnitzer, C. Meyer, M. Jung, S. H. M. Butchart, P. Cardoso, G. Mancini, H. R. Akçakaya, B. E. Young, G. Patoine, M. Di Marco, Prioritizing the reassessment of data-deficient species on the IUCN Red List. *Conserv. Biol.* **37**, e14139 (2023).

106. S. Chamberlain, rredlist: "IUCN" Red List Client, version 0.7.1 (2022); https://cran.r-project.org/web/packages/rredlist/index.html.

107. Natural Earth, Free vector and raster map data at 1:10m, 1:50m, and 1:110m scales; www.naturalearthdata.com/.

108. P. Weigelt, W. Jetz, H. Kreft, Bioclimatic and physical characterization of the world's islands. *Proc. Natl. Acad. Sci. U.S.A.* **110**, 15307–15312 (2013).

109. BirdLife International, www.birdlife.org/.

110. U. Roll, A. Feldman, M. Novosolov, A. Allison, A. M. Bauer, R. Bernard, M. Böhm, F. Castro-Herrera, L. Chirio, B. Collen, G. R. Colli, L. Dabool, I. Das, T. M. Doan, L. L. Grismer, M. Hoogmoed, Y. Itescu, F. Kraus, M. LeBreton, A. Lewin, M. Martins, E. Maza, D. Meirte, Z. T. Nagy, C. De, C. Nogueira, O. S. G. Pauwels, D. Pincheira-Donoso, G. D. Powney, R. Sindaco, O. J. S. Tallowin, O. Torres-Carvajal, J.-F. Trape, E. Vidan, P. Uetz, P. Wagner, Y. Wang, C. D. L. Orme, R. Grenyer, S. Meiri, The global distribution of tetrapods reveals a need for targeted reptile conservation. *Nat. Ecol. Evol.* **1**, 1677–1682 (2017).

111. Q. Huang, J. R. Sauer, A. Swatantran, R. Dubayah, A centroid model of species distribution with applications to the Carolina wren *Thryothorus ludovicianus* and house finch *Haemorhous mexicanus* in the United States. *Ecography* **39**, 54–66 (2016).

112. Y. Sun, Y. Deng, S. Yao, Y. Sun, A. A. Degen, L. Dong, J. Luo, S. Xie, Q. Hou, D. Tang, Y. Sun, J. Xiong, J. Peng, W. Hu, J. Ran, J. Deng, Distribution range and richness of plant species are predicted to increase by 2100 due to a warmer and wetter climate in Northern China. *Glob. Chang. Biol.* **31**, e70334 (2025).

113. R. Sokal, F. Rohlf, *Biometry: The Principles and Practice of Statistics in Biological Research* (W. H. Freeman & Company, ed. 2, 2012).

114. Z. Šidák, Rectangular confidence regions for the means of multivariate normal distributions. *J. Am. Stat. Assoc.* **62**, 626–633 (1967).

115. O. J. Dunn, Multiple comparisons among means. *J. Am. Stat. Assoc.* **56**, 52–64 (1961).

116. M. R. Moura, K. Ceron, J. J. M. Guedes, R. Chen-Zhao, Y. V. Sica, J. Hart, W. Dorman, J. M. Portmann, P. González-del-Pliego, A. Ranipeta, A. Catenazzi, F. P. Werneck, L. F. Toledo, N. S. Upham, J. F. R. Tonini, T. J. Colston, R. Guralnick, R. C. K. Bowie, R. A. Pyron, W. Jetz, A phylogeny-informed characterisation of global tetrapod traits addresses data gaps and biases. *PLoS Biol.* **22**, e3002658 (2024).

117. B. F. Oliveira, V. A. São-Pedro, G. Santos-Barrera, C. Penone, G. C. Costa, AmphiBIO, a global database for amphibian ecological traits. *Sci. Data* **4**, 170123 (2017).

118. J. P. Bird, R. Martin, H. R. Akçakaya, J. Gilroy, I. J. Burfield, S. T. Garnett, A. Symes, J. Taylor, Ç. H. Şekercioğlu, S. H. M. Butchart, Generation lengths of the world's birds and their implications for extinction risk. *Conserv. Biol.* **34**, 1252–1261 (2020).

119. C. D. Soria, M. Pacifici, M. Di Marco, S. M. Stephen, C. Rondinini, COMBINE: A coalesced mammal database of intrinsic and extrinsic traits. *Ecology* **102**, e03344 (2021).

120. J. A. Tobias, C. Sheard, A. L. Pigot, A. J. M. Devenish, J. Yang, F. Sayol, M. H. C. Neate-Clegg, N. Alioravainen, T. L. Weeks, R. A. Barber, P. A. Walkden, H. E. A. MacGregor, S. E. I. Jones, C. Vincent, A. G. Phillips, N. M. Marples, F. A. Montaño-Centellas, V. Leandro-Silva, S. Claramunt, B. Darski, B. G. Freeman, T. P. Bregman, C. R. Cooney, E. C. Hughes, E. J. R. Capp, Z. K. Varley, N. R. Friedman, H. Korntheuer, A. Corrales-Vargas, C. H.

Trisos, B. C. Weeks, D. M. Hanz, T. Töpfer, G. A. Bravo, V. Remeš, L. Nowak, L. S. Carneiro, A. J. Moncada R., B. Matysioková, D. T. Baldassarre, A. Martínez-Salinas, J. D. Wolfe, P. M. Chapman, B. G. Daly, M. C. Sorensen, A. Neu, M. A. Ford, R. J. Mayhew, L. F. Silveira, D. J. Kelly, N. N. D. Annorbah, H. S. Pollock, A. M. Grabowska-Zhang, J. P. McEntee, J. C. T. Gonzalez, C. G. Meneses, M. C. Muñoz, L. L. Powell, G. A. Jamie, T. J. Matthews, O. Johnson, G. R. R. Brito, K. Zyskowski, R. Crates, M. G. Harvey, M. J. Zevallos, P. A. Hosner, T. Bradfer-Lawrence, J. M. Maley, F. G. Stiles, H. S. Lima, K. L. Provost, M. Chibesa, M. Mashao, J. T. Howard, E. Mlamba, M. A. H. Chua, B. Li, M. I. Gómez, N. C. García, M. Päckert, J. Fuchs, J. R. Ali, E. P. Derryberry, M. L. Carlson, R. C. Urriza, K. E. Brzeski, D. M. Prawiradilaga, M. J. Rayner, E. T. Miller, R. C. K. Bowie, R.-M. Lafontaine, R. P. Scofield, Y. Lou, L. Somarathna, D. Lepage, M. Illif, E. L. Neuschulz, M. Templin, D. M. Dehling, J. C. Cooper, O. S. G. Pauwels, K. Analuddin, J. Fjeldså, N. Seddon, P. R. Sweet, F. A. J. DeClerck, L. N. Naka, J. D. Brawn, A. Aleixo, K. Böhning-Gaese, C. Rahbek, S. A. Fritz, G. H. Thomas, M. Schleuning, AVONET: Morphological, ecological and geographical data for all birds. *Ecol. Lett.* **25**, 581–597 (2022).

121. F. Sayol, J. P. Wayman, P. Dufour, T. E. Martin, J. P. Hume, M. W. Jørgensen, N. Martínez-Rubio, A. Sanglas, F. C. Soares, R. Cooke, C. D. Mendenhall, J. R. Margolis, J. C. Illera, R. Lemoine, E. Benavides, O. Lapiedra, K. A. Triantis, A. L. Pigot, J. A. Tobias, S. Faurby, T. J. Matthews, AVOTREX: A global dataset of extinct birds and their traits. *Glob. Ecol. Biogeogr.* **33**, e13927 (2024).

122. O. Oskyrko, C. Mi, S. Meiri, W. Du, ReptTraits: A comprehensive dataset of ecological traits in reptiles. *Sci. Data* **11**, 243 (2024).

123. G. Amatulli, S. Domisch, M.-N. Tuanmu, B. Parmentier, A. Ranipeta, J. Malczyk, W. Jetz, A suite of global, cross-scale topographic variables for environmental and biodiversity modeling. *Sci. Data* **5**, 180040 (2018).

124. D. N. Karger, O. Conrad, J. Böhner, T. Kawohl, H. Kreft, R. W. Soria-Auza, N. E. Zimmermann, H. P. Linder, M. Kessler, Climatologies at high resolution for the earth's land surface areas. *Sci. Data* **4**, 170122 (2017).

125. H. Mu, X. Li, Y. Wen, J. Huang, P. Du, W. Su, S. Miao, M. Geng, A global record of annual terrestrial Human Footprint dataset from 2000 to 2018. *Sci. Data* **9**, 176 (2022).

126. F. Gassert, O. Venter, J. E. M. Watson, S. P. Brumby, J. C. Mazzariello, S. C. Atkinson, S. Hyde, An operational approach to near real time global high resolution mapping of the terrestrial Human Footprint. *Front. Remote Sens.* **4**, 1130896 (2023).

127. E. E. Dyer, D. W. Redding, T. M. Blackburn, The global avian invasions atlas, a database of alien bird distributions worldwide. *Sci. Data* **4**, 170041 (2017).

128. D. Biancolini, V. Vascellari, B. Melone, T. M. Blackburn, P. Cassey, S. L. Scrivens, C. Rondinini, DAMA: The global distribution of alien mammals database. *Ecology* **102**, e03474 (2021).

129. D. Wang, Y. Zhao, S. Tang, X. Liu, W. Li, P. Han, D. Zeng, Y. Yang, G. Wei, Y. Kang, X. Si, Nearby large islands diminish biodiversity of the focal island by a negative target effect. *J. Anim. Ecol.* **92**, 492–502 (2023).

130. G. Ceballos, P. R. Ehrlich, R. Dirzo, Biological annihilation via the ongoing sixth mass extinction signaled by vertebrate population losses and declines. *Proc. Natl. Acad. Sci. U.S.A.* **114**, E6089–E6096 (2017).

131. J. Liu, F. Slik, S. Zheng, D. B. Lindenmayer, Undescribed species have higher extinction risk than known species. *Conserv. Lett.* **15**, e12876 (2022).

132. A. D. Davidson, M. J. Hamilton, A. G. Boyer, J. H. Brown, G. Ceballos, Multiple ecological pathways to extinction in mammals. *Proc. Natl. Acad. Sci. U.S.A.* **106**, 10702–10705 (2009).

133. J. Yang, C. Yang, H. Lin, A. C. Lees, J. A. Tobias, Elevational constraints on flight efficiency shape global gradients in avian wing morphology. *Curr. Biol.* **35**, 1890–1900.e5 (2025).

134. B. E. Crowley, L. R. Godfrey, R. J. Bankoff, G. H. Perry, B. J. Culleton, D. J. Kennett, M. R. Sutherland, K. E. Samonds, D. A. Burney, Island-wide aridity did not trigger recent megafaunal extinctions in Madagascar. *Ecography* **40**, 901–912 (2017).

135. S. van Buuren, K. Groothuis-Oudshoorn, mice: Multivariate imputation by chained equations in R. *J. Stat. Softw.* **45**, 1–67 (2011).

136. R. Molina-Venegas, How to get the most out of phylogenetic imputation without abusing it. *Methods Ecol. Evol.* **15**, 456–463 (2024).

137. P. Li, E. A. Stuart, D. B. Allison, Multiple imputation: A flexible tool for handling missing data. *JAMA* **314**, 1966–1967 (2015).

138. J. Stiller, S. Feng, A.-A. Chowdhury, I. Rivas-González, D. A. Duchêne, Q. Fang, Y. Deng, A. Kozlov, A. Stamatakis, S. Claramunt, J. M. T. Nguyen, S. Y. W. Ho, B. C. Faircloth, J. Haag, P. Houde, J. Cracraft, M. Balaban, U. Mai, G. Chen, R. Gao, C. Zhou, Y. Xie, Z. Huang, Z. Cao, Z. Yan, H. A. Ogilvie, L. Nakhleh, B. Lindow, B. Morel, J. Fjeldså, P. A. Hosner, R. R. Da Fonseca, B. Petersen, J. A. Tobias, T. Székely, J. D. Kennedy, A. H. Reeve, A. Liker, M. Stervander, A. Antunes, D. T. Tietze, M. F. Bertelsen, F. Lei, C. Rahbek, G. R. Graves, M. H. Schierup, T. Warnow, E. L. Braun, M. T. P. Gilbert, E. D. Jarvis, S. Mirarab, G. Zhang, Complexity of avian evolution revealed by family-level genomes. *Nature* **629**, 851–860 (2024).

139. N. S. Upham, J. A. Esselstyn, W. Jetz, Inferring the mammal tree: Species-level sets of phylogenies for questions in ecology, evolution, and conservation. *PLoS Biol.* **17**, e3000494 (2019).

140. N. Cox, B. E. Young, P. Bowles, M. Fernandez, J. Marin, G. Rapacciuolo, M. Böhm, T. M. Brooks, S. B. Hedges, C. Hilton-Taylor, M. Hoffmann, R. K. B. Jenkins, M. F. Tognelli, G. J. Alexander, A. Allison, N. B. Ananjeva, M. Auliya, L. J. Avila, D. G. Chapple, D. F. Cisneros-Heredia, H. G. Cogger, G. R. Colli, A. de Silva, C. C. Eisemberg, J. Els, A. Fong G., T. D. Grant, R. A. Hitchmough, D. T. Iskandar, N. Kidera, M. Martins, S. Meiri, N. J. Mitchell, S. Molur, C. de C. Nogueira, J. C. Ortiz, J. Penner, A. G. J. Rhodin, G. A. Rivas, M.-O. Rödel, U. Roll, K. L. Sanders, G. Santos-Barrera, G. M. Shea, S. Spawls, B. L. Stuart, K. A. Tolley, J.-F. Trape, M. A. Vidal, P. Wagner, B. P. Wallace, Y. Xie, A global reptile assessment highlights shared conservation needs of tetrapods. *Nature* **605**, 285–290 (2022).

141. L. J. Revell, phytools: An R package for phylogenetic comparative biology (and other things). *Methods Ecol. Evol.* **3**, 217–223 (2012).

142. L. si Tung Ho, C. Ané, A linear-time algorithm for gaussian and non-gaussian trait evolution models. *Syst. Biol.* **63**, 397–408 (2014).

143. J. A. Britnell, Y. Zhu, G. I. H. Kerley, S. Shultz, Ecological marginalization is widespread and increases extinction risk in mammals. *Proc. Natl. Acad. Sci. U.S.A.* **120**, e2205315120 (2023).

144. IUCN, Guidelines for using the IUCN red list categories and criteria, version 16 (2024); www.iucnredlist.org/resources/redlistguidelines.

145. R. P. Powers, W. Jetz, Global habitat loss and extinction risk of terrestrial vertebrates under future land-use-change scenarios. *Nat. Clim. Chang.* **9**, 323–329 (2019).

146. A. Purvis, J. L. Gittleman, G. Cowlishaw, G. M. Mace, Predicting extinction risk in declining species. *Proc. R. Soc. B. Biol. Sci.* **267**, 1947–1952 (2000).

147. L. Souviron-Priego, A. L. Márquez, N. Korbee, F. L. Figueroa, R. Real, Understanding the invasion of the macroalga *Rugulopteryx okamurae* (Ochrophyta) in the northern Alboran Sea through the use of biogeographic models. *Sci. Total Environ.* **955**, 176851 (2024).

148. G. Horváth, L. Z. Garamszegi, G. Herczeg, Phylogenetic meta-analysis reveals system-specific behavioural type–behavioural predictability correlations. *R. Soc. Open Sci.* **10**, 230303 (2023).

149. C. Spearman, The proof and measurement of association between two things. *Am. J. Psychol.* **15**, 72 (1904).

150. M. R. E. Symonds, A. Moussalli, A brief guide to model selection, multimodel inference and model averaging in behavioural ecology using Akaike's information criterion. *Behav. Ecol. Sociobiol.* **65**, 13–21 (2011).

151. K. P. Burnham, D. R. Anderson, Eds., *Model Selection and Multimodel Inference* (Springer, 2004).

152. D. B. Rubin, *Multiple Imputation for Nonresponse in Surveys* (Wiley, ed. 1, 1987).

153. K. Bartoń, MuMIn: Multi-model inference, version 1.48.11 (2025); https://cran.r-project.org/web/packages/MuMIn/index.html.

154. A. R. Ives, T. Garland Jr., Phylogenetic logistic regression for binary dependent variables. *Syst. Biol.* **59**, 9–26 (2010).

155. T. B. Atwood, S. A. Valentine, E. Hammill, D. J. McCauley, E. M. P. Madin, K. H. Beard, W. D. Pearse, Herbivores at the highest risk of extinction among mammals, birds, and reptiles. *Sci. Adv.* **6**, eabb8458 (2020).

156. R. J. Hijmans, M. Barbosa, R. Bivand, A. Brown, M. Chirico, E. Cordano, K. Dyba, E. Pebesma, B. Rowlingson, M. D. Sumner, terra: Spatial data analysis, version 1.8-93 (2026); https://cran.r-project.org/web/packages/terra/index.html.

157. G. Kalinkat, S. C. Jähnig, J. M. Jeschke, Exceptional body size–extinction risk relations shed new light on the freshwater biodiversity crisis. *Proc. Natl. Acad. Sci. U.S.A.* **114**, E10263–E10264 (2017).

158. P. M. Lucas, M. Di Marco, V. Cazalis, J. Luedtke, K. Neam, M. H. Brown, P. F. Langhammer, G. Mancini, L. Santini, Using comparative extinction risk analysis to prioritize the IUCN Red List reassessments of amphibians. *Conserv. Biol.* **38**, e14316 (2024).

159. S. Ducatez, R. Tingley, R. Shine, Using species co-occurrence patterns to quantify relative habitat breadth in terrestrial vertebrates. *Ecosphere* **5**, 1–12 (2014).

160. G. Murali, R. Gumbs, S. Meiri, U. Roll, Global determinants and conservation of evolutionary and geographic rarity in land vertebrates. *Sci. Adv.* **7**, eabe5582 (2021).

161. M. R. Cardoso, A. M. Bastidas-Urrutia, K. Frac, C. Hof, H. Kreft, J. Albrecht, K. Böhning-Gaese, S. A. Fritz, Urbanisation is related to the prevalence of threatened species on islands across the globe. *Glob. Ecol. Biogeogr.* **34**, e70125 (2025).

162. T. S. Doherty, A. S. Glen, D. G. Nimmo, E. G. Ritchie, C. R. Dickman, Invasive predators and global biodiversity loss. *Proc. Natl. Acad. Sci. U.S.A.* **113**, 11261–11265 (2016).
